# Supplementary material for: Compositional boundary layers trigger liquid unmixing in a basaltic crystal mush
Source: Nat Commun. 2019 Oct 23;10:4821. doi: 10.1038/s41467-019-12694-5 (PMC6811629; doi:10.1038/s41467-019-12694-5)
Supplement: Supplementary file 1 — Supplementary Information [file 41467_2019_12694_MOESM1_ESM.pdf]

SUPPLEMENTARY INFORMATION FOR

COMPOSITIONAL BOUNDARY LAYERS TRIGGER LIQUID UNMIXING IN A BASALTIC CRYSTAL MUSH

BY HONOUR ET AL.

SUPPLEMENTARY FIGURES

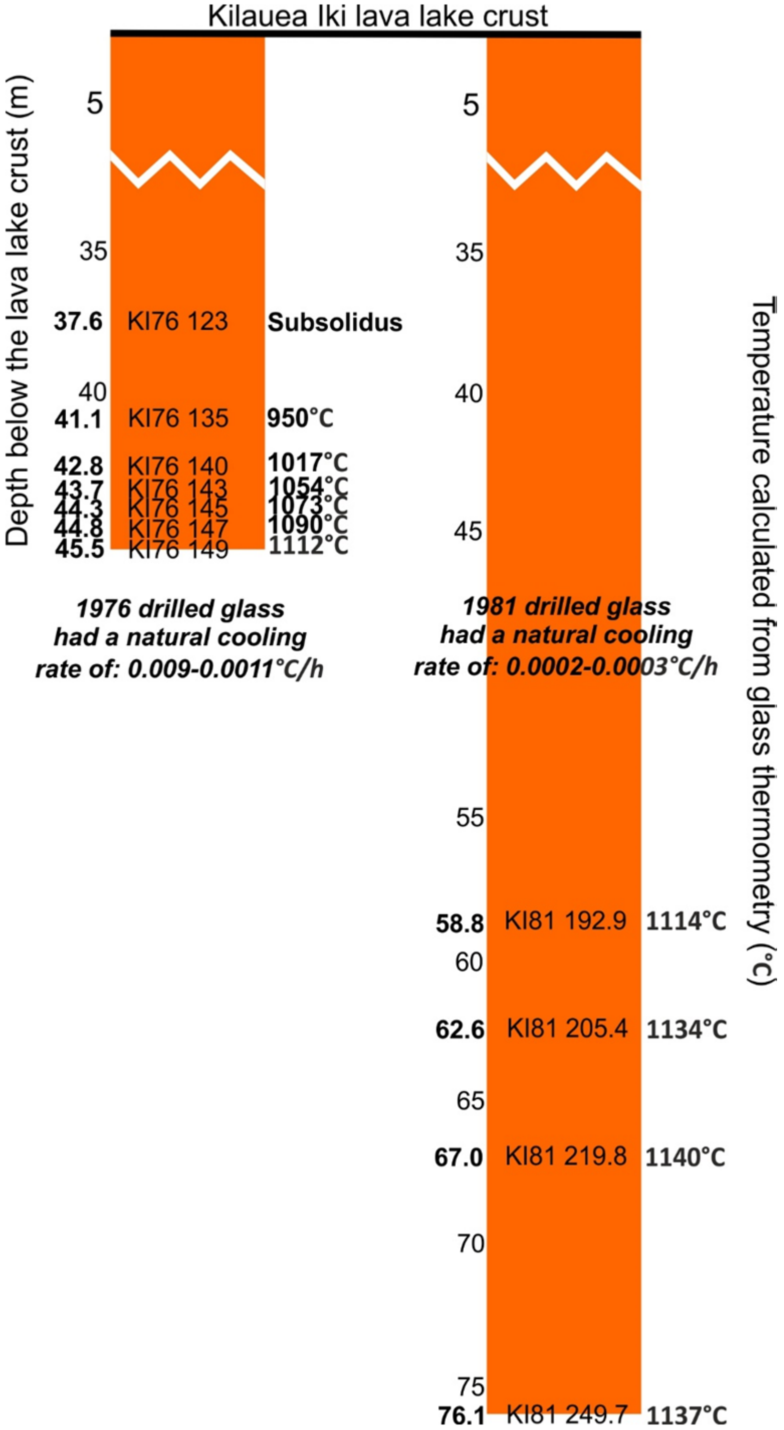

Supplementary Figure 1 Depth and corresponding temperature immediately prior to drilling of the Kīlauea Iki lava lake sample suite from the 1976 and 1981 drill cores. As the Kīlauea Iki lava lake cooled, the isotherms in the lava lake crust moved downwards at a progressively slower rate[1,2] Consequently, the samples in the two drill cores each experienced a different cooling rate.

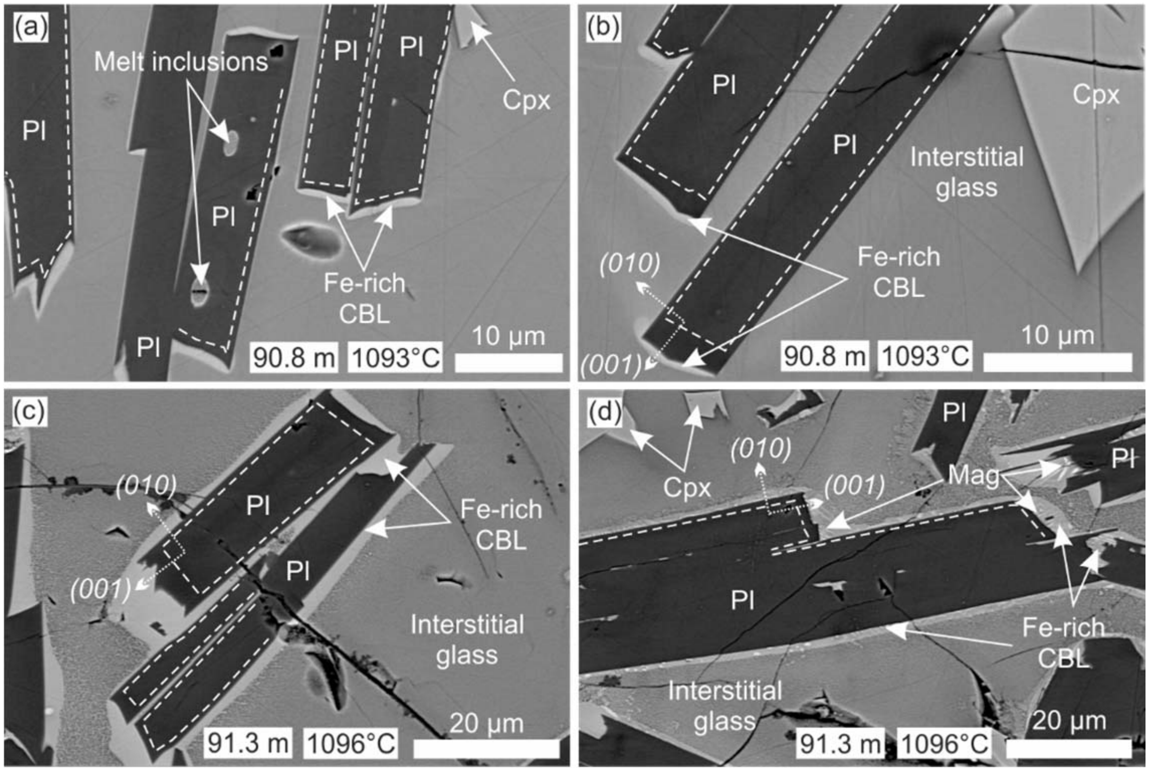

Supplementary Figure 2 The physical behaviour of immiscible liquids from the naturally quenched Snake River Plain basaltic flow, USA, sampled from the Sugar City Exploration Well, Idaho. (a) Plagioclase (Pl) laths surrounded by an Fe-rich compositional boundary layer (CBL). Note the melt inclusions hosting a nanoemulsion of immiscible liquids, the sample underwent the glass transition at 1093°C, thus preserving the residual liquid as glass. Temperature is determined by glass thermometry (see Methods). The Fe-rich CBL is thicker in the fastest growth direction of the plagioclase on the (001) face. (b) Plagioclase laths surrounded by an Fe-rich CBL representative of the residual liquid at 1093°C. Note the darker (i.e. more silicic) glass surrounding the pyroxene (Cpx). (c) Plagioclase laths surrounded by an Fe-rich CBL, representative of the residual liquid at 1096°C. (d) Plagioclase laths surrounded by an Fe-rich CBL, representative of the residual liquid at 1096°C. Note the magnetite crystals nucleated on the plagioclase surface and growing through the Fe-rich CBL.

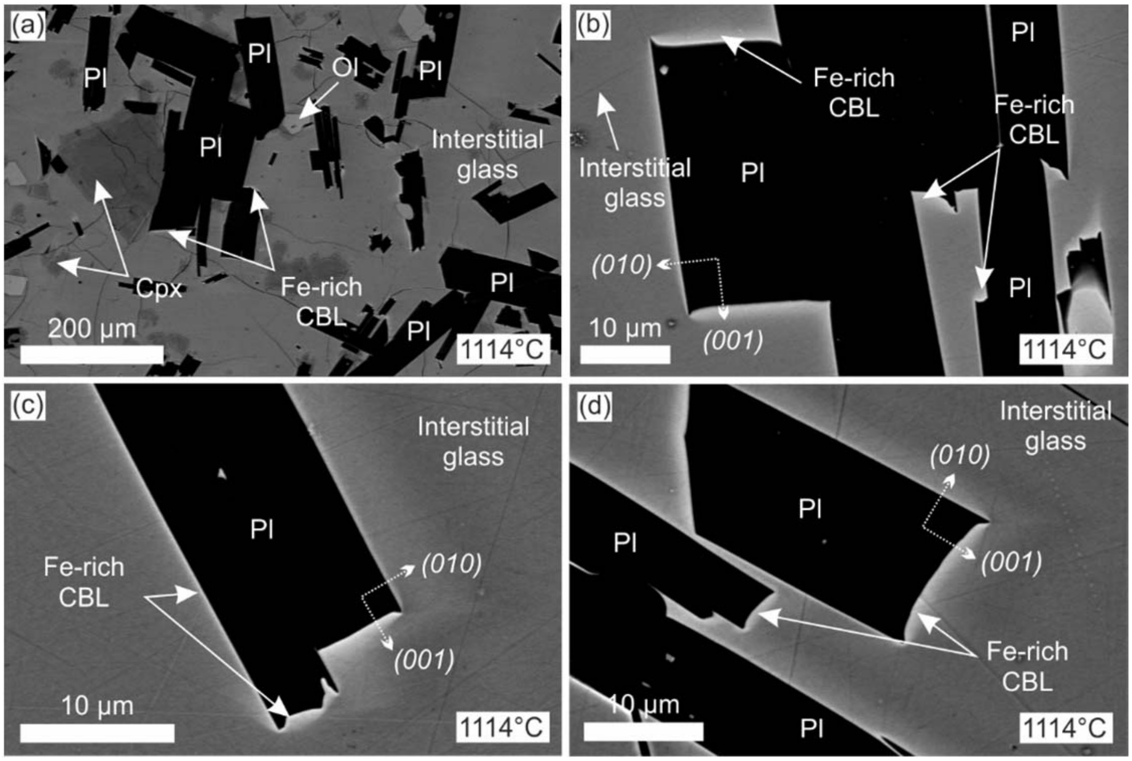

Supplementary Figure 3 The physical behaviour of immiscible liquids from the Laki eruption, Iceland, preserved by natural quench. (a) An overview of the sample, showing plagioclase (Pl), clinopyroxene (Cpx) and olivine (Ol), representative of the residual liquid at 1114°C. (b), (c), (d) Plagioclase laths surrounded by a diffuse Fe-rich compositional boundary layer (CBL) representative of the residual liquid at 1114°C; the Fe-rich CBL is thicker in the fastest growth direction of the plagioclase on the (001) face.

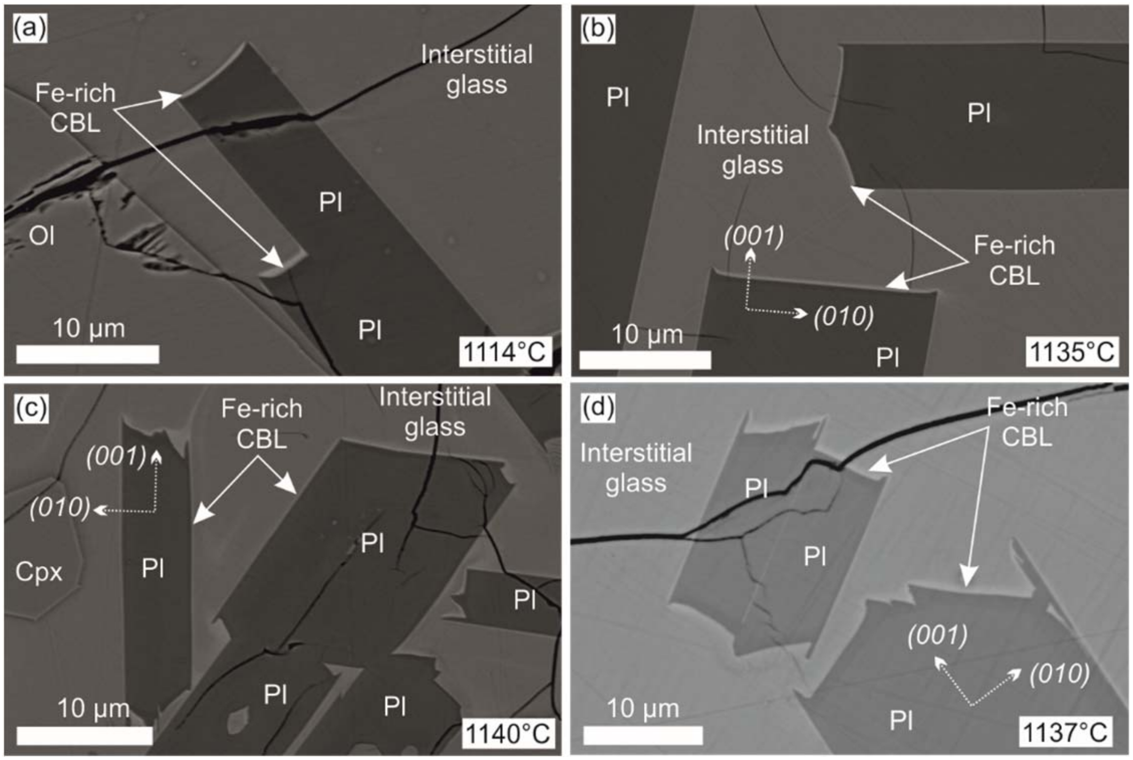

Supplementary Figure 4 The physical behaviour of immiscible liquids from the 1981 drill core from the Kīlauea Iki lava lake. (a) BSE image of plagioclase (Pl), and olivine (Ol), surrounded by interstitial glass with an Fe-rich compositional boundary layer (CBL) around the plagioclase grain, quenched from 1114°C during drilling. (b) Well-formed plagioclase grains, surrounded by an Fe-rich CBL. The Fe-rich CBL is thicker in the fastest growth direction of the plagioclase on the (001) face. Note the homogeneity of the interstitial glass, quenched from 1135°C during drilling. (c) Plagioclase laths surrounded by an Fe-rich CBL and clinopyroxene (Cpx) surrounded by a Si-rich compositional boundary layer, quenched from 1140°C during drilling. (d) Plagioclase grains with swallow-tail terminations extending from the (001) face. The plagioclase grains are surrounded by an Fe-rich CBL, within the interstitial glass quenched from 1137°C during drilling.

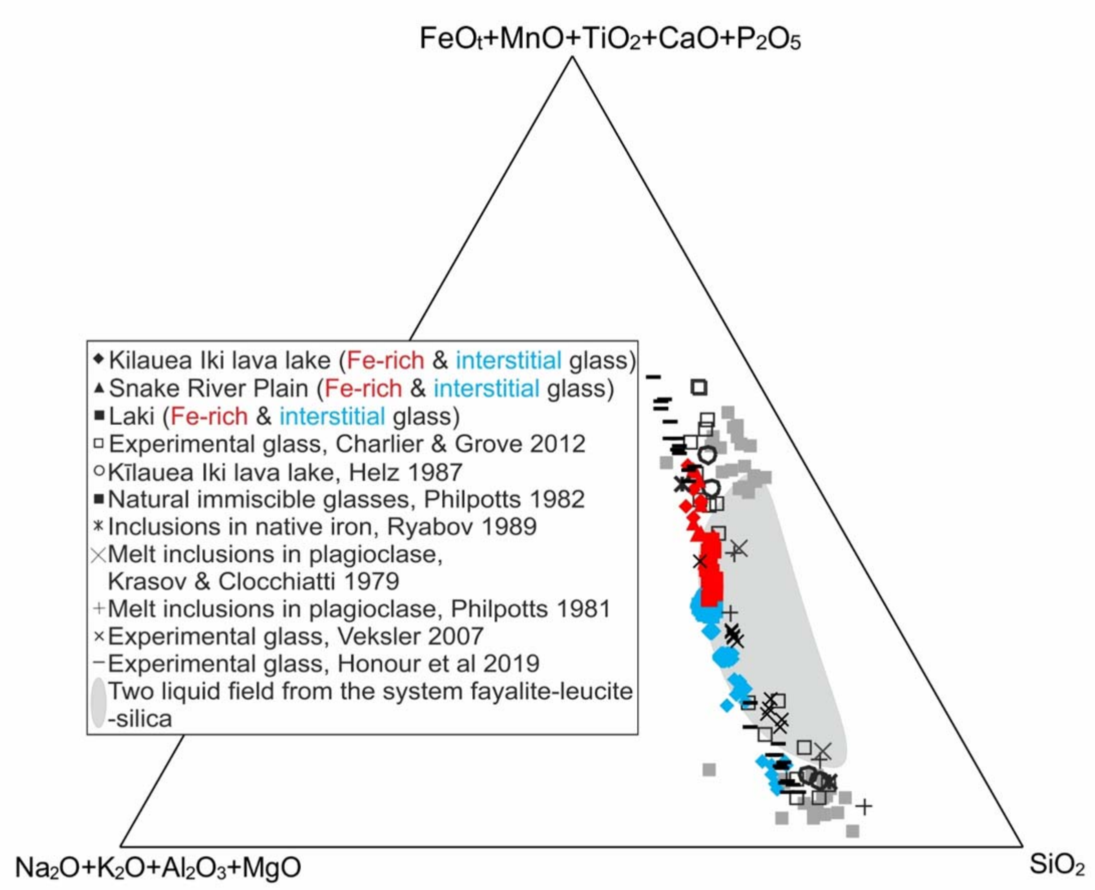

Supplementary Figure 5 Plot of the composition of Fe-rich and Si-rich immiscible liquids preserved as glassy droplets in a range of tholeiitic volcanic rocks, melt inclusions, and experiments. The light grey area is the two-liquid field from the fayalite-leucite-silica system. EPMA compositions determined for this study are overlaid; the Fe-rich compositions are plotted in red, while the surrounding interstitial glass is plotted in blue[3-9].

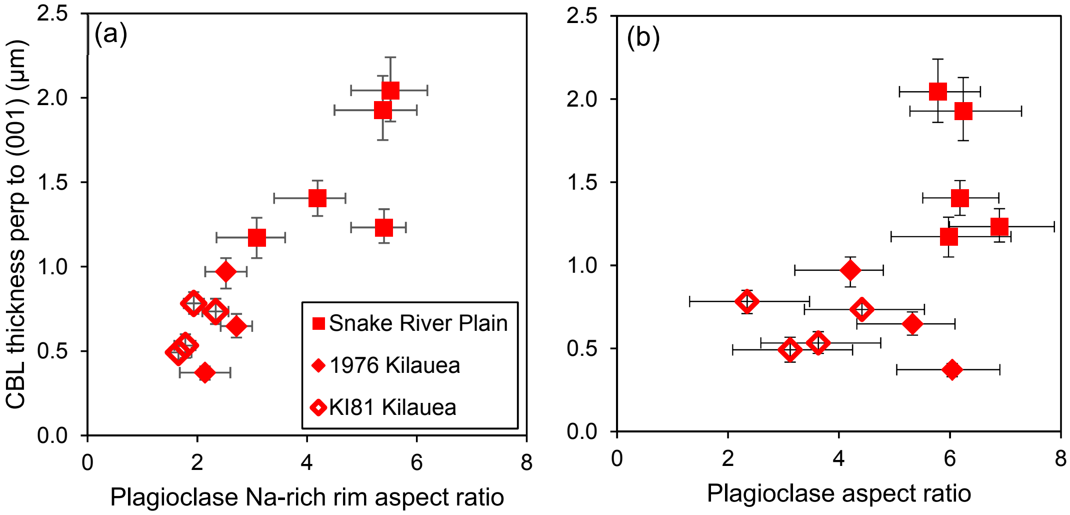

Supplementary Figure 6 (a) The average aspect ratio of the Na-rich rims on the plagioclase compared with the thickness of the Fe-rich compositional boundary layer (CBL) perpendicular to (001) crystal face for samples from Snake River Plain (SRP), and the 1976 and 1981 Kīlauea Iki lava lake drill cores. (b) Average overall aspect ratio of plagioclase grains against Fe-rich compositional boundary layer (CBL) perpendicular to (001) crystal face. Uncertainties were determined by the bootstrap method. This data is plotted from Supplementary Table 2.

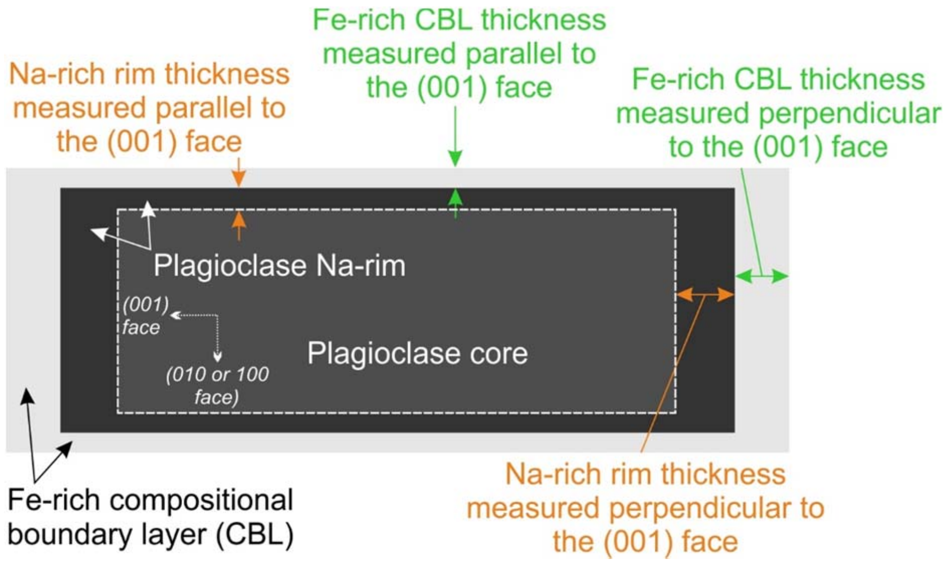

Supplementary Figure 7 A schematic diagram showing a plagioclase grain surrounded by an Fe-rich compositional boundary layer.

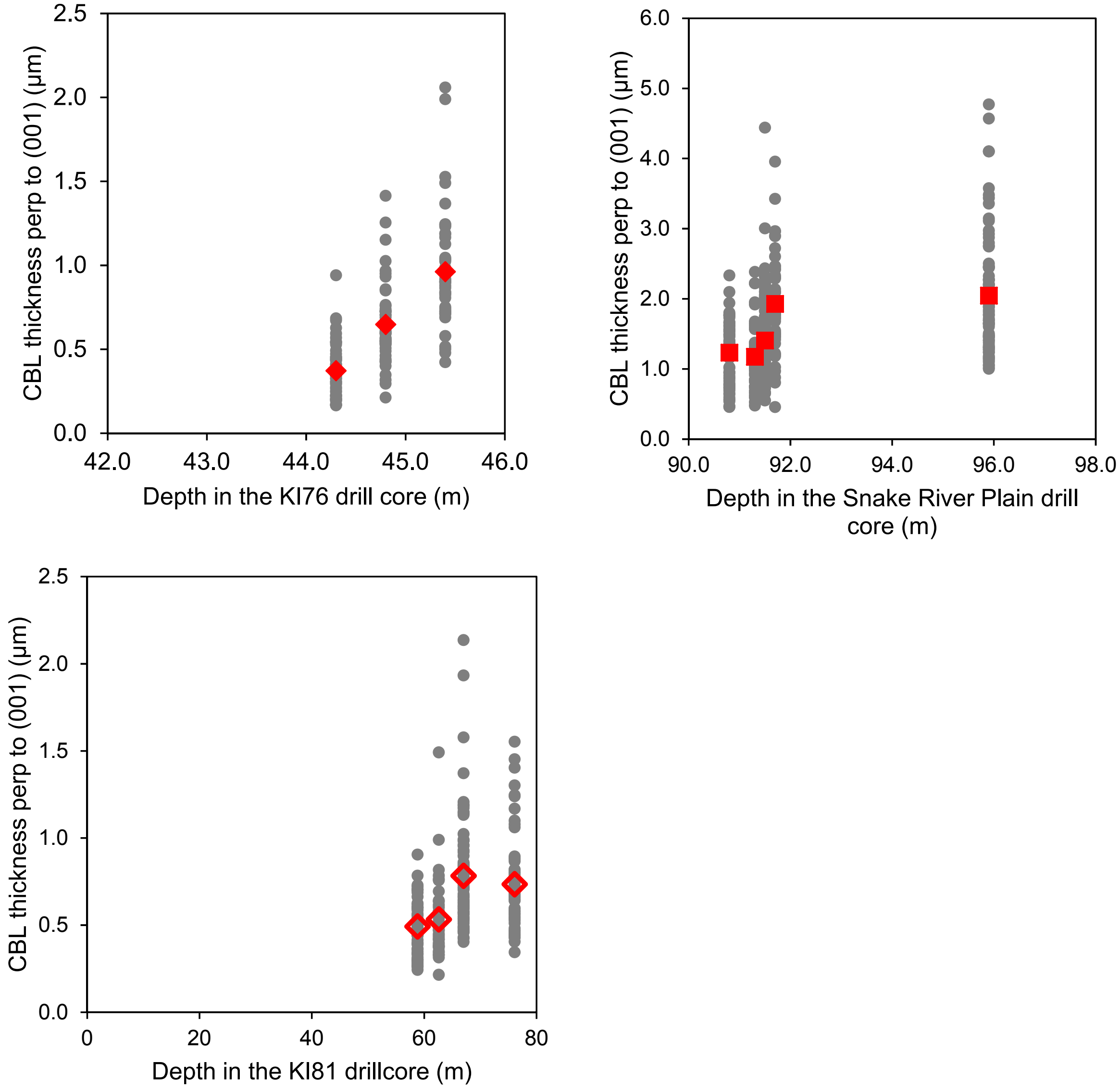

Supplementary Figure 8 (Upper left) The thickness of the Fe-rich compositional boundary layer (CBL) perpendicular to the plagioclase (001) crystal face as a function of depth in the Kīlauea Iki lava lake 1976 drill core. (Upper right) The thickness of the Fe-rich CBL perpendicular to plagioclase (001) crystal faces as a function of depth in the Snake River Plain drill core. (Lower left) The thickness of the Fe-rich CBL perpendicular to plagioclase (001) crystal faces as a function of depth in the Kīlauea Iki lava lake 1981 drill core. This data is plotted from Supplementary Table 2. Grey circles show individual data points and red symbols are average values.

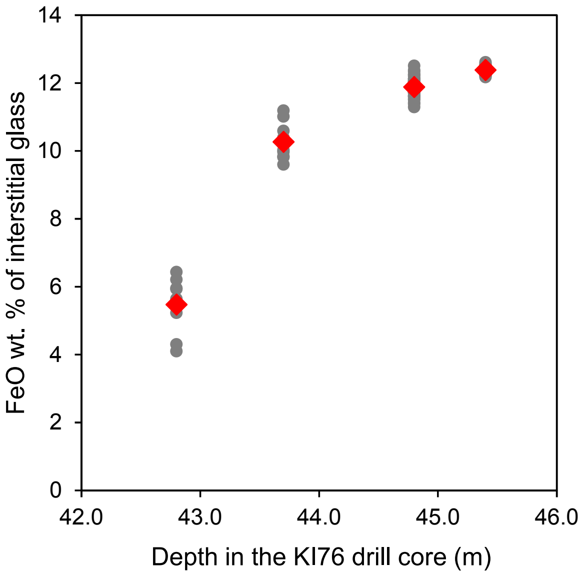

Supplementary Figure 9 The FeO_{total} concentration (wt.%) of interstitial glass as a function of depth in the Kīlauea Iki lava lake 1976 drill core (the data is plotted from Supplementary Table 1). Grey circles are individual data points and red diamonds are average values.

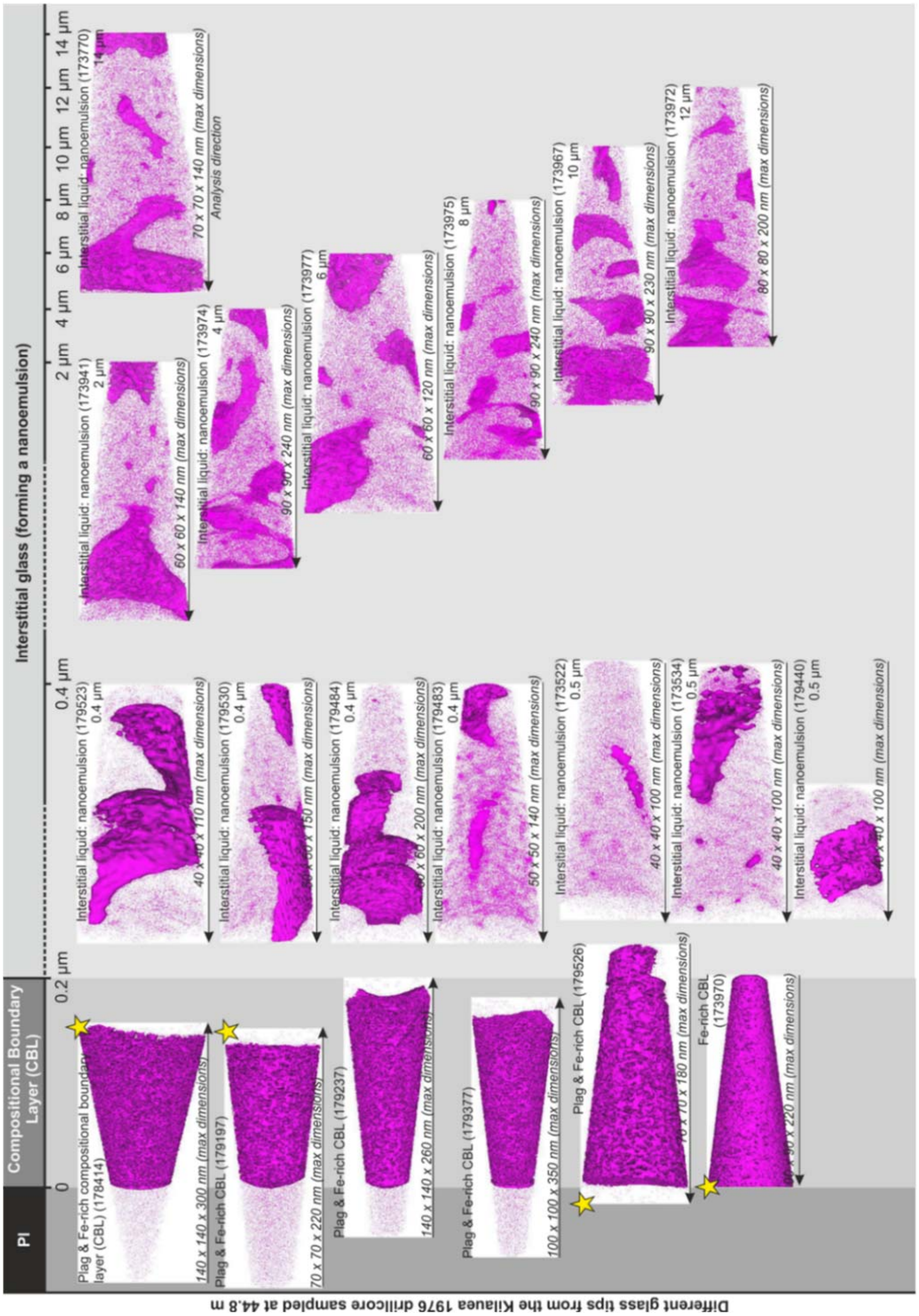

Supplementary Figure 10 Three-dimensional reconstruction of APT data from twenty glass tips analysed across a plagioclase grain, its adjacent Fe-rich CBL and the surrounding interstitial liquid (comprised of a nanoemulsion) in a sample from Kīlauea Iki lava lake 1976 drill core, quenched from ~1090°C during drilling at a depth of 44.8 m. Each dot represents a single atom, but not all atoms are shown. The yellow star denotes samples shown in Supplementary Figures 11–12.

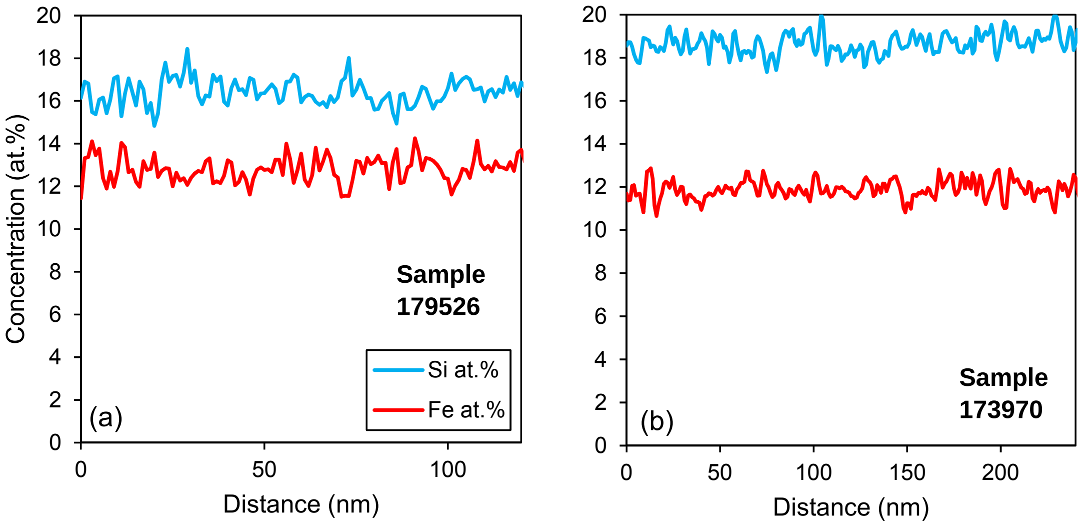

Supplementary Figure 11 APT relative compositional data for sample (a) 179526, (b) 173970, for Si at.% and Fe at.% across the Fe-rich compositional boundary layer (CBL) sampled by those glass tips in Supplementary Figure 10 that are marked with a yellow star. The two samples are taken from the Kīlauea Iki lava lake 1976 drill core, quenched from ~1090°C during drilling at a depth of 44.8 m. The standard deviations across the Fe-rich CBL for each are 0.6 and 0.6, respectively. Data points are plotted at 1 nm distances; however, for clarity the data markers have been removed.

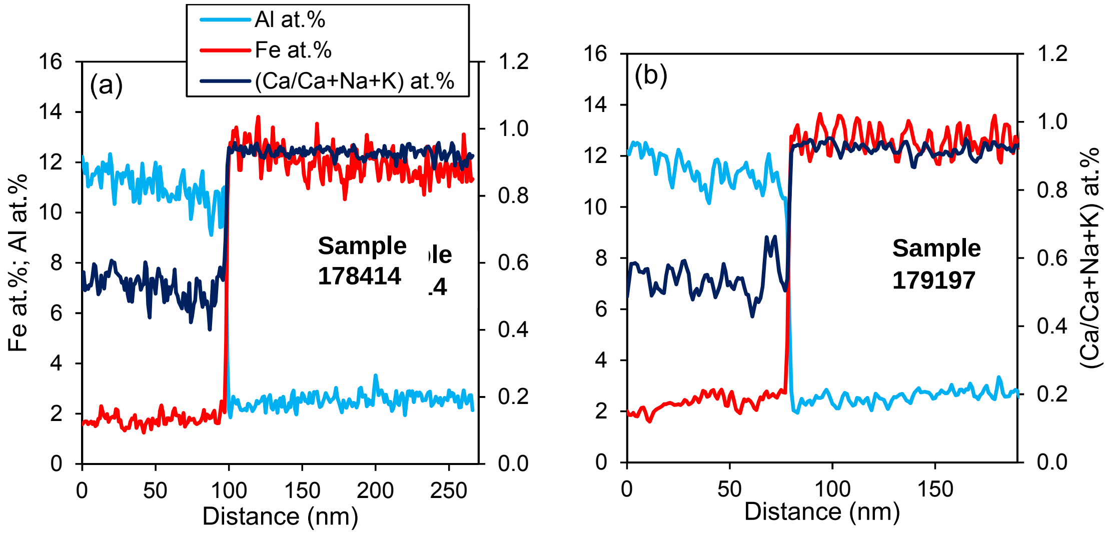

Supplementary Figure 12 APT relative compositional data for sample (a) 178414, (b) 179197, for Al at.%, Fe at.%, and (Ca/Ca+Na+K) at.% across the margin of a plagioclase grain surrounded by an Fe-rich CBL – see glass tips in Supplementary Figure 10 that are marked with a yellow star. Sample is taken from the Kīlauea Iki lava lake 1976 drill core, quenched from ~1090°C during drilling at a depth of 44.8 m. The standard deviations across plagioclase for each are 0.6, 0.3, and 0.05, respectively. The respective standard deviations across the Fe-rich CBL for each are 0.4, 0.6, and 0.05. Data points are plotted at 1 nm distances; however, for clarity the data markers have been removed.

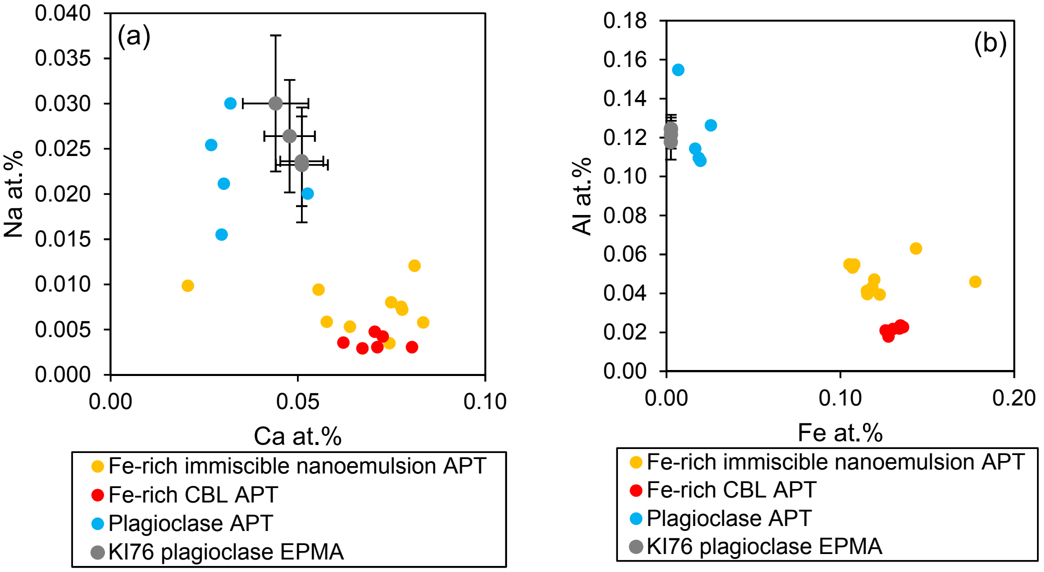

Supplementary Figure 13 (a) Relative APT compositions of plagioclase, its Fe-rich compositional boundary layer (CBL), and the Fe-rich immiscible liquid of the surrounding nanoemulsion. (b) Relative APT compositions of plagioclase, its Fe-rich CBL, and the Fe-rich immiscible liquid of the surrounding nanoemulsion. The data are plotted from Supplementary Table 1 and 4. The sample is from the Kīlauea Iki lava lake 1976 drill core, quenched from ~1090°C during drilling at 44.8 m. EPMA analyses of plagioclase are shown with two standard deviation errors.

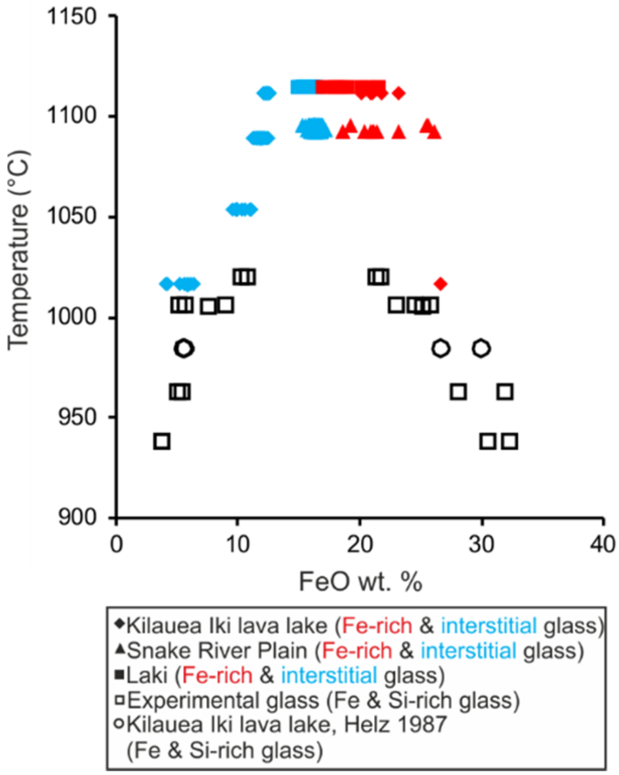

Supplementary Figure 14 Quench temperature as a function of the glass FeO wt.% for samples from the Kīlauea Iki lava lake, and the Snake River Plain and Laki lava flows, compared to previously published data. This shows the same trends as Fig. 5, in which quench temperature is plotted against the NBO/T of the analysed glass. Note that we find evidence of unmixing in the form of well-defined compositional boundary layers surrounding plagioclase, at much higher temperatures than the experimental determinations of unmixing[3,4].

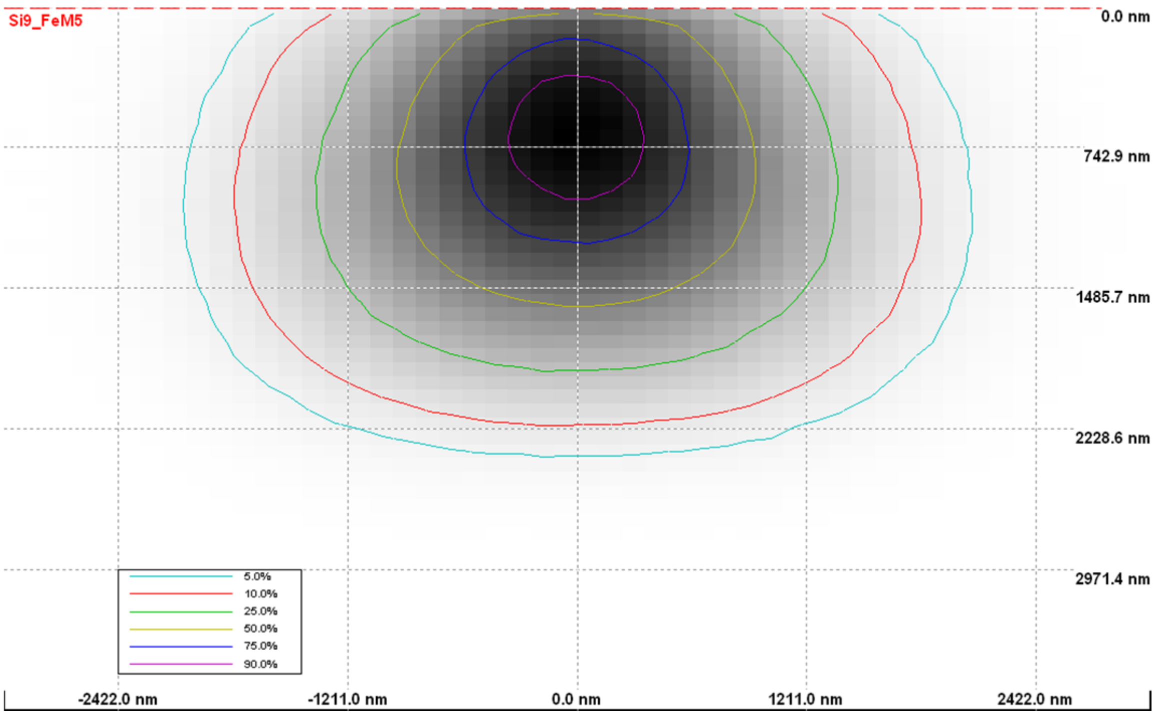

Supplementary Figure 15 The interaction volume of a 2 μm, 15 kV focused electron beam interacting with an Fe-rich homogeneous glass, calculated using *Casino v2.48* software.

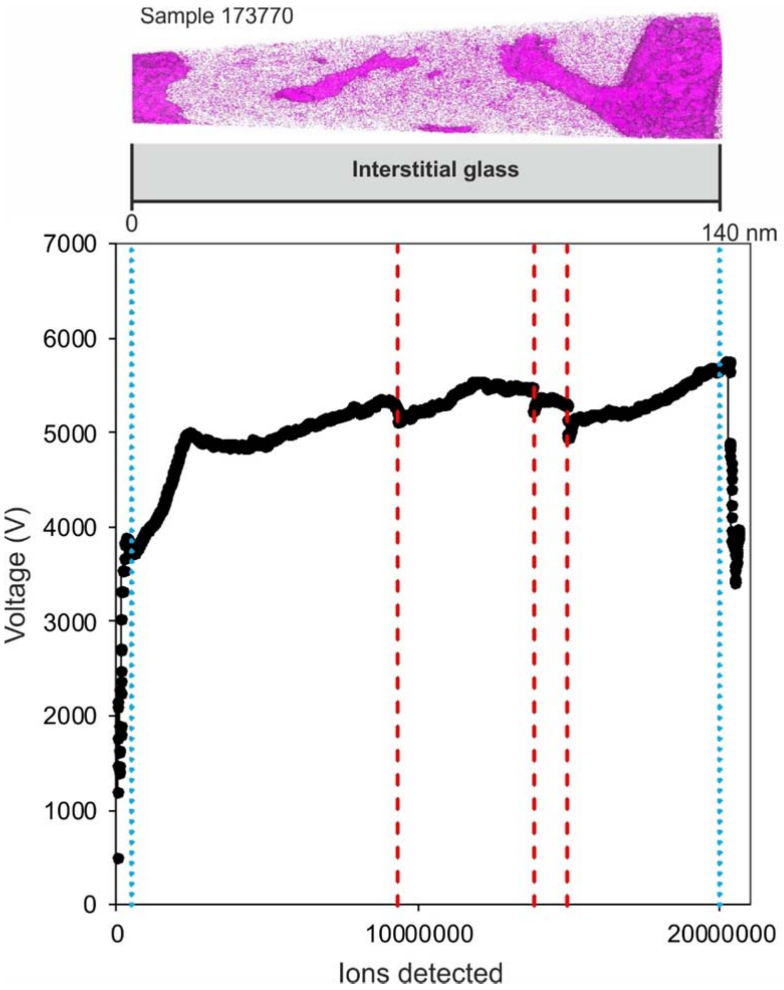

Supplementary Figure 16 APT voltage curve for the APT tip, sample 173770, cut 14 µm from the Fe-rich compositional boundary layer of a plagioclase in the Kīlauea Iki lava lake drill core from 1976, from the polished section quenched from ~1090°C during drilling at a depth of 44.8 m. Red dashed lines show noticeable voltage drops. Blue dotted lines show the region within which sufficiently stable ion evaporation occurred, before failure.

The figure represents voltage evolution typical for the many analyses collected as part of this report (173770). Approximately 20.4 million ions were collected before specimen failure (indicated by the sharp voltage drop at the end of the plot). Only ions acquired after sufficiently stable ion evaporation and before failure (~0.5-20.0 million ions) were used for reconstruction and subsequent chemical analysis. The voltage trends were generally smooth, indicating stable analysis. The general gentle increase in voltage is related to the increase in the cross-section of the atom probe tip. However, there were occasions with noticeable voltage drops during analysis, e.g. at 9.3, 13.8, and 14.9 million ions (red dashed lines). These voltage drops are the instrument's response to increases in detected ion rates. For example, the evaporation at 14.9 million ions briefly went for 1.0+-0.3% to ~7% causing this instrument response. The control algorithm adjusts voltage to maintain a target, constant detected ion rate.

In general, significant ionization spikes may indicate uncontrolled removal of specimen material suggesting that either some small depth of material has been removed but not fully

detected (resulting in a discontinuity in the analysed structure), or just a normal response due to normal evaporation variations. Cases where significant losses of collected material have occurred are indicated when the previous, longer-scale voltage trends are not resumed (clear voltage discontinuity) and these regions were filtered out and not used as continuous volumes as part of this report. For cases like those shown in this figure, where the voltage trends were resumed, the reconstructed volumes are deemed robust. Only analysis identified as robust are used in this contribution.

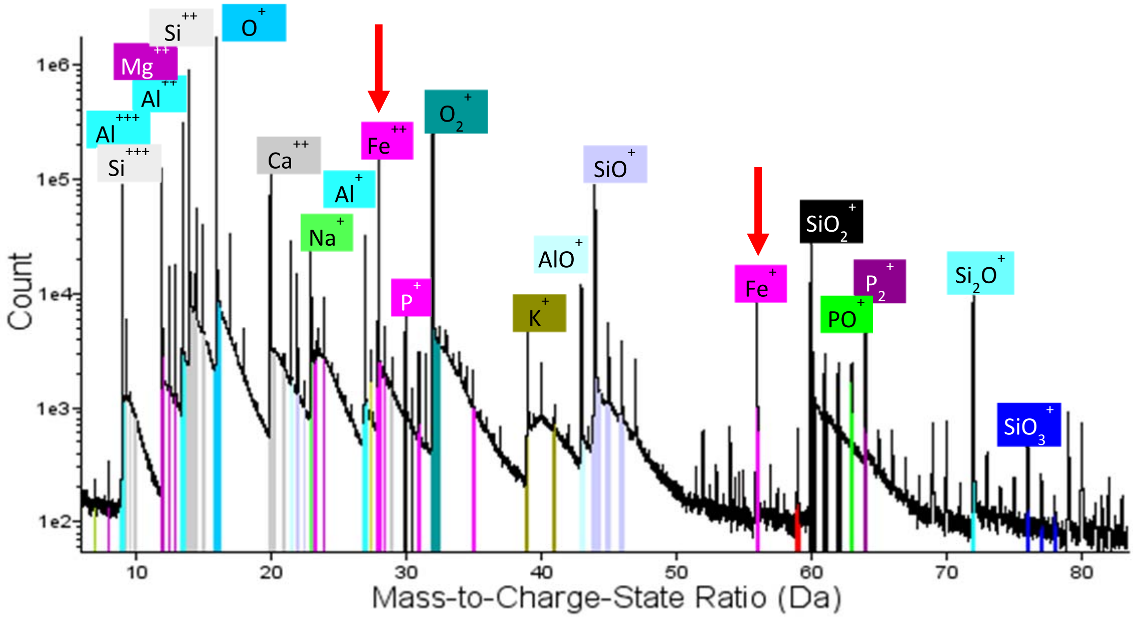

Supplementary Figure 17 APT mass ranging for sample 173770 at 14 μm from the Fe-rich compositional boundary layer of a plagioclase in the Kīlauea Iki lava lake drill core from 1976. Arrows highlight the Fe+1 and Fe+2 peaks.

Mass Ranging: Spectrum analysis features included in IVAS require manual assignment of mass-to-charge ion ranges to a particular element or multi-element ion. This figure shows log-scale mass spectrum from 173770 with the ranges used for the strongest peaks colour coded and labelled. For multi-isotope elements like silicon, the ratios of the ^{28}Si, ^{29}Si, and ^{30}Si are easy to identify and provide strong confidence for the identification of silicon ions and silicon-containing ions like SiO_2. For single isotope elements, the confidence depends on the likelihood of competing element combinations that could present an ion peak at the same position. The presence of multiple charge states can provide some additional material for excluding certain potential identification overlaps. Likewise, chemical material from other techniques can also be used to evaluate possible ion types. This log-scale plot shows many unidentified peaks, but these features contribute very little to the overall composition.

In particular peak overlaps have been considered. The major isotopes of Fe are 56, 54, 57, 58, so the +2 charge states are at 28, 27, 28.5, and 29 in order. There are expected overlaps both with ^{28}Si and ^{29}Si as well as ^{27}Al. If one assumes some sort of natural abundance, then there are clear mixes of these elements/isotopes in those peaks. When reporting average composition for some volume (freely selected by the analyst) one can use these natural abundances to estimate the individual contributions (overlaps), but this is not possible on a single ion basis as the statistics of many ions are needed to correct an

average composition for some sub-volume. For the figure below specifically, the intensity of the 29 peak suggests it is almost all silicon, since the ^{58}Fe abundance is relatively weak. Consequently, this suggests that ~20% of the $^{56}Fe^{++}$ peak is actually silicon. Also, about half of the ^{27}Fe peak is Al and half is $^{54}Fe^{++}$.

For Ca, almost all of the detected Ca is observed in the Ca++ charge state. There is some overlap of the $^{44}Ca^{++}$ with $^{28}Si^{16}O^{++}$ peak but it is dominated by SiO++, so is negligible. For Ca+, there are interferences and upon closer scrutiny, the ^{40}Ca and ^{42}Ca peaks are almost certainly MgO+ at nearly 100%. These two peaks account for 0.06 at.% of the composition i.e. there is not Ca+. This change would reduce the estimated Ca by about 2% (relative not absolute; this very small change is probably less than the estimated error on the average composition measurement) and increase Mg by about 2%, making this mislabelling statistically insignificant.

SUPPLEMENTARY TABLES

Table 1 *EPMA of glass in samples from the 1976 drill core from the Kīlauea Iki lava lake; Snake River Plain tholeiite in the USA and the Laki eruption in Iceland. The degree of polymerisation is parameterised as NBO/T, where T = Si + Al + P + Ti wt. %.*

Sample	Sample depth from core top (m)	Temperature °C (Putrika 2008)	SiO2	TiO2	Al2O3	FeO	MnO	MgO	CaO	Na2O	K2O	P2O5	Total	NBO/T	Density (g/m3)	Mg#/Fo#/An#
**KI76 149 Si-rich glass**	45.4	1112	50.00	5.37	12.34	12.57	0.17	4.29	7.81	1.96	1.15	0.80	96.45	0.61		
**KI76 149 Si-rich glass**	45.4	1112	49.66	5.23	11.88	12.61	0.19	4.54	7.92	2.05	1.19	0.78	96.05	0.65		
**KI76 149 Si-rich glass**	45.4	1112	50.03	5.31	12.16	12.51	0.21	4.66	7.82	1.94	1.24	0.83	96.71	0.63		
**KI76 149 Si-rich glass**	45.4	1112	50.11	5.31	12.27	12.43	0.19	4.49	7.86	1.90	1.19	0.80	96.58	0.62		
**KI76 149 Si-rich glass**	45.4	1112	49.79	5.28	12.05	12.18	0.18	4.59	7.80	1.99	1.19	0.81	95.85	0.63		
**KI76 149 Si-rich glass**	45.4	1112	49.22	5.24	12.18	12.23	0.21	4.49	7.80	1.90	1.24	0.80	95.30	0.62		
**KI76 149 Si-rich glass**	45.4	1112	50.17	5.33	12.17	12.28	0.17	4.57	7.82	2.13	1.14	0.80	96.59	0.62		
**KI76 149 Si-rich glass**	45.4	1112	49.89	5.29	12.43	12.21	0.19	4.53	7.98	2.03	1.22	0.80	96.57	0.62		
**KI76 149 Si-rich glass**	45.4	1112	50.35	5.30	12.12	12.46	0.17	4.52	7.90	2.02	1.18	0.80	96.81	0.63		
**KI76 149 Si-rich glass**	45.4	1112	50.22	5.26	12.37	12.54	0.18	4.56	7.93	2.04	1.20	0.78	97.07	0.63		
**KI76 149 Si-rich glass**	45.4	1112	49.95	5.33	12.03	12.32	0.18	4.62	8.02	2.01	1.20	0.80	96.47	0.64		
**KI76 149 Si-rich glass**	45.4	1112	50.02	5.23	12.28	12.51	0.18	4.41	7.89	2.53	1.16	0.79	97.01	0.63		
**KI76 149 Si-rich glass**	45.4	1112	50.12	5.30	12.17	12.30	0.19	4.38	7.89	2.62	1.14	0.79	96.91	0.63		
**KI76 149 Si-rich glass**	45.4	1112	50.05	5.30	11.96	12.45	0.17	4.44	7.90	2.48	1.15	0.80	96.70	0.64		
**KI76 149 Si-rich glass**	45.4	1112	49.82	5.28	12.17	12.45	0.19	4.28	7.99	2.51	1.14	0.81	96.64	0.63		
**KI76 149 Si-rich glass**	45.4	1112	49.83	5.24	12.14	12.27	0.19	4.46	7.99	2.43	1.13	0.80	96.50	0.64		
**KI76 149 Si-rich glass**	45.4	1112	49.87	5.27	12.17	12.25	0.18	4.38	7.97	2.56	1.13	0.79	96.57	0.63		
Averages	45.4	*1112*	*49.90 (0.3)*	*5.29 (0.1)*	*12.17 (0.1)*	*12.39 (0.1)*	*0.18 (0.1)*	*4.48 (0.1)*	*7.89 (0.1)*	*2.18 (0.3)*	*1.18 (0.1)*	*0.79 (0.1)*	-	*0.63*	*2.71*	-
**KI76 149 Fe-rich glass**	45.4	1112	42.27	9.64	5.73	20.15	0.30	8.11	10.03	1.12	0.45	1.40	99.20	1.34		
**KI76 149 Fe-rich glass**	45.4	1112	41.22	9.88	3.38	21.03	0.30	8.67	9.68	0.71	0.50	1.47	96.83	1.51		
**KI76 149 Fe-rich glass**	45.4	1112	39.51	10.13	4.21	21.79	0.33	8.00	10.25	1.03	0.19	1.55	96.99	1.52		
**KI76 149 Fe-rich glass**	45.4	1112	41.42	10.20	3.59	20.90	0.29	8.40	10.01	0.81	0.56	1.43	97.62	1.49		
**KI76 149 Fe-rich glass**	45.4	1112	37.68	11.05	2.91	23.19	0.36	8.57	10.71	0.86	0.12	1.62	97.06	1.73		
Averages			*40.42 (1.6)*	*10.18 (0.5)*	*3.96 (1.0)*	*21.41 (1.)*	*0.32 (0.02)*	*8.35 (0.3)*	*10.14 (0.3)*	*0.91 (0.1)*	*0.36 (0.2)*	*1.49 (0.1)*	-	*1.52*	*3.00*	
**KI76 149 Average major mineral phases**																
KI76 149 Plagioclase (8)	45.4	-	*48.08 (0.2)*	*0.13 (0.01)*	*30.46 (0.3)*	*0.94 (0.05)*	-	*0.10 (0.01)*	*15.12 (0.6)*	*2.80 (0.2)*	*0.12 (0.01)*	-	-	-	-	*83*
KI76 149 Olivine (8)	45.4	-	*37.53 (0.5)*	*0.23 (0.1)*	-	*22.71 (1.2)*	-	*38.11 (1.1)*	*0.19 (0.03)*	-	-	-	-	-	-	*57*
KI76 149 Pyroxene (8)	45.4	-	*50.544 (0.3)*	*9.10 (0.7)*	*2.27 (0.3)*	*7.15 (0.6)*	-	*16.29 (0.1)*	*12.67 (0.2)*	*0.11 (0.01)*	-	-	-	-	-	*64*
**KI76 147 Si-rich glass**	44.8	1090	53.77	4.01	12.62	11.74	0.17	3.76	7.02	3.18	1.61	0.91	98.79	0.56		
**KI76 147 Si-rich glass**	44.8	1090	53.36	4.07	12.50	12.02	0.19	3.77	6.95	3.00	1.63	0.91	98.40	0.56		

**KI76 147 Si-rich glass**	44.8	1090	54.03	3.98	12.43	11.76	0.18	3.61	6.91	2.39	1.58	0.87	97.74	0.53		
**KI76 147 Si-rich glass**	44.8	1090	54.76	4.07	12.36	11.39	0.18	3.50	6.86	2.97	1.60	0.91	98.60	0.53		
**KI76 147 Si-rich glass**	44.8	1090	53.60	4.07	12.24	12.05	0.19	3.63	7.24	3.32	1.52	0.89	98.76	0.58		
**KI76 147 Si-rich glass**	44.8	1090	55.28	4.11	12.36	12.38	0.21	3.63	7.01	2.18	1.55	0.94	99.64	0.53		
**KI76 147 Si-rich glass**	44.8	1090	54.47	4.09	12.70	12.08	0.31	3.84	6.96	3.09	1.58	0.95	100.06	0.56		
**KI76 147 Si-rich glass**	44.8	1090	53.75	4.03	12.38	11.29	0.14	3.61	6.90	3.22	1.55	0.86	97.75	0.54		
**KI76 147 Si-rich glass**	44.8	1090	54.37	4.00	12.61	12.51	0.18	3.74	6.97	2.85	1.55	0.92	99.68	0.56		
**KI76 147 Si-rich glass**	44.8	1090	54.76	4.07	12.74	11.82	0.07	3.53	7.11	3.26	1.62	0.92	99.89	0.54		
**KI76 147 Si-rich glass**	44.8	1090	54.98	4.06	12.49	11.53	0.17	3.43	6.91	2.43	1.54	0.91	98.45	0.51		
**KI76 147 Si-rich glass**	44.8	1090	54.76	4.07	12.43	12.05	0.20	3.68	6.89	3.14	1.60	0.89	99.69	0.55		
**KI76 147 Si-rich glass**	44.8	1090	54.04	3.98	12.99	12.12	0.21	3.69	6.80	3.02	1.60	0.96	99.40	0.54		
**KI76 147 Si-rich glass**	44.8	1090	54.49	4.06	12.61	11.79	0.20	3.67	6.93	2.60	1.53	0.92	98.79	0.53		
**KI76 147 Si-rich glass**	44.8	1090	54.52	3.98	12.81	11.90	0.13	3.47	7.03	3.20	1.58	0.93	99.54	0.54		
**KI76 147 Si-rich glass**	44.8	1090	54.23	3.86	12.14	11.42	0.27	3.73	6.95	3.31	1.56	0.93	98.41	0.56		
**KI76 147 Si-rich glass**	44.8	1090	53.72	3.91	12.59	12.24	0.22	3.48	6.72	2.63	1.66	0.91	98.08	0.54		
**KI76 147 Si-rich glass**	44.8	1090	53.92	3.87	12.66	11.90	0.30	3.70	6.77	2.54	1.65	0.93	98.24	0.54		
**KI76 147 Si-rich glass**	44.8	1090	54.40	3.86	12.60	11.75	0.18	3.82	6.96	3.32	1.65	0.88	99.42	0.56		
**KI76 147 Si-rich glass**	44.8	1090	54.83	3.88	12.54	11.80	0.00	3.54	6.99	2.35	1.56	0.90	98.39	0.52		
**KI76 147 Si-rich glass**	44.8	1090	54.50	3.92	12.62	11.95	0.15	3.67	7.01	2.84	1.68	0.89	99.22	0.54		
**KI76 147 Si-rich glass**	44.8	1090	54.84	3.98	13.01	12.12	0.20	3.72	6.88	2.99	1.56	0.93	100.23	0.54		
**KI76 147 Si-rich glass**	44.8	1090	54.07	4.00	12.58	11.91	0.05	3.63	6.96	2.29	1.61	0.89	98.00	0.53		
**KI76 147 Si-rich glass**	44.8	1090	54.25	3.86	12.48	11.71	0.24	3.63	6.77	3.14	1.65	0.90	98.64	0.54		
**KI76 147 Si-rich glass**	44.8	1090	54.22	3.87	12.66	11.63	0.11	3.41	6.73	2.48	1.63	0.93	97.68	0.51		
**KI76 147 Si-rich glass**	44.8	1090	54.68	3.93	12.48	11.83	0.13	3.61	6.83	3.08	1.64	0.87	99.09	0.54		
**KI76 147 Si-rich glass**	44.8	1090	55.02	3.90	12.57	11.73	0.12	3.65	7.04	2.49	1.67	0.93	99.11	0.53		
**KI76 147 Si-rich glass**	44.8	1090	54.69	3.87	12.51	11.72	0.19	3.53	6.99	2.67	1.61	0.92	98.69	0.53		
**KI76 147 Si-rich glass**	44.8	1090	54.19	3.92	12.52	11.60	0.28	3.60	6.98	1.96	1.60	0.89	97.54	0.51		
**KI76 147 Si-rich glass**	44.8	1090	54.78	3.98	12.74	12.26	0.28	3.34	7.02	2.41	1.66	0.92	99.39	0.52		
**KI76 147 Si-rich glass**	44.8	1090	54.43	4.09	12.47	12.03	0.25	3.67	7.04	3.27	1.53	0.92	99.69	0.56		
**KI76 147 Si-rich glass**	44.8	1090	53.85	4.05	12.43	11.62	0.19	3.71	6.91	3.19	1.59	0.93	98.47	0.55		
**KI76 147 Si-rich glass**	44.8	1090	53.89	4.11	12.54	11.99	0.15	3.51	7.00	3.19	1.55	0.93	98.87	0.55		
**KI76 147 Si-rich glass**	44.8	1090	53.69	4.09	12.65	11.96	0.15	3.58	6.90	3.32	1.60	0.94	98.87	0.55		
**KI76 147 Si-rich glass**	44.8	1090	53.62	4.05	12.72	11.93	0.22	3.46	6.90	2.30	1.56	0.88	97.64	0.52		
**KI76 147 Si-rich glass**	44.8	1090	54.53	4.06	12.24	11.83	0.23	3.50	7.02	3.16	1.51	0.91	98.99	0.55		
**KI76 147 Si-rich glass**	44.8	1090	54.19	4.01	12.28	12.13	0.18	3.61	7.09	2.55	1.63	0.91	98.59	0.55		
**KI76 147 Si-rich glass**	44.8	1090	53.78	3.94	12.70	11.80	0.23	3.55	6.94	3.09	1.59	0.89	98.51	0.54		
**KI76 147 Si-rich glass**	44.8	1090	53.73	4.02	12.47	12.06	0.19	3.69	6.96	3.02	1.61	0.91	98.66	0.56		
**KI76 147 Si-rich glass**	44.8	1090	53.87	4.07	12.78	11.89	0.23	3.67	6.67	3.17	1.65	0.90	98.90	0.54		
**KI76 147 Si-rich glass**	44.8	1090	53.70	3.99	12.47	11.60	0.11	3.69	7.07	2.42	1.62	0.92	97.60	0.53		
**KI76 147 Si-rich glass**	44.8	1090	54.07	3.99	12.50	11.86	0.25	3.61	6.88	3.08	1.63	0.90	98.77	0.55		
**KI76 147 Si-rich glass**	44.8	1090	53.73	4.03	12.50	12.34	0.21	3.68	6.82	2.76	1.60	0.92	98.58	0.55		
**KI76 147 Si-rich glass**	44.8	1090	53.99	3.99	12.35	11.51	0.25	3.75	6.83	2.98	1.57	0.91	98.13	0.55		
**KI76 147 Si-rich glass**	44.8	1090	53.78	3.96	12.74	12.20	0.24	3.74	7.11	3.20	1.60	0.91	99.48	0.57		
Averages		*1090*	*54.22 (0.5)*	*3.99 (0.1)*	*12.55 (0.2)*	*11.88*	*0.19 (0.1)*	*3.62 (0.1)*	*6.94 (0.1)*	*2.87 (0.4)*	*1.60*	*0.91*	*-*	*0.54*	*2.61*	

						(0.3)					(0.01)	(0.01)				
**KI76 145 Average major mineral phases**																
KI76 145 Plagioclase (8)	44.2	*1073*	*51.67 (1.8)*	*0.14 (0.02)*	*28.09 (1.2)*	*0.89 (0.06)*	-	*0.09 (0.01)*	*12.26 (1.3)*	*4.17 (0.7)*	*0.23 (0.07)*	-	-	-	-	*73*
KI76 145 Olivine (8)	44.2	*1073*	*36.39 (0.7)*	*0.19 (0.06)*	-	*28.82 (3.4)*	*0.43 (0.04)*	*33.31 (2.6)*	*0.17 (0.02)*	-	-	-	-	-	-	*47*
KI76 145 Pyroxene (8)	44.2	*1073*	*50.52 (0.5)*	*9.73 (1.1)*	*2.21 (0.3)*	*7.98 (0.9)*	-	*16.11 (0.2)*	*12.32 (0.4)*	*0.11 (0.01)*	-	-	-	-	-	*61*
**KI76 143 Si-rich glass**	43.7	1054	56.81	2.23	12.89	10.39	-	2.17	5.17	2.42	2.32	1.68	96.09	0.38		
**KI76 143 Si-rich glass**	43.7	1054	56.10	2.13	12.70	10.59	-	2.20	5.27	3.05	2.17	1.47	95.86	0.41		
**KI76 143 Si-rich glass**	43.7	1054	55.07	2.37	12.77	11.19	-	2.28	5.44	2.98	2.09	1.56	95.97	0.44		
**KI76 143 Si-rich glass**	43.7	1054	55.92	2.23	12.48	11.01	-	2.33	5.42	2.83	2.23	1.65	96.28	0.43		
**KI76 143 Si-rich glass**	43.7	1054	56.95	2.06	13.73	9.82	-	2.26	5.45	2.34	2.16	1.67	96.54	0.36		
**KI76 143 Si-rich glass**	43.7	1054	57.18	2.02	12.58	9.60	-	2.24	4.71	2.93	2.32	1.37	95.14	0.37		
**KI76 143 Si-rich glass**	43.7	1054	56.50	2.13	13.31	9.97	-	2.33	4.98	3.00	2.48	1.65	96.46	0.39		
**KI76 143 Si-rich glass**	43.7	1054	56.67	2.11	13.09	10.02	-	2.33	5.07	3.11	2.44	1.66	96.67	0.40		
**KI76 143 Si-rich glass**	43.7	1054	55.43	2.14	12.84	9.84	-	2.23	5.06	2.88	2.46	1.59	94.63	0.39		
Averages		*1054*	*56.29 (0.7)*	*2.16 (0.1)*	*12.93 (0.4)*	*10.27 (0.5)*	-	*2.26 (0.1)*	*5.17 (0.2)*	*2.84 (0.3)*	*2.30 (0.1)*	*1.59 (0.1)*	-	*0.40*	*2.54*	
**KI76 143 Average major mineral phases**																
KI76 143 Plagioclase (8)	43.7	*1054*	*50.19 (2.2)*	*0.10 (0.02)*	*29.57 (1.6)*	*0.89 (0.06)*	-	*0.09 (0.01)*	*13.96 (1.9)*	*3.34 (0.9)*	*0.19 (0.1)*	-	-	-	-	*79*
KI76 143 Olivine (8)	43.7	*1054*	*35.54 (0.5)*	*0.37 (0.08)*	-	*34.49 (1.8)*	*0.46 (0.01)*	*28.73 (1.3)*	*0.19 (0.01)*	-	-	-	-	-	-	*39*
KI76 143 Pyroxene (8)	43.7	*1054*	*51.19 (0.6)*	*8.65 (2.3)*	*1.78 (0.6)*	*10.41 (3.7)*	-	*17.06 (2.5)*	*10.55 (3.7)*	*0.10 (0.01)*	-	-	-	-	-	*56*
**KI76 140 Si-rich glass**	42.8	1017	65.52	1.13	13.88	6.21	-	0.75	2.46	2.25	2.80	0.37	95.47	0.12		
**KI76 140 Si-rich glass**	42.8	1017	63.94	1.29	13.94	5.95	-	0.85	2.60	3.20	3.34	0.32	95.54	0.15		
**KI76 140 Si-rich glass**	42.8	1017	63.95	0.92	14.09	5.23	-	0.78	2.30	3.34	3.57	0.35	94.63	0.13		
**KI76 140 Si-rich glass**	42.8	1017	65.15	1.16	13.08	6.43	-	0.74	2.63	2.77	3.10	0.28	95.46	0.16		
**KI76 140 Si-rich glass**	42.8	1017	55.52	1.47	16.01	5.93	-	1.27	6.38	3.26	1.99	3.53	95.48	0.26		
**KI76 140 Si-rich glass**	42.8	1017	64.82	1.17	14.10	4.30	-	0.81	1.83	2.93	4.05	0.34	94.42	0.10		
**KI76 140 Si-rich glass**	42.8	1017	62.26	1.61	13.88	5.64	-	1.00	2.37	3.27	3.82	0.85	94.78	0.16		
**KI76 140 Si-rich glass**	42.8	1017	66.29	1.21	14.12	4.10	-	0.77	1.68	3.34	4.10	0.27	95.93	0.10		
Averages		*1017*	*63.43 (3.2)*	*1.25 (0.2)*	*14.14 (0.8)*	*5.47 (0.8)*	-	*0.87 (0.2)*	*2.78 (1.4)*	*3.05 (0.4)*	*3.35 (0.7)*	*0.79 (1.1)*	-	*0.15*	*2.45*	
**KI76 140 Fe-rich glass**	42.8	1017	40.86	7.51	6.38	26.70	-	7.30	7.88	1.41	0.58	1.03	99.64	1.46	*3.06*	
**KI76 140 Average major mineral phases**																
KI76 140 Plagioclase (8)	42.8	*1017*	*52.31 (3.1)*	*0.12 (0.03)*	*28.07 (1.87)*	*0.86 (0.07)*	-	-	*12.08 (2.29)*	*4.34 (1.1)*	*0.31 (0.18)*	-	-	-	-	*71*
KI76 140 Olivine (8)	42.8	*1017*	*36.28 (0.6)*	*0.30*	-	*31.61*	*0.39*	*31.54*	*0.17 (0.01)*	-	-	-	-	-	-	*44*

				(0.09)		*(1.9)*	*(0.01)*	*(1.5)*								
KI76 140 Pyroxene (8)	42.8	*1017*	*51.36 (0.3)*	*9.35 (0.8)*	*2.17 (0.3)*	*7.73 (0.9)*	-	*16.34 (0.1)*	*12.64 (0.4)*	*0.10 (0.01)*	-	-	-	-	-	*62*
**KI81 249.7 Average major mineral phases**																
KI81 249.7 Plagioclase (8)	76.1	-	*48.72 (0.7)*	*0.14 (0.1)*	*30.72 (0.8)*	*0.93 (0.3)*	-	*0.22 (0.19)*	*15.15 (0.5)*	*2.57 (0.3)*	*0.12 (0.02)*	-	-	-	-	*84*
KI81 249.7 Olivine (8)	76.1	-	*38.66 (0.3)*	*0.02 (0.01)*	-	*18.60 (0.1)*	-	*41.46 (0.1)*	*0.20 (0.02)*	-	-	-	-	-	-	*63*
**KI81 219.8 Average major mineral phases**																
KI81 219.8 Plagioclase (8)	66.9	-	*49.40 (1.0)*	*0.12 (0.01)*	*30.07 (0.5)*	*0.71 (0.04)*	-	*0.17 (0.01)*	*14.79 (0.6)*	*2.87 (0.4)*	*0.12 (0.02)*	-	-	-	-	*83*
KI81 219.8 Olivine (8)	66.9	-	*38.47 (0.3)*	*0.02 (0.01)*	-	*18.75 (0.1)*	-	*41.10 (0.2)*	*0.23 (0.02)*	-	-	-	-	-	-	*63*
**KI81 205.4 Average major mineral phases**																
KI81 205.4 Plagioclase (8)	62.6	-	*51.17 (0.3)*	*0.14 (0.01)*	*28.66 (0.1)*	*0.63 (0.1)*	-	*0.18 (0.01)*	*13.53 (0.1)*	*3.63 (0.1)*	*0.17 (0.01)*	-	-	-	-	*77*
KI81 205.4 Olivine (8)	62.6	-	*38.42 (0.4)*	*0.02 (0.01)*	-	*18.84 (0.2)*	-	*41.33 (0.2)*	*0.23 (0.01)*	-	-	-	-	-	-	*63*
**KI81 192.8 Average major mineral phases**																
KI81 192.8 Plagioclase (8)	58.8	-	*49.70 (1.9)*	*0.09 (0.02)*	*29.76 (1.1)*	*0.86 (0.1)*	-	*0.15 (0.1)*	*14.13 (1.3)*	*3.16 (0.7)*	*0.14 (0.03)*	-	-	-	-	*80*
KI81 192.8 Olivine (8)	58.8	-	*38.91 (0.3)*	*0.02 (0.01)*	-	*20.25 (0.3)*	-	*40.3 (0.4)*	*0.20 (0.01)*	-	-	-	-	-	-	*61*
KI81 192.8 Pyroxene (8)	58.8	-	*51.41 (0.4)*	*0.81 (0.1)*	*4.78 (0.5)*	*6.84 (0.6)*	-	*16.46 (0.1)*	*15.01 (0.1)*	*0.2 (0.01)*	-	-	-	-	-	*65*
**SRP 299 Si-rich glass**																
**SRP 299**	91.3	1096	49.09	4.08	11.73	16.19	0.42	3.53	8.87	2.29	1.71	1.72	99.62	0.76		
**SRP 299**	91.3	1096	48.88	4.06	11.29	16.24	0.31	3.47	8.91	2.60	1.70	1.76	99.22	0.79		
**SRP 299**	91.3	1096	48.47	4.10	11.74	15.60	0.27	3.36	9.04	2.63	1.74	1.77	98.72	0.76		
**SRP 299**	91.3	1096	49.24	3.96	11.95	16.25	0.48	3.59	8.97	2.40	1.74	1.76	100.34	0.77		
**SRP 299**	91.3	1096	48.87	4.09	11.47	16.17	0.32	3.53	8.72	2.51	1.72	1.75	99.17	0.77		
**SRP 299**	91.3	1096	48.71	4.03	11.52	15.91	0.28	3.63	8.75	2.47	1.73	1.73	98.79	0.77		
**SRP 299**	91.3	1096	48.99	4.02	11.36	15.88	0.32	3.70	9.00	2.51	1.74	1.75	99.27	0.79		
**SRP 299**	91.3	1096	48.59	3.96	11.65	16.62	0.28	3.14	8.80	2.52	1.75	1.77	99.10	0.77		
**SRP 299**	91.3	1096	48.39	4.06	11.60	16.18	0.35	3.52	8.78	2.39	1.74	1.74	98.75	0.78		
**SRP 299**	91.3	1096	47.54	3.93	11.47	15.32	0.35	3.65	8.55	2.51	1.72	1.77	96.81	0.77		
**SRP 299**	91.3	1096	47.89	3.96	11.47	16.38	0.32	3.83	8.78	2.51	1.76	1.72	98.62	0.81		
**SRP 299**	91.3	1096	48.20	3.88	11.42	16.34	0.35	3.67	8.68	2.09	1.69	1.75	98.07	0.78		
**SRP 299**	91.3	1096	48.72	3.98	11.38	16.22	0.25	3.61	8.91	2.57	1.86	1.74	99.23	0.79		
**SRP 299**	91.3	1096	49.03	3.92	11.47	16.46		3.75	8.89	2.52	1.89	1.76	100.10	0.80		
**SRP 299**	91.3	1096	49.04	3.94	11.02	16.20	0.18	3.91	8.73	2.54	1.85	1.75	99.16	0.81		
**SRP 299**	91.3	1096	48.34	3.98	11.20	16.24	0.22	4.05	8.67	2.58	1.84	1.74	98.86	0.81		

**SRP 299**	91.3	1096	48.05	3.90	11.59	16.68	0.40	4.03	8.72	2.29	1.88	1.72	99.26	0.82		
**SRP 299**	91.3	1096	48.05	3.92	11.73	16.39	0.29	3.91	8.78	2.29	1.85	1.70	98.92	0.80		
**SRP 299**	91.3	1096	48.17	3.81	11.37	16.09	0.29	3.92	8.68	2.53	1.95	1.69	98.49	0.81		
**SRP 299**	91.3	1096	48.81	3.90	11.45	16.36	0.40	3.96	8.61	2.18	1.85	1.74	99.25	0.79		
**SRP 299**	91.3	1096	48.41	3.85	11.34	16.34	0.24	4.19	8.69	2.41	1.96	1.75	99.18	0.82		
**SRP 299**	91.3	1096	47.74	3.85	11.05	16.43	0.21	4.07	8.43	2.35	1.87	1.72	97.71	0.82		
**SRP 299**	91.3	1096	48.60	3.86	11.47	16.30	0.36	3.83	8.42	2.46	1.86	1.69	98.85	0.79		
**SRP 299**	91.3	1096	48.50	3.95	11.47	16.73	0.37	3.64	9.00	2.30	1.74	1.70	99.40	0.80		
**SRP 299**	91.3	1096	47.24	3.84	11.00	16.73	0.36	3.77	8.71	2.32	1.83	1.68	97.49	0.83		
**SRP 299**	91.3	1096	48.33	3.91	11.15	15.96	0.35	3.82	8.91	2.22	1.85	1.69	98.17	0.80		
**SRP 299**	91.3	1096	47.24	3.88	11.45	16.67	0.41	3.54	8.61	2.15	1.90	1.71	97.55	0.80		
**SRP 299**	91.3	1096	47.67	3.95	11.26	16.13	0.32	3.82	8.52	2.39	1.88	1.74	97.68	0.80		
**SRP 299**	91.3	1096	47.65	3.95	11.63	16.34	0.47	3.59	8.54	2.46	1.80	1.74	98.17	0.79		
**SRP 299**	91.3	1096	48.58	3.97	11.59	16.50	0.27	3.72	8.63	2.58	1.85	1.75	99.45	0.79		
**SRP 299**	91.3	1096	48.10	3.87	11.34	16.48	0.32	3.75	8.69	2.25	1.85	1.73	98.36	0.80		
**SRP 299**	91.3	1096	47.98	3.95	11.45	15.93	0.40	3.72	8.62	2.25	1.81	1.71	97.82	0.78		
**SRP 299**	91.3	1096	47.72	3.94	11.54	16.64	0.27	3.88	8.43	2.64	1.89	1.72	98.67	0.81		
**SRP 299**	91.3	1096	48.24	3.81	11.29	16.42	0.38	4.12	8.72	2.36	1.82	1.66	98.83	0.82		
**SRP 299**	91.3	1096	47.78	3.86	11.48	16.50	0.44	4.05	8.46	2.40	1.82	1.73	98.51	0.81		
**SRP 299**	91.3	1096	47.96	3.95	11.31	16.46	0.33	3.81	8.44	2.31	1.80	1.66	98.03	0.80		
**SRP 299**	91.3	1096	47.58	3.89	11.38	16.39	0.39	3.85	8.68	2.39	1.77	1.70	98.02	0.81		
**SRP 299**	91.3	1096	48.28	3.84	11.70	15.98	0.28	3.95	8.66	2.45	1.73	1.76	98.64	0.79		
**SRP 299**	91.3	1096	47.81	3.86	11.37	16.75	0.27	4.04	8.65	2.37	1.84	1.69	98.64	0.82		
**SRP 299**	91.3	1096	47.23	3.85	11.64	16.78	0.30	3.96	8.63	2.19	1.78	1.68	98.04	0.81		
**SRP 299**	91.3	1096	48.17	3.82	11.27	16.30	0.37	3.83	8.75	2.44	1.88	1.74	98.57	0.81		
**SRP 299**	91.3	1096	48.05	3.90	11.45	16.12	0.28	3.84	8.66	2.21	1.76	1.80	98.07	0.79		
**SRP 299**	91.3	1096	48.28	3.85	11.41	16.14	0.28	3.96	8.64	2.09	1.82	1.72	98.18	0.79		
**SRP 299**	91.3	1096	48.58	3.92	11.64	16.36	0.41	3.90	8.62	2.22	1.84	1.70	99.20	0.79		
**SRP 299**	91.3	1096	49.04	3.93	11.12	16.84	0.32	3.60	8.07	2.43	1.86	1.75	98.96	0.78		
**SRP 299**	91.3	1096	48.53	3.89	10.78	17.00	0.39	3.93	8.29	2.30	1.85	1.69	98.64	0.83		
**SRP 299**	91.3	1096	48.08	3.89	11.53	16.99	0.29	4.15	8.49	2.31	1.95	1.73	99.43	0.82		
**SRP 299**	91.3	1096	47.35	3.89	11.38	16.44	0.40	3.99	8.33	2.39	1.98	1.75	97.89	0.82		
**SRP 299**	91.3	1096	47.96	3.90	11.52	16.78	0.46	3.71	8.51	2.23	1.86	1.73	98.66	0.80		
**SRP 299**	91.3	1096	48.32	3.91	11.61	16.47	0.39	4.01	8.57	2.32	1.88	1.77	99.24	0.80		
**SRP 299**	91.3	1096	47.54	3.86	11.69	16.21	0.31	3.89	8.50	2.35	1.91	1.67	97.92	0.79		
**SRP 299**	91.3	1096	48.14	3.89	11.40	16.93	0.49	4.03	8.55	2.48	1.84	1.74	99.48	0.83		
**SRP 299**	91.3	1096	47.25	3.89	11.50	15.85	0.23	3.94	8.62	2.41	1.84	1.71	97.24	0.80		
**SRP 299**	91.3	1096	47.57	3.80	11.65	16.30	0.28	3.79	8.64	2.44	1.82	1.66	97.94	0.80		
**SRP 299**	91.3	1096	48.58	3.92	11.57	16.94	0.43	4.08	8.80	2.12	1.77	1.72	99.92	0.81		
**SRP 299**	91.3	1096	48.02	3.90	11.63	16.26	0.24	3.81	8.61	2.63	1.79	1.75	98.63	0.79		
**SRP 299**	91.3	1096	47.75	3.94	11.33	16.96	0.42	3.83	8.48	2.45	1.73	1.73	98.61	0.82		
**SRP 299**	91.3	1096	47.97	3.89	11.60	16.41	0.37	3.78	8.76	2.37	1.83	1.74	98.71	0.80		
**SRP 299**	91.3	1096	47.67	3.96	11.38	16.33	0.30	3.97	8.55	2.22	1.83	1.70	97.90	0.80		
**SRP 299**	91.3	1096	48.28	4.02	11.35	16.44	0.45	3.99	8.57	2.13	1.80	1.71	98.74	0.80		

**SRP 299**	91.3	1096	48.63	3.88	11.71	16.42	0.36	3.91	8.55	2.09	1.82	1.69	99.06	0.78		
**SRP 299**	91.3	1096	48.24	3.86	11.41	16.98	0.38	3.79	8.61	2.42	1.82	1.74	99.26	0.82		
**SRP 299**	91.3	1096	47.59	3.94	10.95	16.72	0.43	3.98	8.62	2.26	1.89	1.71	98.08	0.84		
**SRP 299**	91.3	1096	47.92	3.97	11.53	16.46	0.37	3.92	8.42	2.20	1.74	1.71	98.24	0.79		
**SRP 299**	91.3	1096	47.39	3.87	11.37	16.33	0.45	3.97	8.46	2.43	1.72	1.74	97.72	0.81		
**SRP 299**	91.3	1096	47.79	3.94	11.46	16.87	0.30	3.93	8.66	2.42	1.73	1.72	98.82	0.82		
**SRP 299**	91.3	1096	47.26	3.88	11.34	16.53	0.41	3.88	8.73	2.40	1.68	1.70	97.81	0.82		
**SRP 299**	91.3	1096	48.84	3.86	11.40	16.79	0.32	3.83	8.63	2.47	1.68	1.71	99.51	0.80		
**SRP 299**	91.3	1096	47.96	3.92	11.51	16.62	0.30	4.06	8.76	2.16	1.65	1.70	98.64	0.81		
**SRP 299**	91.3	1096	48.14	3.86	11.63	16.52	0.25	4.03	8.70	2.49	1.71	1.69	99.01	0.81		
**SRP 299**	91.3	1096	48.07	3.86	11.57	16.97	0.43	3.93	8.64	2.03	1.69	1.69	98.88	0.81		
**SRP 299**	91.3	1096	48.04	3.88	11.38	16.55	0.27	3.96	8.64	2.28	1.68	1.74	98.39	0.81		
**SRP 299**	91.3	1096	48.60	3.86	11.70	16.34	0.31	3.96	8.50	2.24	1.74	1.72	98.97	0.78		
**SRP 299**	91.3	1096	48.50	3.90	11.46	16.29	0.42	4.04	8.63	2.50	1.73	1.67	99.13	0.81		
**SRP 299**	91.3	1096	47.74	3.84	11.69	16.85	0.28	3.70	8.63	2.05	1.59	1.71	98.08	0.79		
**SRP 299**	91.3	1096	48.27	3.92	11.68	16.53	0.45	4.20	8.40	2.40	1.67	1.71	99.22	0.80		
**SRP 299**	91.3	1096	47.09	3.90	11.24	16.35	0.36	3.74	8.60	2.57	1.76	1.67	97.30	0.82		
**SRP 299**	91.3	1096	47.73	3.95	11.71	16.47	0.50	3.76	8.74	2.16	1.70	1.74	98.46	0.79		
**SRP 299**	91.3	1096	47.73	3.90	11.63	16.62	0.37	3.99	8.63	2.51	1.72	1.69	98.78	0.81		
**SRP 299**	91.3	1096	48.29	3.83	11.44	16.37	0.38	3.82	8.66	2.36	1.85	1.69	98.68	0.80		
**SRP 299**	91.3	1096	47.52	3.80	11.43	16.43	0.34	3.80	8.32	2.05	1.68	1.67	97.02	0.79		
**SRP 299**	91.3	1096	46.81	3.81	11.68	16.38	0.41	3.80	8.76	2.15	1.70	1.72	97.21	0.80		
**SRP 299**	91.3	1096	48.53	3.78	11.31	16.57	0.27	3.92	8.42	2.28	1.75	1.70	98.53	0.80		
**SRP 299**	91.3	1096	47.60	3.82	11.70	16.73	0.40	3.77	8.67	2.28	1.63	1.70	98.29	0.80		
**SRP 299**	91.3	1096	48.10	3.90	11.50	15.36	0.32	3.78	8.86	2.33	1.67	1.76	97.58	0.77		
**SRP 299**	91.3	1096	47.94	3.97	11.47	16.50	0.32	3.88	8.75	2.33	1.74	1.75	98.65	0.81		
**SRP 299**	91.3	1096	48.43	3.94	11.48	16.78	0.29	4.00	8.78	2.40	1.72	1.68	99.50	0.81		
**SRP 299**	91.3	1096	47.62	3.84	11.37	16.21	0.50	4.07	8.59	2.31	1.73	1.70	97.94	0.81		
**SRP 299**	91.3	1096	48.16	3.83	10.98	16.21	0.30	4.00	8.72	2.65	1.75	1.72	98.31	0.83		
**SRP 299**	91.3	1096	47.30	3.92	11.14	16.81	0.39	3.75	8.65	2.38	1.73	1.74	97.83	0.83		
**SRP 299**	91.3	1096	48.31	3.84	11.59	15.91	0.32	4.05	8.53	2.17	1.79	1.72	98.24	0.78		
**SRP 299**	91.3	1096	48.30	3.90	11.26	16.44	0.31	3.87	8.55	1.99	1.75	1.69	98.06	0.79		
**SRP 299**	91.3	1096	48.06	3.86	11.10	16.46	0.29	3.87	8.53	2.34	1.79	1.74	98.04	0.81		
**SRP 299**	91.3	1096	48.13	3.88	11.29	16.00	0.28	3.87	8.55	2.50	1.75	1.76	98.03	0.80		
**SRP 299**	91.3	1096	47.71	3.82	11.70	16.45	0.36	3.78	8.70	2.20	1.70	1.71	98.15	0.79		
**SRP 299**	91.3	1096	48.24	3.88	11.63	16.29	0.35	3.95	8.52	2.37	1.71	1.69	98.61	0.79		
**SRP 299**	91.3	1096	48.97	3.88	11.45	16.33	0.23	3.87	8.34	2.43	1.76	1.73	99.00	0.78		
**SRP 299**	91.3	1096	47.51	3.89	11.87	16.61	0.33	4.06	8.33	2.44	1.71	1.70	98.46	0.80		
**SRP 299**	91.3	1096	47.57	3.85	11.52	16.47	0.33	3.91	8.58	2.23	1.75	1.68	97.88	0.80		
**SRP 299**	91.3	1096	47.74	3.88	11.29	16.58	0.33	3.88	8.43	2.45	1.72	1.65	97.96	0.81		
**SRP 299**	91.3	1096	47.22	3.76	11.38	16.54	0.40	4.05	8.50	2.37	1.76	1.71	97.67	0.82		
Averages		*1096*	*48.08*	*3.90*	*11.45*	*16.41*	*0.34*	*3.85*	*8.63*	*2.35*	*1.78*	*1.72*	*-*	*0.80*	*2.76*	
	**SRP 299 Fe-rich glass**															
**SRP 299**	91.3	1096	39.19	6.61	4.24	25.63	0.51	6.17	11.30	1.28	0.43	2.89	98.24	1.68		

**SRP 299**	91.3	1096	46.50	4.64	8.69	19.29	0.24	4.44	8.88	2.15	1.35	1.87	98.05	1.01		
**SRP 299**	91.3	1096	40.83	6.56	4.64	25.51	0.46	5.97	10.90	1.32	0.60	3.02	99.81	1.58		
Averages			*42.18*	*5.94*	*5.86*	*23.47*	*0.40*	*5.53*	*10.36*	*1.58*	*0.79*	*2.59*	*-*	*1.42*	*2.95*	
**SRP 298 Si-rich glass**																
**SRP 298**	90.8	1093	48.91	3.91	11.29	16.43	0.32	3.90	8.66	2.44	1.74	1.75	99.33	0.80		
**SRP 298**	90.8	1093	48.54	3.88	11.58	16.68	0.37	3.70	9.06	2.54	1.78	1.74	99.87	0.81		
**SRP 298**	90.8	1093	48.51	3.93	11.39	16.01	0.46	3.64	8.54	2.41	1.82	1.78	98.48	0.78		
**SRP 298**	90.8	1093	47.92	3.84	11.55	16.45	0.30	3.77	8.73	2.40	1.80	1.70	98.45	0.80		
**SRP 298**	90.8	1093	48.30	3.91	11.35	16.07	0.25	3.80	8.82	2.49	1.79	1.69	98.47	0.80		
**SRP 298**	90.8	1093	48.03	3.86	11.38	16.84	0.30	3.90	8.84	2.32	1.67	1.75	98.87	0.82		
**SRP 298**	90.8	1093	48.22	3.89	11.87	16.32	0.36	3.64	8.73	2.58	1.85	1.72	99.20	0.79		
**SRP 298**	90.8	1093	47.73	3.89	11.69	16.86	0.32	3.83	8.73	2.38	1.86	1.74	99.04	0.81		
**SRP 298**	90.8	1093	48.10	3.88	11.69	16.60	0.23	3.85	8.90	2.52	1.82	1.72	99.29	0.81		
**SRP 298**	90.8	1093	49.04	3.90	11.47	16.19	0.34	3.79	8.81	2.39	1.86	1.73	99.53	0.79		
**SRP 298**	90.8	1093	48.31	3.94	10.93	16.36	0.36	3.82	8.59	2.37	1.85	1.75	98.28	0.81		
**SRP 298**	90.8	1093	48.70	3.96	11.11	16.76	0.39	3.73	8.74	2.19	1.87	1.72	99.17	0.81		
**SRP 298**	90.8	1093	48.12	3.95	11.03	16.57	0.51	3.78	8.39	2.47	1.91	1.76	98.49	0.82		
**SRP 298**	90.8	1093	48.46	3.89	11.54	16.47	0.23	3.74	8.54	2.53	1.74	1.69	98.82	0.79		
**SRP 298**	90.8	1093	48.05	3.90	11.36	16.40	0.31	3.73	8.75	2.66	1.87	1.76	98.79	0.81		
**SRP 298**	90.8	1093	48.80	3.91	11.65	16.47	0.31	3.64	8.64	2.45	1.90	1.72	99.51	0.78		
**SRP 298**	90.8	1093	47.96	3.94	11.60	16.14	0.36	3.80	8.69	2.54	1.95	1.72	98.69	0.80		
**SRP 298**	90.8	1093	47.84	3.78	11.41	16.40	0.34	3.69	8.72	2.36	1.84	1.75	98.13	0.80		
**SRP 298**	90.8	1093	47.12	3.94	11.57	16.46	0.40	4.00	8.52	2.51	1.86	1.72	98.10	0.82		
**SRP 298**	90.8	1093	48.06	3.91	11.08	16.21	0.34	3.68	8.54	2.61	1.91	1.67	98.01	0.81		
**SRP 298**	90.8	1093	48.40	3.94	11.41	16.88	0.31	3.85	8.69	2.60	1.94	1.79	99.83	0.82		
**SRP 298**	90.8	1093	48.81	4.01	11.39	16.42	0.37	3.84	8.69	2.15	1.90	1.74	99.31	0.79		
**SRP 298**	90.8	1093	48.34	3.84	11.48	16.36	0.39	3.69	8.64	2.35	1.82	1.74	98.64	0.79		
**SRP 298**	90.8	1093	48.45	3.90	11.74	16.18	0.27	3.92	8.83	2.49	1.91	1.72	99.41	0.80		
**SRP 298**	90.8	1093	47.99	3.84	11.71	15.77	0.31	4.05	8.58	2.42	1.93	1.73	98.32	0.79		
**SRP 298**	90.8	1093	48.35	3.89	11.47	16.22	0.20	4.02	8.57	2.42	1.93	1.74	98.81	0.80		
**SRP 298**	90.8	1093	47.83	3.95	11.39	16.74	0.33	3.99	8.45	2.73	1.86	1.69	98.97	0.83		
**SRP 298**	90.8	1093	48.70	3.86	11.46	16.27	0.29	3.79	8.69	2.57	1.90	1.71	99.22	0.80		
**SRP 298**	90.8	1093	47.29	3.80	11.37	16.66	0.23	3.75	8.45	2.59	1.88	1.77	97.79	0.82		
**SRP 298**	90.8	1093	47.86	3.85	11.68	16.81	0.43	3.90	8.51	2.67	1.86	1.75	99.33	0.82		
**SRP 298**	90.8	1093	48.39	3.86	11.30	16.07	0.37	3.89	8.39	2.42	1.86	1.73	98.26	0.79		
**SRP 298**	90.8	1093	47.83	3.85	11.58	16.27	0.27	3.82	8.67	2.47	1.81	1.72	98.29	0.80		
**SRP 298**	90.8	1093	47.75	3.89	11.57	16.19	0.31	3.86	8.55	2.46	1.93	1.70	98.21	0.80		
**SRP 298**	90.8	1093	48.10	3.90	11.53	16.35	0.35	3.97	8.64	2.49	1.94	1.72	98.99	0.81		
**SRP 298**	90.8	1093	47.86	3.93	11.56	16.58	0.29	3.90	8.69	2.48	1.86	1.72	98.88	0.81		
**SRP 298**	90.8	1093	49.35	3.94	11.79	16.53	0.39	3.71	8.65	2.67	1.94	1.75	100.72	0.79		
**SRP 298**	90.8	1093	48.53	3.94	11.49	16.69	0.26	3.89	8.83	2.11	1.88	1.75	99.36	0.80		
**SRP 298**	90.8	1093	47.64	3.98	11.65	16.11	0.34	3.84	8.34	2.48	1.96	1.74	98.07	0.79		
**SRP 298**	90.8	1093	48.52	4.00	11.39	16.69	0.38	3.57	8.59	2.39	1.90	1.74	99.17	0.79		

**SRP 298**	90.8	1093	48.79	4.00	11.40	16.39	0.24	3.82	8.47	2.48	1.87	1.75	99.20	0.79		
**SRP 298**	90.8	1093	48.26	4.03	10.94	16.83	0.43	3.78	8.40	2.30	1.92	1.78	98.66	0.82		
**SRP 298**	90.8	1093	47.81	3.90	11.26	16.62	0.47	3.88	8.81	2.35	1.76	1.73	98.59	0.83		
**SRP 298**	90.8	1093	47.60	3.85	11.57	16.12	0.35	3.57	8.74	2.41	1.78	1.71	97.69	0.79		
**SRP 298**	90.8	1093	48.39	3.93	11.52	16.63	0.30	3.76	8.53	2.39	1.80	1.71	98.97	0.79		
**SRP 298**	90.8	1093	48.18	3.88	11.63	16.93	0.22	3.67	8.55	2.56	1.75	1.78	99.14	0.80		
**SRP 298**	90.8	1093	48.22	3.93	11.43	15.96	0.35	3.88	8.74	2.30	1.74	1.75	98.32	0.79		
**SRP 298**	90.8	1093	48.24	3.94	11.53	16.83	0.34	3.78	8.65	2.54	1.87	1.73	99.45	0.81		
**SRP 298**	90.8	1093	47.73	3.89	11.13	16.89	0.32	3.60	8.58	2.26	1.71	1.73	97.84	0.81		
**SRP 298**	90.8	1093	47.97	4.05	11.43	16.46	0.33	3.98	8.69	2.59	1.79	1.70	98.99	0.82		
**SRP 298**	90.8	1093	47.81	4.07	10.86	16.09	0.15	3.79	9.15	3.06	1.14	1.82	97.94	0.83		
**SRP 298**	90.8	1093	49.08	3.91	11.40	16.39	0.46	3.70	8.81	2.50	1.82	1.73	99.80	0.80		
**SRP 298**	90.8	1093	47.79	3.87	11.26	16.45	0.36	3.75	8.56	2.43	1.78	1.71	97.96	0.81		
**SRP 298**	90.8	1093	49.24	3.91	11.31	16.53	0.37	3.92	8.92	2.41	1.71	1.72	100.04	0.81		
**SRP 298**	90.8	1093	48.57	3.84	11.05	16.77	0.27	3.72	8.51	2.53	1.77	1.74	98.79	0.81		
**SRP 298**	90.8	1093	47.34	4.00	11.62	16.55	0.34	3.93	8.73	2.37	1.74	1.74	98.35	0.81		
**SRP 298**	90.8	1093	47.76	3.98	11.60	16.05	0.35	3.65	8.85	2.24	1.77	1.71	97.96	0.78		
**SRP 298**	90.8	1093	48.27	3.91	11.70	16.51	0.18	3.77	8.62	2.32	1.83	1.72	98.83	0.78		
**SRP 298**	90.8	1093	47.96	3.92	11.38	16.36	0.39	3.50	8.82	2.33	1.80	1.71	98.17	0.79		
**SRP 298**	90.8	1093	48.63	4.01	11.45	16.44	0.41	3.62	8.57	2.50	1.82	1.71	99.16	0.79		
**SRP 298**	90.8	1093	48.17	3.96	11.35	17.22	0.35	3.69	8.73	2.61	1.87	1.75	99.70	0.83		
**SRP 298**	90.8	1093	48.58	3.97	11.42	16.30	0.35	3.70	8.83	2.27	1.82	1.73	98.95	0.79		
**SRP 298**	90.8	1093	47.91	3.95	11.42	16.77	0.34	3.62	8.65	2.33	1.83	1.72	98.54	0.80		
**SRP 298**	90.8	1093	48.36	4.03	11.44	16.23	0.29	3.62	8.54	2.11	1.85	1.75	98.21	0.77		
**SRP 298**	90.8	1093	47.84	3.95	11.26	16.08	0.40	3.69	8.56	2.25	1.94	1.75	97.72	0.79		
**SRP 298**	90.8	1093	48.17	3.92	11.36	16.80	0.13	4.11	8.63	2.41	1.84	1.75	99.11	0.82		
**SRP 298**	90.8	1093	48.39	3.94	11.33	16.63	0.45	4.06	8.64	2.63	1.82	1.75	99.64	0.83		
**SRP 298**	90.8	1093	47.45	3.85	11.46	16.36	0.38	4.00	8.74	2.77	1.84	1.76	98.61	0.83		
**SRP 298**	90.8	1093	49.48	3.95	11.24	16.17	0.45	3.86	8.73	2.34	1.87	1.76	99.83	0.79		
**SRP 298**	90.8	1093	47.36	3.93	11.58	16.50	0.41	3.69	8.49	2.46	1.87	1.76	98.04	0.80		
**SRP 298**	90.8	1093	48.44	3.96	11.58	17.01	0.29	3.91	8.81	2.60	1.86	1.76	100.22	0.82		
**SRP 298**	90.8	1093	49.32	3.89	11.43	16.31	0.30	3.83	8.57	2.59	1.78	1.79	99.81	0.79		
**SRP 298**	90.8	1093	48.34	3.93	11.30	16.31	0.38	3.88	8.55	2.61	1.82	1.76	98.88	0.81		
**SRP 298**	90.8	1093	48.44	3.97	11.45	16.78	0.42	4.07	8.67	2.40	1.88	1.72	99.80	0.82		
**SRP 298**	90.8	1093	48.13	3.90	11.67	16.56	0.27	3.66	8.81	2.39	1.69	1.78	98.87	0.79		
**SRP 298**	90.8	1093	48.61	3.82	11.30	16.52	0.26	3.62	8.82	2.63	1.78	1.74	99.11	0.80		
**SRP 298**	90.8	1093	48.70	3.81	11.71	16.33	0.24	3.72	8.90	2.39	1.75	1.73	99.30	0.78		
**SRP 298**	90.8	1093	48.03	4.01	11.48	16.18	0.27	3.75	8.57	2.56	1.81	1.75	98.43	0.79		
**SRP 298**	90.8	1093	48.13	3.96	11.80	16.89	0.34	3.59	8.86	2.78	1.75	1.72	99.83	0.81		
**SRP 298**	90.8	1093	47.83	3.95	11.08	16.53	0.39	3.92	8.97	2.41	1.76	1.72	98.56	0.84		
**SRP 298**	90.8	1093	48.73	3.99	11.50	16.76	0.36	3.66	8.57	2.26	1.77	1.76	99.36	0.79		
**SRP 298**	90.8	1093	48.00	3.92	11.74	16.16	0.21	3.57	8.66	2.32	1.79	1.75	98.12	0.77		
**SRP 298**	90.8	1093	48.56	4.05	11.44	16.17	0.44	3.59	8.81	2.10	1.73	1.72	98.61	0.78		
**SRP 298**	90.8	1093	48.23	4.04	11.41	16.41	0.38	3.55	8.67	2.49	1.85	1.72	98.75	0.79		

**SRP 298**	90.8	1093	48.16	4.02	11.34	16.65	0.35	3.70	8.88	2.32	1.83	1.72	98.97	0.81		
**SRP 298**	90.8	1093	48.43	4.01	11.22	16.30	0.32	3.57	8.76	2.41	1.86	1.73	98.62	0.79		
**SRP 298**	90.8	1093	48.97	4.00	11.15	16.78	0.27	3.53	8.83	2.13	1.74	1.79	99.19	0.79		
**SRP 298**	90.8	1093	48.13	4.00	11.49	16.23	0.32	3.83	8.94	2.67	1.73	1.72	99.07	0.81		
**SRP 298**	90.8	1093	48.47	4.00	11.47	16.36	0.34	3.71	8.75	2.43	1.72	1.78	99.02	0.79		
**SRP 298**	90.8	1093	48.18	3.94	11.66	16.34	0.49	3.80	8.61	2.40	1.77	1.72	98.91	0.79		
**SRP 298**	90.8	1093	48.10	4.18	11.03	16.22	0.39	3.67	8.72	2.43	1.73	1.73	98.20	0.80		
**SRP 298**	90.8	1093	48.94	4.12	11.32	16.04	0.30	3.52	8.74	2.44	1.75	1.75	98.92	0.77		
**SRP 298**	90.8	1093	48.11	4.22	11.23	16.25	0.40	3.35	8.61	2.22	1.82	1.78	97.97	0.78		
**SRP 298**	90.8	1093	48.15	4.26	11.26	16.16	0.24	3.46	8.77	2.39	1.80	1.80	98.29	0.78		
Averages		*1093*	*48.25*	*3.94*	*11.43*	*16.45*	*0.33*	*3.77*	*8.68*	*2.45*	*1.82*	*1.74*	*-*	*0.80*	*2.75*	
**SRP 298 Fe-rich glass**																
**SRP 298**	90.8	1093	38.84	6.49	4.38	26.18	0.47	6.04	11.08	1.31	0.57	2.97	98.34	1.69		
**SRP 298**	90.8	1093	45.07	5.17	6.41	21.09	0.38	4.55	9.65	1.80	1.39	2.25	97.75	1.19		
**SRP 298**	90.8	1093	42.93	5.12	7.64	21.18	0.56	5.05	9.66	1.84	1.30	2.32	97.60	1.22		
**SRP 298**	90.8	1093	44.17	5.48	7.19	21.41	0.41	5.20	10.02	1.86	1.19	2.49	99.40	1.23		
**SRP 298**	90.8	1093	42.67	5.56	8.13	20.97	0.64	4.54	11.10	2.71	0.54	2.50	99.35	1.23		
**SRP 298**	90.8	1093	45.42	4.86	7.60	20.47	0.38	4.24	9.32	1.92	1.53	2.09	97.83	1.10		
**SRP 298**	90.8	1093	41.73	5.71	6.07	23.30	0.55	5.03	10.51	1.70	0.87	2.65	98.12	1.39		
**SRP 298**	90.8	1093	47.19	4.67	8.30	18.68	0.46	4.04	9.21	2.09	1.70	2.00	98.33	1.00		
**SRP 298**	90.8	1093	46.26	4.91	8.86	18.67	0.42	4.29	9.60	2.27	1.44	2.13	98.86	1.01		
Averages			*43.81*	*5.33*	*7.17*	*21.33*	*0.48*	*4.77*	*10.02*	*1.94*	*1.17*	*2.38*	*-*	*1.23*	*2.89*	
**LAKI 04 Si-rich glass**																
**LAKI 04**	-	1114	49.52	3.93	11.74	15.20	0.38	4.71	9.11	2.96	0.55	0.56	98.66	0.79		
**LAKI 04**	-	1114	49.69	3.96	11.73	16.09	0.18	4.49	9.06	2.79	0.57	0.54	99.12	0.79		
**LAKI 04**	-	1114	49.10	4.04	11.74	16.01	0.30	4.53	8.97	2.97	0.57	0.57	98.80	0.80		
**LAKI 04**	-	1114	49.10	4.04	11.31	15.81	0.17	4.47	8.98	2.74	0.54	0.59	97.75	0.80		
**LAKI 04**	-	1114	48.71	3.96	11.61	16.36	0.31	4.64	9.02	3.02	0.60	0.54	98.78	0.83		
**LAKI 04**	-	1114	49.86	3.90	11.66	16.03	0.26	4.44	9.11	2.77	0.62	0.55	99.21	0.79		
**LAKI 04**	-	1114	49.76	3.92	11.48	16.28	0.28	4.58	9.18	2.89	0.61	0.57	99.53	0.81		
**LAKI 04**	-	1114	49.48	3.92	11.50	15.47	0.36	4.59	9.12	2.86	0.57	0.56	98.44	0.80		
**LAKI 04**	-	1114	49.54	3.99	11.59	15.64	0.28	4.67	8.96	2.71	0.59	0.55	98.52	0.79		
**LAKI 04**	-	1114	49.04	3.90	11.61	16.30	0.20	4.67	9.19	2.72	0.61	0.54	98.79	0.82		
**LAKI 04**	-	1114	49.25	3.90	11.36	15.57	0.21	4.50	9.14	2.77	0.55	0.55	97.80	0.80		
**LAKI 04**	-	1114	49.23	3.97	11.80	15.50	0.23	4.50	9.13	2.88	0.57	0.50	98.31	0.78		
**LAKI 04**	-	1114	49.60	3.98	11.12	15.77	0.27	4.54	9.00	2.81	0.58	0.52	98.21	0.80		
**LAKI 04**	-	1114	49.97	3.95	11.79	16.03	0.21	4.50	8.95	2.95	0.57	0.56	99.48	0.78		
**LAKI 04**	-	1114	49.74	3.77	11.75	16.15	0.23	4.62	9.06	3.03	0.56	0.56	99.47	0.80		
**LAKI 04**	-	1114	49.48	3.85	11.44	15.61	0.29	4.76	9.15	2.72	0.60	0.59	98.49	0.81		
**LAKI 04**	-	1114	50.04	3.94	11.28	16.00	0.33	4.48	9.09	2.78	0.59	0.56	99.09	0.80		
**LAKI 04**	-	1114	49.54	3.85	11.24	15.30	0.36	4.49	9.19	2.23	0.55	0.54	97.28	0.78		
**LAKI 04**	-	1114	49.06	3.93	11.55	15.68	0.14	4.66	9.05	3.01	0.51	0.56	98.15	0.80		
**LAKI 04**	-	1114	49.66	3.96	11.53	16.24	0.23	4.73	9.31	2.73	0.55	0.54	99.49	0.82		

**LAKI 04**	-	1114	49.74	3.96	11.09	16.08	0.27	4.69	9.19	2.70	0.56	0.60	98.90	0.82		
**LAKI 04**	-	1114	48.52	4.01	11.18	16.25	0.23	4.67	8.92	2.31	0.61	0.59	97.28	0.82		
**LAKI 04**	-	1114	49.33	3.98	11.62	16.34	0.19	4.46	8.90	2.96	0.57	0.54	98.90	0.80		
**LAKI 04**	-	1114	50.27	3.87	11.39	15.46	0.16	4.59	8.99	2.82	0.59	0.58	98.73	0.78		
**LAKI 04**	-	1114	49.59	3.90	11.35	15.77	0.26	4.44	9.12	2.82	0.56	0.55	98.36	0.80		
**LAKI 04**	-	1114	49.77	3.89	11.66	15.92	0.21	4.63	8.76	2.79	0.60	0.52	98.75	0.78		
**LAKI 04**	-	1114	49.89	3.92	11.55	16.51	0.19	4.65	9.18	3.00	0.62	0.52	100.02	0.82		
**LAKI 04**	-	1114	49.91	3.88	11.53	16.69	0.17	4.79	9.03	2.62	0.58	0.57	99.77	0.82		
**LAKI 04**	-	1114	49.56	3.88	11.53	15.69	0.24	4.40	9.06	2.80	0.55	0.55	98.23	0.78		
**LAKI 04**	-	1114	49.35	3.90	11.44	15.78	0.25	4.61	9.12	2.70	0.57	0.53	98.26	0.80		
**LAKI 04**	-	1114	49.15	3.97	11.67	15.95	0.23	4.39	9.26	2.23	0.57	0.55	97.97	0.78		
**LAKI 04**	-	1114	49.68	3.97	11.60	15.58	0.19	4.61	9.18	2.68	0.58	0.54	98.60	0.79		
**LAKI 04**	-	1114	49.81	3.97	11.47	16.33	0.32	4.63	9.05	2.76	0.57	0.55	99.47	0.81		
**LAKI 04**	-	1114	49.02	3.92	11.45	15.66	0.38	4.37	9.22	2.86	0.58	0.57	98.02	0.80		
**LAKI 04**	-	1114	49.91	3.92	11.52	15.83	0.31	4.47	9.15	2.91	0.60	0.53	99.15	0.80		
**LAKI 04**	-	1114	48.83	3.88	11.46	15.71	0.31	4.45	9.14	2.61	0.57	0.57	97.54	0.80		
**LAKI 04**	-	1114	49.90	3.84	11.64	16.13	0.23	4.57	8.89	2.61	0.60	0.55	98.96	0.79		
**LAKI 04**	-	1114	48.77	3.97	11.36	15.73	0.27	4.59	9.19	2.84	0.59	0.57	97.89	0.82		
**LAKI 04**	-	1114	49.87	4.04	11.63	15.80	0.19	4.46	9.13	2.94	0.56	0.54	99.18	0.79		
**LAKI 04**	-	1114	49.14	3.96	11.29	16.03	0.22	4.63	9.04	2.70	0.54	0.54	98.10	0.81		
**LAKI 04**	-	1114	49.50	3.90	11.12	16.15	0.25	4.44	9.33	2.88	0.58	0.57	98.72	0.82		
**LAKI 04**	-	1114	49.25	3.95	11.27	15.74	0.40	4.63	9.06	2.82	0.56	0.54	98.22	0.81		
**LAKI 04**	-	1114	49.48	4.03	11.32	16.54	0.35	4.65	9.22	2.85	0.58	0.53	99.55	0.83		
**LAKI 04**	-	1114	49.23	4.03	10.71	16.11	0.24	4.59	9.17	2.61	0.59	0.58	97.85	0.83		
**LAKI 04**	-	1114	50.53	3.99	10.87	16.32	0.23	4.68	9.20	2.78	0.56	0.58	99.75	0.83		
**LAKI 04**	-	1114	48.91	4.07	10.77	16.35	0.30	4.51	8.87	2.80	0.63	0.54	97.74	0.83		
**LAKI 04**	-	1114	49.62	3.95	11.30	16.45	0.30	4.54	9.04	2.72	0.62	0.57	99.11	0.82		
**LAKI 04**	-	1114	48.94	3.85	11.12	16.22	0.31	4.72	8.89	2.72	0.59	0.57	97.93	0.83		
**LAKI 04**	-	1114	49.62	3.94	11.50	16.00	0.34	4.51	8.90	2.78	0.62	0.54	98.74	0.80		
**LAKI 04**	-	1114	50.44	3.85	11.46	15.99	0.27	4.77	9.18	2.79	0.64	0.53	99.93	0.81		
**LAKI 04**	-	1114	49.14	3.91	11.63	16.25	0.25	4.36	9.23	2.95	0.54	0.56	98.82	0.81		
**LAKI 04**	-	1114	49.41	3.97	11.63	15.74	0.27	4.69	9.06	2.77	0.58	0.54	98.66	0.80		
**LAKI 04**	-	1114	49.63	3.90	11.41	15.57	0.22	4.39	9.09	2.50	0.56	0.57	97.83	0.78		
**LAKI 04**	-	1114	49.62	3.84	11.51	15.99	0.28	4.55	9.09	2.88	0.57	0.53	98.86	0.80		
**LAKI 04**	-	1114	49.18	3.92	11.57	15.57	0.25	4.36	8.99	2.85	0.53	0.55	97.78	0.78		
**LAKI 04**	-	1114	48.69	3.90	11.36	15.41	0.28	4.38	9.30	2.78	0.57	0.55	97.22	0.80		
**LAKI 04**	-	1114	50.03	4.00	11.67	15.82	0.31	4.48	9.11	2.69	0.61	0.54	99.26	0.78		
**LAKI 04**	-	1114	49.72	3.94	11.21	15.92	0.25	4.37	9.17	3.01	0.57	0.54	98.69	0.81		
**LAKI 04**	-	1114	49.47	4.03	11.26	15.82	0.17	4.30	9.13	2.80	0.58	0.56	98.14	0.79		
**LAKI 04**	-	1114	49.60	3.98	11.47	16.46	0.25	4.53	9.03	2.94	0.56	0.58	99.39	0.81		
**LAKI 04**	-	1114	48.32	3.96	11.38	16.01	0.28	4.46	9.17	2.77	0.57	0.56	97.47	0.82		
**LAKI 04**	-	1114	48.93	3.90	11.71	16.13	0.38	4.44	9.03	2.75	0.57	0.55	98.40	0.80		
**LAKI 04**	-	1114	48.52	3.82	11.41	15.89	0.25	4.43	8.92	2.53	0.55	0.56	96.88	0.80		
**LAKI 04**	-	1114	48.91	3.91	11.47	15.59	0.29	4.35	8.91	1.98	0.53	0.53	96.47	0.76		

**LAKI 04**	-	1114	49.60	3.95	11.48	16.01	0.34	4.35	9.16	2.77	0.56	0.58	98.79	0.80		
**LAKI 04**	-	1114	49.74	3.95	11.42	16.17	0.33	4.46	9.15	3.02	0.56	0.58	99.38	0.81		
**LAKI 04**	-	1114	49.15	3.94	11.66	15.50	0.26	4.54	9.01	2.73	0.59	0.58	97.96	0.78		
**LAKI 04**	-	1114	49.82	3.93	11.61	16.20	0.32	4.33	8.93	2.77	0.60	0.57	99.08	0.79		
**LAKI 04**	-	1114	49.02	3.95	11.34	15.88	0.26	4.71	9.10	2.96	0.57	0.54	98.32	0.82		
**LAKI 04**	-	1114	49.09	3.92	11.50	15.60	0.28	4.58	8.95	2.93	0.57	0.58	98.00	0.80		
**LAKI 04**	-	1114	49.16	3.92	11.59	15.84	0.21	4.51	9.02	2.79	0.56	0.55	98.16	0.79		
**LAKI 04**	-	1114	49.14	3.99	11.53	15.65	0.35	4.69	9.08	2.78	0.53	0.53	98.26	0.80		
**LAKI 04**	-	1114	49.74	3.97	11.46	15.97	0.25	4.54	9.09	2.75	0.55	0.56	98.89	0.80		
**LAKI 04**	-	1114	48.82	3.98	11.41	16.07	0.24	4.62	8.96	2.84	0.58	0.55	98.08	0.81		
**LAKI 04**	-	1114	49.96	3.89	11.73	15.64	0.28	4.53	9.18	2.72	0.59	0.58	99.10	0.78		
**LAKI 04**	-	1114	48.58	3.94	11.45	15.83	0.20	4.48	9.14	2.66	0.59	0.56	97.45	0.80		
**LAKI 04**	-	1114	50.24	3.95	11.37	15.96	0.30	4.67	9.17	2.64	0.58	0.56	99.43	0.80		
**LAKI 04**	-	1114	49.79	3.90	11.70	15.86	0.31	4.60	8.99	2.94	0.57	0.55	99.20	0.79		
**LAKI 04**	-	1114	49.33	3.92	11.50	16.13	0.38	4.42	9.19	2.82	0.59	0.51	98.79	0.81		
**LAKI 04**	-	1114	49.79	3.89	11.28	16.52	0.26	4.60	9.24	2.82	0.59	0.55	99.55	0.83		
**LAKI 04**	-	1114	49.29	3.84	11.68	15.94	0.34	4.49	9.20	2.63	0.58	0.59	98.57	0.80		
**LAKI 04**	-	1114	49.43	3.96	11.37	15.19	0.36	4.57	9.22	2.72	0.58	0.56	97.96	0.79		
**LAKI 04**	-	1114	49.10	4.01	11.31	15.95	0.24	4.72	9.03	2.69	0.64	0.54	98.23	0.81		
**LAKI 04**	-	1114	49.06	3.94	11.71	15.48	0.22	4.50	9.07	2.71	0.57	0.54	97.81	0.78		
**LAKI 04**	-	1114	48.22	3.98	11.53	15.58	0.36	4.44	9.07	2.92	0.56	0.57	97.24	0.81		
**LAKI 04**	-	1114	49.64	3.85	11.84	15.69	0.29	4.40	9.07	2.92	0.57	0.56	98.84	0.78		
**LAKI 04**	-	1114	48.88	3.95	11.71	15.90	0.25	4.46	9.11	2.82	0.57	0.55	98.20	0.80		
**LAKI 04**	-	1114	49.68	3.99	11.68	16.04	0.26	4.63	9.26	2.79	0.56	0.55	99.44	0.80		
**LAKI 04**	-	1114	48.77	4.06	11.38	15.15	0.27	4.42	8.99	2.76	0.55	0.55	96.90	0.78		
**LAKI 04**	-	1114	49.90	3.94	11.44	16.09	0.27	4.32	9.41	2.72	0.59	0.55	99.24	0.80		
**LAKI 04**	-	1114	49.01	4.00	11.41	15.46	0.26	4.45	9.19	2.94	0.60	0.56	97.89	0.80		
**LAKI 04**	-	1114	49.20	4.01	11.46	16.18	0.16	4.39	9.01	2.83	0.59	0.55	98.37	0.80		
**LAKI 04**	-	1114	48.73	3.97	11.61	15.97	0.33	4.69	9.20	2.81	0.59	0.53	98.44	0.82		
**LAKI 04**	-	1114	49.07	3.91	11.67	15.76	0.37	4.54	9.20	2.63	0.57	0.58	98.30	0.80		
**LAKI 04**	-	1114	49.79	3.86	11.41	15.71	0.32	4.26	9.25	2.80	0.64	0.55	98.59	0.79		
**LAKI 04**	-	1114	49.14	3.96	11.63	16.15	0.28	4.45	9.04	2.78	0.64	0.55	98.62	0.80		
**LAKI 04**	-	1114	48.71	3.95	11.66	15.38	0.26	4.63	9.05	2.81	0.61	0.59	97.66	0.79		
**LAKI 04**	-	1114	48.91	3.90	11.37	15.86	0.28	4.45	9.00	2.80	0.56	0.54	97.67	0.80		
**LAKI 04**	-	1114	49.74	3.91	11.37	15.71	0.28	4.56	9.10	2.73	0.60	0.53	98.53	0.80		
**LAKI 04**	-	1114	50.18	3.97	11.51	16.28	0.30	4.55	8.82	2.83	0.56	0.55	99.55	0.79		
**LAKI 04**	-	1114	49.39	3.90	11.34	15.96	0.09	4.63	9.24	3.03	0.55	0.57	98.69	0.82		
**LAKI 04**	-	1114	49.46	3.92	11.43	15.78	0.26	4.62	9.00	2.47	0.63	0.55	98.12	0.79		
**LAKI 04**	-	1114	48.62	3.92	11.41	15.86	0.24	4.48	9.07	3.04	0.57	0.50	97.70	0.81		
**LAKI 04**	-	1114	48.49	3.96	11.46	15.67	0.32	4.55	9.31	2.81	0.59	0.55	97.71	0.82		
**LAKI 04**	-	1114	49.43	3.91	11.60	16.18	0.45	4.61	9.03	2.98	0.53	0.56	99.28	0.81		
**LAKI 04**	-	1114	48.72	4.04	11.36	15.73	0.32	4.51	9.06	2.86	0.59	0.58	97.76	0.81		
**LAKI 04**	-	1114	49.38	3.86	11.58	15.55	0.20	4.55	8.98	2.84	0.57	0.57	98.08	0.79		
**LAKI 04**	-	1114	48.09	3.91	11.37	15.98	0.31	4.63	9.01	2.89	0.61	0.56	97.36	0.83		

**LAKI 04**	-	1114	48.59	3.95	11.52	15.63	0.22	4.45	9.14	2.77	0.60	0.52	97.40	0.80		
**LAKI 04**	-	1114	49.72	3.89	11.36	15.99	0.27	4.72	9.02	2.79	0.61	0.51	98.88	0.81		
**LAKI 04**	-	1114	48.37	3.96	11.63	15.93	0.23	4.53	9.02	2.80	0.59	0.58	97.64	0.81		
**LAKI 04**	-	1114	49.30	3.95	11.41	15.90	0.34	4.57	8.92	2.78	0.60	0.50	98.28	0.80		
**LAKI 04**	-	1114	49.22	3.89	11.19	16.06	0.27	4.81	9.16	2.83	0.63	0.55	98.61	0.83		
**LAKI 04**	-	1114	49.03	3.99	11.60	15.45	0.28	4.49	9.03	2.73	0.53	0.53	97.67	0.78		
**LAKI 04**	-	1114	48.66	4.00	11.15	15.94	0.24	4.43	9.31	2.87	0.58	0.52	97.70	0.83		
**LAKI 04**	-	1114	48.91	3.99	11.50	15.65	0.16	4.64	9.12	2.86	0.58	0.54	97.95	0.80		
**LAKI 04**	-	1114	48.76	3.94	11.80	16.26	0.28	4.73	8.97	2.71	0.61	0.50	98.56	0.81		
**LAKI 04**	-	1114	49.58	3.95	11.48	15.24	0.22	4.38	9.01	2.93	0.53	0.52	97.85	0.77		
**LAKI 04**	-	1114	48.96	4.02	11.28	15.77	0.29	4.67	9.04	2.84	0.55	0.55	97.97	0.81		
**LAKI 04**	-	1114	49.91	3.87	11.66	16.30	0.34	4.41	9.31	2.99	0.58	0.53	99.90	0.81		
**LAKI 04**	-	1114	49.14	3.95	11.51	15.65	0.16	4.44	9.19	2.44	0.55	0.52	97.57	0.78		
**LAKI 04**	-	1114	49.93	3.86	11.40	15.79	0.26	4.36	9.35	3.06	0.58	0.53	99.11	0.80		
**LAKI 04**	-	1114	49.21	3.90	11.23	15.91	0.23	4.42	8.99	2.88	0.55	0.57	97.89	0.80		
**LAKI 04**	-	1114	49.61	3.89	11.38	16.46	0.32	4.47	9.11	2.83	0.58	0.54	99.19	0.82		
**LAKI 04**	-	1114	49.52	3.96	11.68	15.78	0.12	4.67	9.08	2.82	0.58	0.49	98.70	0.79		
**LAKI 04**	-	1114	48.69	3.94	11.57	15.67	0.27	4.55	8.88	2.76	0.53	0.54	97.42	0.79		
**LAKI 04**	-	1114	48.88	3.88	11.73	16.44	0.32	4.56	8.73	2.74	0.58	0.52	98.36	0.80		
**LAKI 04**	-	1114	48.63	3.99	11.46	16.04	0.14	4.36	8.93	2.92	0.58	0.50	97.55	0.80		
**LAKI 04**	-	1114	48.45	3.96	11.75	16.08	0.25	4.69	9.18	3.06	0.55	0.52	98.48	0.82		
**LAKI 04**	-	1114	48.91	3.93	11.64	16.10	0.24	4.53	9.16	2.68	0.58	0.56	98.34	0.81		
**LAKI 04**	-	1114	49.11	3.94	11.48	16.51	0.22	4.62	9.22	2.85	0.58	0.53	99.06	0.83		
**LAKI 04**	-	1114	49.33	3.97	11.51	16.13	0.29	4.66	8.97	2.70	0.60	0.54	98.69	0.81		
**LAKI 04**	-	1114	48.59	3.96	11.64	16.12	0.31	4.58	8.92	2.76	0.61	0.54	98.02	0.81		
**LAKI 04**	-	1114	48.99	3.93	11.62	16.07	0.23	4.60	8.96	3.01	0.60	0.60	98.61	0.81		
**LAKI 04**	-	1114	49.46	3.99	11.63	15.74	0.21	4.52	8.68	3.05	0.61	0.52	98.41	0.78		
**LAKI 04**	-	1114	48.61	3.96	11.45	15.86	0.31	4.54	8.81	2.92	0.53	0.54	97.53	0.81		
**LAKI 04**	-	1114	48.72	3.97	11.33	15.94	0.33	4.49	9.09	2.72	0.57	0.52	97.69	0.81		
**LAKI 04**	-	1114	48.41	3.94	11.34	15.70	0.30	4.89	9.08	2.86	0.60	0.52	97.65	0.83		
**LAKI 04**	-	1114	48.51	3.87	11.77	16.00	0.30	4.62	8.96	2.84	0.60	0.53	97.99	0.81		
**LAKI 04**	-	1114	48.81	3.87	11.32	16.03	0.27	4.45	8.91	2.74	0.58	0.53	97.51	0.81		
**LAKI 04**	-	1114	49.24	3.97	11.49	16.00	0.25	4.75	8.99	2.70	0.58	0.52	98.49	0.81		
**LAKI 04**	-	1114	48.83	3.88	11.10	16.04	0.31	4.63	9.02	2.81	0.56	0.54	97.73	0.83		
**LAKI 04**	-	1114	48.88	3.90	11.18	16.12	0.31	4.73	9.00	2.77	0.60	0.52	98.02	0.83		
**LAKI 04**	-	1114	48.73	3.99	11.22	16.42	0.24	4.51	9.00	2.75	0.59	0.50	97.95	0.82		
**LAKI 04**	-	1114	49.12	3.92	11.41	16.05	0.25	4.64	9.08	2.98	0.55	0.56	98.57	0.82		
**LAKI 04**	-	1114	50.33	3.99	11.65	15.67	0.30	4.44	9.14	2.92	0.54	0.56	99.54	0.78		
**LAKI 04**	-	1114	49.46	3.91	11.59	16.79	0.29	4.51	9.07	2.65	0.55	0.54	99.36	0.81		
**LAKI 04**	-	1114	49.81	3.94	11.51	15.86	0.22	4.48	9.02	2.73	0.57	0.50	98.63	0.79		
**LAKI 04**	-	1114	48.84	3.99	11.41	15.97	0.31	4.44	8.97	2.78	0.61	0.55	97.86	0.80		
**LAKI 04**	-	1114	49.47	3.94	11.28	15.91	0.21	4.43	9.32	2.91	0.56	0.58	98.60	0.81		
**LAKI 04**	-	1114	49.30	4.03	11.76	15.83	0.17	4.61	9.01	2.78	0.56	0.57	98.62	0.79		
**LAKI 04**	-	1114	49.68	3.92	11.77	15.87	0.24	4.46	8.99	2.63	0.57	0.57	98.69	0.78		

**LAKI 04**	-	1114	48.52	3.90	11.43	15.74	0.19	4.57	9.13	2.34	0.59	0.59	97.02	0.80		
**LAKI 04**	-	1114	49.75	4.01	11.49	15.08	0.31	4.63	9.22	2.26	0.60	0.56	97.91	0.77		
**LAKI 04**	-	1114	49.00	4.02	11.24	16.48	0.29	4.63	9.07	2.92	0.60	0.53	98.79	0.84		
**LAKI 04**	-	1114	49.91	3.96	11.29	16.05	0.36	4.44	9.21	2.87	0.53	0.57	99.19	0.81		
**LAKI 04**	-	1114	50.06	4.00	11.48	15.92	0.28	4.71	8.89	2.60	0.59	0.57	99.09	0.79		
**LAKI 04**	-	1114	48.58	3.94	11.52	15.84	0.15	4.66	9.13	3.00	0.59	0.54	97.94	0.82		
**LAKI 04**	-	1114	48.12	3.88	11.33	15.81	0.31	4.57	9.10	2.83	0.55	0.54	97.04	0.82		
**LAKI 04**	-	1114	48.16	3.92	11.51	15.79	0.42	4.33	9.03	2.73	0.59	0.54	97.02	0.80		
**LAKI 04**	-	1114	49.12	3.93	11.52	16.21	0.26	4.61	8.98	2.70	0.61	0.51	98.46	0.81		
**LAKI 04**	-	1114	48.86	3.89	11.60	16.15	0.31	4.63	8.92	2.87	0.62	0.53	98.40	0.81		
**LAKI 04**	-	1114	48.45	3.97	11.28	15.96	0.32	4.49	9.07	2.88	0.57	0.54	97.53	0.82		
**LAKI 04**	-	1114	48.55	3.93	11.56	15.38	0.37	4.64	8.93	2.78	0.57	0.60	97.32	0.80		
**LAKI 04**	-	1114	48.60	3.93	11.22	16.14	0.26	4.49	9.03	2.78	0.58	0.49	97.52	0.82		
**LAKI 04**	-	1114	48.90	3.88	11.54	15.83	0.19	4.64	8.84	2.84	0.61	0.53	97.80	0.80		
**LAKI 04**	-	1114	48.77	4.01	11.25	15.77	0.22	4.58	8.98	2.83	0.60	0.51	97.53	0.81		
**LAKI 04**	-	1114	49.36	3.90	11.34	15.59	0.31	4.80	8.86	2.71	0.62	0.53	98.01	0.80		
**LAKI 04**	-	1114	48.79	3.99	11.40	16.06	0.28	4.66	8.74	2.83	0.65	0.53	97.93	0.81		
**LAKI 04**	-	1114	49.20	3.95	11.62	15.98	0.17	4.46	8.91	2.73	0.56	0.54	98.12	0.79		
**LAKI 04**	-	1114	48.85	3.93	11.34	15.96	0.24	4.41	9.12	2.71	0.55	0.56	97.67	0.81		
**LAKI 04**	-	1114	49.07	4.08	11.44	15.85	0.25	4.60	8.98	2.51	0.53	0.56	97.87	0.79		
**LAKI 04**	-	1114	48.89	3.92	11.18	15.74	0.29	4.56	9.02	2.90	0.58	0.56	97.66	0.82		
**LAKI 04**	-	1114	49.53	3.96	11.31	15.67	0.26	4.73	9.03	2.94	0.56	0.56	98.55	0.81		
**LAKI 04**	-	1114	49.06	3.95	11.41	15.82	0.45	4.54	8.97	2.79	0.54	0.55	98.09	0.81		
**LAKI 04**	-	1114	49.25	4.03	11.55	16.01	0.18	4.50	9.23	2.98	0.63	0.54	98.91	0.81		
**LAKI 04**	-	1114	48.97	4.04	11.35	15.84	0.24	4.53	9.23	2.23	0.58	0.54	97.55	0.79		
**LAKI 04**	-	1114	48.88	3.88	11.61	16.08	0.29	4.70	8.94	2.61	0.54	0.53	98.07	0.81		
**LAKI 04**	-	1114	49.60	4.02	11.14	15.98	0.32	4.54	9.25	2.73	0.59	0.57	98.73	0.82		
**LAKI 04**	-	1114	49.66	3.98	11.45	15.91	0.37	4.50	9.08	2.99	0.57	0.56	99.08	0.80		
**LAKI 04**	-	1114	49.39	4.00	11.62	16.24	0.32	4.52	8.95	2.94	0.63	0.54	99.15	0.81		
Averages		1114	*49.24*	*3.94*	*11.46*	*15.92*	*0.27*	*4.55*	*9.07*	*2.79*	*0.58*	*0.55*	*98.37*	*0.80*	*2.76*	
**LAKI 04 Fe-rich glass**																
**LAKI 04**	-	1114	49.65	4.25	10.62	17.13	0.28	5.09	9.26	2.56	0.55	0.60	100.00	0.88		
**LAKI 04**	-	1114	48.43	4.42	8.94	18.16	0.29	5.38	9.32	2.15	0.58	0.61	98.28	0.98		
**LAKI 04**	-	1114	45.49	5.36	6.70	21.74	0.35	6.09	9.89	1.62	0.42	0.71	98.37	1.25		
**LAKI 04**	-	1114	49.21	4.23	9.79	17.40	0.24	4.98	9.24	2.47	0.53	0.59	98.68	0.91		
**LAKI 04**	-	1114	48.19	4.42	9.34	18.12	0.34	5.33	9.19	2.35	0.53	0.60	98.42	0.97		
**LAKI 04**	-	1114	46.33	4.58	8.67	19.14	0.28	5.23	9.79	2.23	0.49	0.63	97.38	1.06		
**LAKI 04**	-	1114	46.00	5.28	7.32	20.81	0.31	5.96	9.80	2.09	0.38	0.72	98.67	1.19		
**LAKI 04**	-	1114	47.32	4.55	9.83	18.57	0.29	4.54	9.18	2.24	0.52	0.54	97.57	0.93		
**LAKI 04**	-	1114	47.87	5.66	7.61	19.18	0.26	7.22	10.65	1.78	0.42	0.74	101.38	1.17		
**LAKI 04**	-	1114	48.21	4.49	8.67	18.62	0.29	5.40	9.24	2.21	0.59	0.61	98.34	1.01		
**LAKI 04**	-	1114	47.68	5.46	7.35	21.75	0.36	6.03	9.85	1.77	0.39	0.72	101.35	1.18		
**LAKI 04**	-	1114	48.09	4.65	8.89	18.29	0.22	5.30	9.11	2.22	0.54	0.63	97.93	0.98		
**LAKI 04**	-	1114	46.71	5.15	8.20	20.34	0.31	5.69	9.75	1.95	0.44	0.68	99.23	1.11		

Averages		*47.63*	*4.81*	*8.61*	*19.17*	*0.29*	*5.56*	*9.56*	*2.13*	*0.49*	*0.65*	*98.89*	*1.05*	*2.84*	

Table 2 *Measurements of Fe-rich CBL thickness and plagioclase outer rim thickness, made on BSE images. Note that the grains are randomly oriented, so these thicknesses are likely to be greater than the true 3D*

thickness.

Sample	Depth in drill core (m)	Plag major axis (um)	Plag minor axis (um)	Aspect ratio	Fe-rich rim perpendicular to (001) (um)	Fe-rich rim perpendicular to (010) (um)	Ratio	Plag rim perpendicular to (001) (um)	Plag rim perpendicular to (010) (um)	Ratio
**KI76 149**	45.4	91.1	28.7	3.2	1.4	0.7	1.9	1.5	0.6	2.6
**KI76 149**	45.4	74.2	25.9	2.9	1.2	0.6	2.1	1.5	0.6	2.6
**KI76 149**	45.4	57.4	13.8	4.2	0.8	0.4	2.0	1.1	0.3	3.3
**KI76 149**	45.4	50.8	25.4	2.0	0.9	0.5	2.0	1.3	0.5	2.5
**KI76 149**	45.4	58.9	26.1	2.3	0.9	0.9	1.0	0.9	1.3	0.7
**KI76 149**	45.4	66.3	17.6	3.8	1.0	0.7	1.6	1.7	0.4	4.0
**KI76 149**	45.4	40.6	15.5	2.6	1.5	0.6	2.5	1.6	1.0	1.6
**KI76 149**	45.4	112.0	26.2	4.3	1.5	0.7	2.2	1.4	1.1	1.2
**KI76 149**	45.4	51.2	7.7	6.6	0.4	0.3	1.3	1.2	0.4	3.6
**KI76 149**	45.4	85.8	7.0	12.2	1.2	0.3	3.3	1.2	0.4	3.2
**KI76 149**	45.4	89.8	16.1	5.6	0.9	0.5	1.9	1.5	0.7	2.2
**KI76 149**	45.4	65.9	21.0	3.1	0.7	0.5	1.4	1.1	0.6	1.8
**KI76 149**	45.4	65.9	21.0	3.1	0.7	0.3	2.2	1.1	0.4	2.5
**KI76 149**	45.4	43.6	20.7	2.1	1.0	0.6	1.6	1.2	0.7	1.7
**KI76 149**	45.4	43.6	20.7	2.1	1.0	0.4	2.4	1.2	0.6	2.1
**KI76 149**	45.4	45.6	16.4	2.8	0.8	0.5	1.8	0.7	0.3	2.2
**KI76 149**	45.4	33.9	14.6	2.3	1.0	0.5	2.0	1.4	0.6	2.1
**KI76 149**	45.4	53.1	18.5	2.9	1.2	0.4	3.0	1.4	0.4	3.3
**KI76 149**	45.4	62.4	15.7	4.0	2.1	0.4	4.9	3.3	0.6	6.1
**KI76 149**	45.4	48.5	14.6	3.3	0.9	0.4	2.1	0.9	0.4	2.2
**KI76 149**	45.4	86.9	9.6	9.1	0.5	0.3	1.5	0.8	0.3	2.7
**KI76 149**	45.4	37.9	6.1	6.3	0.8	0.5	1.6	1.2	0.5	2.3
**KI76 149**	45.4	46.5	12.1	3.8	0.8	0.4	1.9	1.0	0.4	2.8
**KI76 149**	45.4	42.6	11.9	3.6	0.7	0.6	1.2	2.5	0.5	5.5
**KI76 149**	45.4	37.2	8.2	4.6	1.1	0.5	2.1	1.5	0.2	6.2
**KI76 149**	45.4	67.1	19.6	3.4	1.0	0.9	1.1	2.0	1.0	1.9
**KI76 149**	45.4	33.9	6.1	5.6	0.8	0.5	1.6	1.5	0.6	2.6
**KI76 149**	45.4	53.3	16.9	3.1	0.6	0.5	1.3	0.6	0.5	1.3
**KI76 149**	45.4	99.1	23.6	4.2	1.0	0.5	1.8	1.3	0.5	2.9
**KI76 149**	45.4	52.1	18.5	2.8	0.9	0.5	1.9	1.0	0.4	2.4
**KI76 149**	45.4	31.1	11.6	2.7	0.9	0.6	1.5	0.8	0.7	1.2
**KI76 149**	45.4	131.2	26.2	5.0	2.0	0.5	3.8	2.3	0.7	3.2
**KI76 149**	45.4	40.5	16.1	2.5	0.9	0.5	1.9	1.3	0.8	1.7
**KI76 149**	45.4	91.4	17.2	5.3	0.5	0.5	1.0	1.0	0.6	1.6
**KI76 149**	45.4	85.9	14.3	6.0	0.8	0.7	1.3	1.3	0.6	2.2
**KI76 149**	45.4	50.4	17.9	2.8	0.7	0.5	1.6	0.5	0.6	0.8
**KI76 149**	45.4	97.8	16.3	6.0	0.5	0.4	1.1	0.5	0.5	1.0
**KI76 149**	45.4	69.4	12.7	5.5	0.7	0.4	1.6	1.2	0.5	2.5
**KI76 149**	45.4	46.2	23.1	2.0	0.7	0.9	0.8	0.9	1.1	0.8
**KI76 149**	45.4	148.6	14.9	9.9	1.0	0.3	3.2	1.5	0.4	3.9
**KI76 149**	45.4	37.2	15.2	2.5	1.2	0.5	2.3	1.1	0.6	1.8

**KI76 149**	45.4	61.1	23.7	2.6	1.2	0.4	3.3	1.4	0.7	2.1
**KI76 149**	45.4	73.4	13.0	5.7	0.9	0.4	2.4	1.9	0.6	3.3
**KI76 149**	45.4	69.2	38.2	1.8	0.9	0.8	1.1	1.1	0.7	1.6
**KI76 149**	45.4	134.8	19.4	6.9	1.0	0.4	2.7	0.9	0.5	1.7
**KI76 149**	45.4	83.2	15.6	5.3	1.2	0.4	3.3	1.3	0.2	5.6
**KI76 149**	45.4	39.4	9.7	4.1	0.9	0.3	2.9	0.7	0.4	1.7
**KI76 149**	45.4	56.4	15.8	3.6	1.0	0.5	1.9	1.0	0.5	2.1
KI76 149 Average	*45.4*	*65.5*	*17.2*	*4.2*	*1.0*	*0.5*	*2.0*	*1.3*	*0.6*	*2.5*
**KI76 147**	44.8	90.1	15.4	5.8	0.7	0.6	1.2	0.6	0.3	1.9
**KI76 147**	44.8	80.2	13.6	5.9	0.6	0.4	1.4	0.6	0.2	2.6
**KI76 147**	44.8	69.6	8.5	8.2	0.6	0.3	2.0	1.1	0.5	2.0
**KI76 147**	44.8	48.6	11.7	4.2	0.7	0.3	2.6	1.2	0.4	3.4
**KI76 147**	44.8	0.0	17.7	0.0	1.3	0.3	4.0	1.6	0.5	3.5
**KI76 147**	44.8	18.0	13.7	1.3	0.7	0.7	1.0	0.6	0.8	0.8
**KI76 147**	44.8	18.0	13.7	1.3	0.7	0.4	1.9	0.6	0.7	0.9
**KI76 147**	44.8	52.5	12.2	4.3	0.5	0.3	1.6	1.0	0.4	2.6
**KI76 147**	44.8	48.6	13.7	3.6	1.2	0.3	3.7	1.3	0.4	3.7
**KI76 147**	44.8	28.1	9.0	3.1	0.7	0.3	2.0	1.0	0.5	2.0
**KI76 147**	44.8	30.4	9.9	3.1	0.8	0.4	2.1	1.1	0.7	1.5
**KI76 147**	44.8	74.3	21.8	3.4	0.4	0.2	1.9	0.8	0.3	2.5
**KI76 147**	44.8	35.3	17.0	2.1	0.6	0.4	1.6	0.9	0.3	3.0
**KI76 147**	44.8	130.9	45.6	2.9	0.6	0.3	2.4	1.5	0.3	5.4
**KI76 147**	44.8	121.5	29.6	4.1	0.6	0.2	2.7	0.9	0.3	2.8
**KI76 147**	44.8	70.0	7.2	9.8	0.6	0.3	2.2	1.2	0.4	3.1
**KI76 147**	44.8	69.0	12.1	5.7	0.5	0.2	2.5	0.8	0.4	2.0
**KI76 147**	44.8	72.2	18.1	4.0	0.4	0.2	2.3	0.5	0.2	3.1
**KI76 147**	44.8	195.9	49.8	3.9	0.8	0.3	2.6	0.9	0.3	3.2
**KI76 147**	44.8	175.3	19.2	9.1	1.0	0.2	4.3	1.2	0.3	4.0
**KI76 147**	44.8	145.8	14.2	10.3	0.5	0.2	2.7	1.1	0.4	2.5
**KI76 147**	44.8	51.5	8.8	5.8	1.4	0.2	7.0	2.3	0.2	9.3
**KI76 147**	44.8	50.4	8.2	6.1	1.0	1.4	0.7	0.3	0.5	0.6
**KI76 147**	44.8	159.0	34.3	4.6	0.5	1.5	0.4	0.5	0.5	0.9
**KI76 147**	44.8	61.2	18.6	3.3	0.7	0.5	1.4	0.8	0.6	1.3
**KI76 147**	44.8	97.7	30.1	3.2	0.3	0.2	1.3	0.6	0.4	1.3
**KI76 147**	44.8	67.8	11.6	5.8	0.7	0.4	1.8	0.2	0.3	0.7
**KI76 147**	44.8	86.9	24.1	3.6	0.6	0.2	2.8	1.0	0.4	2.4
**KI76 147**	44.8	45.7	21.6	2.1	0.5	0.2	1.9	0.8	0.3	2.5
**KI76 147**	44.8	46.6	7.0	6.7	0.9	0.2	5.0	0.9	0.4	2.1
**KI76 147**	44.8	152.3	17.2	8.9	0.7	0.3	2.7	1.0	0.3	3.5
**KI76 147**	44.8	137.0	14.9	9.2	0.4	0.4	1.1	0.9	0.6	1.5
**KI76 147**	44.8	38.5	10.6	3.6	0.9	0.1	6.2	0.7	0.3	2.3
**KI76 147**	44.8	53.6	10.2	5.2	0.6	0.3	2.2	0.9	0.3	3.2

**KI76 147**	44.8	107.8	19.9	5.4	0.6	0.4	1.4	1.0	0.0	-
**KI76 147**	44.8	101.6	23.7	4.3	0.3	0.3	1.3	0.8	0.2	5.0
**KI76 147**	44.8	229.5	19.5	11.8	0.5	0.2	2.3	1.0	-	-
**KI76 147**	44.8	233.2	17.0	13.7	0.6	0.2	2.7	0.9	-	-
**KI76 147**	44.8	46.8	13.0	3.6	0.2	0.2	1.2	0.6	0.3	2.0
**KI76 147**	44.8	48.7	13.5	3.6	0.7	0.3	2.5	1.0	0.4	2.6
**KI76 147**	44.8	47.7	8.2	5.9	0.8	0.2	3.8	1.5	0.4	4.3
**KI76 147**	44.8	97.5	30.8	3.2	0.7	0.2	2.7	1.9	0.3	6.7
**KI76 147**	44.8	86.9	18.7	4.7	1.0	0.4	2.4	1.4	0.3	4.4
**KI76 147**	44.8	82.6	8.7	9.4	0.6	0.2	2.7	0.8	0.4	1.8
**KI76 147**	44.8	73.8	9.6	7.6	0.7	0.2	3.0	0.9	0.3	3.0
**KI76 147**	44.8	86.8	13.2	6.6	0.5	0.3	1.9	0.8	0.5	1.7
**KI76 147**	44.8	86.0	35.4	2.4	0.4	0.2	1.7	0.6	0.4	1.6
**KI76 147**	44.8	63.3	15.0	4.2	0.5	0.3	1.5	0.9	0.4	2.3
**KI76 147**	44.8	67.0	10.1	6.7	0.4	0.2	2.9	0.5	0.3	1.6
**KI76 147**	44.8	58.5	8.0	7.3	0.5	0.3	1.6	0.7	-	-
**KI76 147**	44.8	60.4	8.8	6.8	0.3	0.3	1.0	0.7	0.3	2.1
KI76 147 Average	*44.8*	*82.4*	*16.9*	*5.3*	*0.6*	*0.3*	*2.3*	*0.9*	*0.4*	*2.7*
**KI76 145**	44.3	85.9	21.2	4.1	0.4	0.1	5.0	0.7	-	-
**KI76 145**	44.3	80.8	19.9	4.1	0.2	0.1	1.7	0.9	0.3	3.4
**KI76 145**	44.3	33.4	12.6	2.7	0.2	0.2	0.9	0.4	0.4	0.8
**KI76 145**	44.3	43.7	12.9	3.4	0.6	0.4	1.4	1.1	0.5	2.3
**KI76 145**	44.3	23.1	10.6	2.2	0.2	0.2	1.6	0.8	0.1	7.6
**KI76 145**	44.3	26.9	4.6	5.8	0.5	0.1	4.3	0.7	-	-
**KI76 145**	44.3	80.9	18.7	4.3	0.3	0.1	2.8	0.5	-	-
**KI76 145**	44.3	26.9	9.9	2.7	0.3	0.2	2.0	0.6	0.3	2.4
**KI76 145**	44.3	44.4	4.0	11.1	0.2	0.2	1.3	0.4	0.2	2.5
**KI76 145**	44.3	53.9	7.2	7.5	0.4	0.1	2.9	0.5	0.2	2.6
**KI76 145**	44.3	72.7	29.4	2.5	0.7	0.4	1.8	1.3	0.4	3.4
**KI76 145**	44.3	219.2	17.3	12.6	0.4	0.1	3.2	0.6	0.0	0.0
**KI76 145**	44.3	201.4	22.8	8.8	0.5	0.3	2.1	0.8	0.5	1.7
**KI76 145**	44.3	109.3	15.3	7.2	0.4	0.2	2.6	0.6	-	-
**KI76 145**	44.3	191.2	17.7	10.8	0.4	0.1	2.5	0.6	0.3	2.5
**KI76 145**	44.3	113.2	20.9	5.4	0.3	0.2	1.5	0.8	0.4	1.9
**KI76 145**	44.3	109.4	21.1	5.2	0.4	0.2	1.8	1.2	0.5	2.6
**KI76 145**	44.3	54.2	16.2	3.3	0.2	0.2	1.3	0.4	0.2	1.8
**KI76 145**	44.3	91.3	9.2	9.9	0.3	0.2	1.9	0.7	0.3	2.2
**KI76 145**	44.3	66.8	14.9	4.5	0.4	0.1	3.2	0.3	0.3	1.1
**KI76 145**	44.3	142.1	22.6	6.3	0.3	0.1	2.4	0.6	0.3	1.6
**KI76 145**	44.3	93.8	13.7	6.9	0.2	0.1	1.1	0.2	0.4	0.6
**KI76 145**	44.3	57.1	8.4	6.8	0.4	0.1	4.8	0.7	0.2	3.7
**KI76 145**	44.3	123.4	20.1	6.1	0.4	0.2	2.1	0.5	-	-

**KI76 145**	44.3	25.9	7.2	3.6	0.4	0.2	2.3	1.2	0.3	3.9
**KI76 145**	44.3	75.6	5.8	13.2	0.3	0.1	2.7	0.5	0.3	1.6
**KI76 145**	44.3	24.0	4.8	5.0	0.3	0.2	1.6	0.5	0.4	1.3
**KI76 145**	44.3	57.4	16.8	3.4	0.2	0.1	1.3	0.4	0.4	1.1
**KI76 145**	44.3	144.8	22.3	6.5	0.2	0.2	0.9	0.3	0.3	1.0
**KI76 145**	44.3	23.5	27.6	0.9	0.2	0.2	1.2	0.5	-	-
**KI76 145**	44.3	128.6	16.9	7.6	0.5	0.1	3.1	0.6	0.3	2.2
**KI76 145**	44.3	45.2	12.8	3.5	0.5	0.3	1.4	1.3	0.0	0.0
**KI76 145**	44.3	26.9	7.8	3.4	0.2	0.2	0.9	0.4	0.3	1.2
**KI76 145**	44.3	38.4	13.3	2.9	0.2	0.1	1.6	0.4	0.3	1.6
**KI76 145**	44.3	150.5	13.0	11.5	0.5	0.2	3.5	0.8	0.3	2.8
**KI76 145**	44.3	178.4	19.2	9.3	0.5	0.2	3.6	0.7	-	-
**KI76 145**	44.3	109.7	26.0	4.2	0.6	0.1	4.8	0.9	-	-
**KI76 145**	44.3	78.6	5.7	13.7	0.3	0.1	2.2	0.4	-	-
**KI76 145**	44.3	31.4	13.6	2.3	0.3	0.1	3.0	0.4	0.4	1.1
**KI76 145**	44.3	23.0	6.9	3.3	0.5	0.2	3.6	1.0	0.3	3.1
**KI76 145**	44.3	65.9	9.1	7.2	0.7	0.1	4.6	1.0	0.2	5.0
**KI76 145**	44.3	126.9	34.4	3.7	0.2	0.1	1.9	0.3	0.3	0.8
**KI76 145**	44.3	49.4	3.4	14.5	0.4	0.1	3.0	1.1	0.2	5.1
**KI76 145**	44.3	50.9	15.6	3.3	0.2	0.2	1.2	0.4	0.4	1.1
**KI76 145**	44.3	61.9	14.9	4.1	0.2	0.1	1.4	0.4	-	-
**KI76 145**	44.3	94.0	18.6	5.1	0.3	0.1	3.9	0.6	0.4	1.5
**KI76 145**	44.3	52.6	12.4	4.3	0.3	0.2	1.8	0.5	0.2	2.0
**KI76 145**	44.3	64.8	11.9	5.5	0.3	0.2	2.0	0.7	0.4	2.0
**KI76 145**	44.3	109.1	10.8	10.1	0.6	0.3	2.1	0.4	0.5	0.7
**KI76 145**	44.3	76.4	11.5	6.7	0.3	0.2	1.5	0.4	0.4	1.2
**KI76 145**	44.3	56.7	10.0	5.7	0.5	0.1	3.6	1.4	-	-
**KI76 145**	44.3	50.7	7.7	6.6	0.9	0.1	9.7	1.7	-	-
**KI76 145**	44.3	42.4	7.5	5.7	0.4	0.1	2.8	0.5	-	-
**KI76 145**	44.3	65.9	13.3	5.4	0.3	0.2		0.6	0.3	2.2
**KI76 145**	44.3	79.4	14.3	6.1	0.4	0.2	2.5	0.7	0.2	2.9
KI76 145 Average	*44.3*	*79.2*	*14.3*	*6.0*	*0.4*	*0.2*	*2.5*	*0.7*	*0.3*	*2.1*
**KI81 250**	76.1	50.9	10.5	4.9	0.5	0.3	1.5	0.5	0.3	1.6
**KI81 250**	76.1	14.6	6.0	2.4	1.6	0.4	4.1	1.5	0.4	4.2
**KI81 250**	76.1	56.1	14.1	4.0	0.7	0.0	142.0	1.0	0.6	1.8
**KI81 250**	76.1	0.0	21.5	-	0.6	0.4	1.6	0.9	0.4	2.3
**KI81 250**	76.1	51.0	22.1	2.3	0.9	0.3	2.8	0.9	0.4	2.4
**KI81 250**	76.1	21.1	9.8	2.2	0.6	0.2	2.9	0.8	0.5	1.5
**KI81 250**	76.1	26.8	13.3	2.0	0.8	0.6	1.2	0.9	0.5	1.7
**KI81 250**	76.1	0.0	21.3	-	0.6	0.4	1.3	1.1	0.8	1.3
**KI81 250**	76.1	48.6	6.0	8.2	1.3	0.3	4.9	0.8	0.3	2.4
**KI81 250**	76.1	35.1	14.8	2.4	0.8	0.3	2.5	0.9	0.5	1.8

**KI81 250**	76.1	0.0	11.1	-	0.8	0.3	2.7	1.0	0.3	3.9
**KI81 250**	76.1	0.0	15.1	-	1.2	0.3	4.4	0.5	0.4	1.4
**KI81 250**	76.1	35.0	13.6	2.6	0.5	0.4	1.4	0.6	0.4	1.4
**KI81 250**	76.1	0.0	6.4	-	0.7	0.3	2.3	1.2	0.3	3.7
**KI81 250**	76.1	13.1	7.3	1.8	0.5	0.2	2.1	0.5	0.3	2.0
**KI81 250**	76.1	30.7	11.0	2.8	0.6	0.6	1.2	1.2	0.7	1.7
**KI81 250**	76.1	18.4	6.5	2.8	0.4	0.3	1.6	0.6	0.3	2.3
**KI81 250**	76.1	30.0	11.5	2.6	0.8	0.3	2.9	0.9	0.3	3.3
**KI81 250**	76.1	38.1	7.0	5.4	0.6	0.2	2.6	0.8	0.4	2.1
**KI81 250**	76.1	42.9	19.2	2.2	0.5	0.4	1.5	0.9	0.5	2.0
**KI81 250**	76.1	62.4	12.1	5.1	1.5	0.3	5.2	1.1	0.4	3.1
**KI81 250**	76.1	33.0	25.4	1.3	0.3	0.3	1.1	0.6	0.9	0.8
**KI81 250**	76.1	26.9	10.7	2.5	0.4	0.3	1.7	0.5	0.4	1.3
**KI81 250**	76.1	72.1	9.4	7.7	1.2	0.3	4.7	1.8	0.3	5.7
**KI81 250**	76.1	49.6	24.9	2.0	0.7	0.4	1.6	1.0	0.6	1.5
**KI81 250**	76.1	63.9	22.6	2.8	0.8	0.4	1.8	1.1	0.4	2.4
**KI81 250**	76.1	74.8	15.0	5.0	0.5	0.5	1.1	0.6	0.5	1.1
**KI81 250**	76.1	38.9	16.0	2.4	0.6	0.3	1.8	0.7	0.4	2.0
**KI81 250**	76.1	58.1	11.6	5.0	0.9	0.3	3.1	1.0	0.3	2.9
**KI81 250**	76.1	0.0	11.1	-	0.4	0.2	1.7	0.5	0.3	1.7
**KI81 250**	76.1	29.2	27.3	1.1	0.7	0.9	0.8	0.7	0.9	0.8
**KI81 250**	76.1	43.8	20.6	2.1	1.4	0.6	2.5	0.8	0.5	1.6
**KI81 250**	76.1	0.0	12.5	-	0.5	0.5	0.9	0.7	0.4	1.6
**KI81 250**	76.1	0.0	12.8	-	0.7	0.3	2.7	1.1	0.3	4.0
**KI81 250**	76.1	51.9	11.2	4.6	1.2	0.5	2.5	1.3	0.3	3.6
**KI81 250**	76.1	17.4	9.7	1.8	0.4	0.3	1.3	0.7	0.3	2.1
**KI81 250**	76.1	27.4	11.1	2.5	0.5	0.3	1.6	0.7	0.5	1.4
**KI81 250**	76.1	19.3	11.6	1.7	0.4	0.4	1.0	1.0	0.3	2.8
**KI81 250**	76.1	59.1	14.2	4.2	1.1	0.5	2.0	1.3	0.5	2.5
**KI81 250**	76.1	0.0	12.0	-	0.8	0.4	2.0	0.7	0.4	1.8
**KI81 250**	76.1	0.0	10.1	-	0.7	0.3	2.4	1.3	0.3	3.9
**KI81 250**	76.1	16.1	9.5	1.7	0.5	0.3	1.6	0.7	0.2	2.7
**KI81 250**	76.1	29.8	7.4	4.0	0.7	0.3	2.4	0.8	0.3	2.6
**KI81 250**	76.1	16.4	5.2	3.2	0.6	0.2	2.7	0.7	0.3	2.3
**KI81 250**	76.1	15.2	5.4	2.8	0.6	0.2	2.4	0.6	0.3	2.1
**KI81 250**	76.1	0.0	19.1	-	0.6	0.3	2.5	0.9	0.2	3.6
**KI81 250**	76.1	51.5	20.5	2.5	0.4	0.3	1.5	0.8	0.4	2.2
**KI81 250**	76.1	0.0	15.5	-	0.5	0.3	2.0	0.6	0.3	2.0
**KI81 250**	76.1	0.0	12.5	-	0.9	0.4	2.1	1.0	0.5	1.9
**KI81 250**	76.1	0.0	8.3	-	0.8	0.3	2.5	0.8	0.4	2.2
**KI81 250**	76.1	38.1	11.1	3.4	0.6	0.2	2.3	0.8	0.3	2.7
**KI81 250**	76.1	0.0	12.1	-	1.1	0.5	2.4	1.4	0.5	2.9
**KI81 250**	76.1	37.3	17.0	2.2	0.9	0.2	4.2	0.6	0.4	1.7
**KI81 250**	76.1	17.0	5.8	2.9	0.7	0.5	1.4	1.0	0.3	3.1

**KI81 250**	76.1	36.4	18.3	2.0	0.6	0.6	1.1	1.0	0.7	1.6
**KI81 250**	76.1	34.3	15.3	2.2	0.6	0.3	1.9	0.8	0.3	2.3
**KI81 250**	76.1	25.5	5.5	4.6	0.7	0.2	2.8	0.9	0.3	3.0
**KI81 250**	76.1	19.0	8.2	2.3	1.1	0.4	2.9	1.0	0.3	2.9
KI81 250 Average	*76.1*	*27.2*	*13.1*	*3.1*	*0.7*	*0.3*	*4.6*	*0.9*	*0.4*	*2.3*
**KI81 220**	67.0	32.5	18.7	1.7	0.7	0.7	1.0	0.8	0.7	1.0
**KI81 220**	67.0	0.0	7.7	-	0.5	0.5	1.1	0.5	0.4	1.2
**KI81 220**	67.0	0.0	12.7	-	0.8	0.5	1.8	0.7	0.4	1.7
**KI81 220**	67.0	0.0	16.1	-	0.8	0.3	2.3	0.8	0.4	2.1
**KI81 220**	67.0	0.0	0.0	-	1.0	0.7	1.4	1.5	0.7	2.3
**KI81 220**	67.0	0.0	9.3	-	1.1	0.4	3.0	1.0	0.5	1.7
**KI81 220**	67.0	0.0	0.0	-	1.9	0.6	3.4	1.4	0.6	2.5
**KI81 220**	67.0	0.0	0.0	-	0.5	0.5	0.9	1.2	0.5	2.5
**KI81 220**	67.0	0.0	16.2	-	1.0	0.4	2.5	0.8	0.5	1.7
**KI81 220**	67.0	0.0	5.6	-	0.5	0.4	1.4	1.4	0.4	3.7
**KI81 220**	67.0	0.0	13.0	-	0.7	0.3	2.0	1.1	0.3	3.3
**KI81 220**	67.0	37.4	13.6	2.8	0.6	0.4	1.6	0.9	0.5	1.7
**KI81 220**	67.0	0.0	9.0	-	0.5	0.6	0.9	0.8	0.6	1.4
**KI81 220**	67.0	0.0	13.1	-	0.5	0.5	0.9	1.2	0.4	2.6
**KI81 220**	67.0	0.0	9.9	-	0.6	0.5	1.4	1.0	0.5	2.1
**KI81 220**	67.0	0.0	7.6	-	0.8	0.5	1.8	0.5	0.3	1.5
**KI81 220**	67.0	0.0	24.9	-	1.6	0.5	3.0	1.5	0.4	3.9
**KI81 220**	67.0	0.0	6.2	-	1.0	0.3	2.9	0.8	0.4	2.2
**KI81 220**	67.0	19.8	12.8	1.5	0.9	0.8	1.1	0.8	1.3	0.6
**KI81 220**	67.0	54.0	11.7	4.6	0.8	0.7	1.2	1.2	0.6	2.0
**KI81 220**	67.0	42.8	28.9	1.5	1.2	0.6	2.0	1.2	0.8	1.5
**KI81 220**	67.0	0.0	9.2	-	0.6	0.5	1.3	0.7	0.4	1.7
**KI81 220**	67.0	0.0	6.7	-	0.8	0.4	2.2	0.9	0.4	2.2
**KI81 220**	67.0	33.0	13.1	2.5	0.8	0.6	1.3	1.4	0.4	3.4
**KI81 220**	67.0	37.0	18.3	2.0	1.0	0.5	1.9	1.5	0.7	2.2
**KI81 220**	67.0	0.0	14.0	-	1.4	0.4	3.3	1.4	0.4	3.7
**KI81 220**	67.0	27.0	8.6	3.2	1.0	0.9	1.1	0.8	0.4	2.3
**KI81 220**	67.0	0.0	11.3	-	1.1	0.4	3.2	0.8	0.4	2.2
**KI81 220**	67.0	0.0	31.2	-	1.2	0.7	1.6	1.0	0.9	1.1
**KI81 220**	67.0	0.0	21.5	-	0.7	0.8	1.0	0.8	0.8	1.0
**KI81 220**	67.0	24.3	13.7	1.8	0.5	0.5	0.9	0.9	0.7	1.3
**KI81 220**	67.0	31.5	7.4	4.3	1.2	0.5	2.3	1.1	0.5	2.0
**KI81 220**	67.0	18.0	4.6	3.9	1.1	0.6	1.9	1.2	0.5	2.7
**KI81 220**	67.0	0.0	5.6	-	0.8	0.5	1.4	0.8	0.8	1.0
**KI81 220**	67.0	0.0	17.5	-	0.5	0.3	1.7	0.9	0.4	2.4
**KI81 220**	67.0	0.0	19.5	-	0.8	0.4	2.2	1.0	0.6	1.8
**KI81 220**	67.0	0.0	9.1	-	0.9	0.4	1.9	1.3	0.5	2.9

**KI81 220**	67.0	0.0	24.0	-	0.8	0.4	2.2	1.3	0.5	2.8
**KI81 220**	67.0	0.0	12.6	-	0.5	1.1	0.4	0.8	0.8	1.1
**KI81 220**	67.0	0.0	11.1	-	0.5	0.5	1.0	1.3	0.5	2.4
**KI81 220**	67.0	0.0	12.4	-	0.9	0.3	2.4	0.9	0.5	1.9
**KI81 220**	67.0	0.0	7.1	-	0.7	0.3	2.0	0.6	0.5	1.4
**KI81 220**	67.0	24.5	9.0	2.7	1.0	0.5	1.9	0.8	0.4	2.0
**KI81 220**	67.0	0.0	13.3	-	1.1	0.7	1.6	1.1	0.6	1.7
**KI81 220**	67.0	47.3	6.2	7.6	0.8	0.4	1.9	0.7	0.6	1.3
**KI81 220**	67.0	43.4	4.8	9.1	0.5	0.5	1.0	0.5	0.4	1.2
**KI81 220**	67.0	29.8	11.3	2.6	0.4	0.6	0.7	0.7	0.6	1.3
**KI81 220**	67.0	42.2	13.0	3.3	0.5	0.8	0.6	0.7	0.4	1.6
**KI81 220**	67.0	0.0	15.7	-	0.6	0.6	1.0	0.6	0.5	1.2
**KI81 220**	67.0	0.0	0.0	-	0.6	0.5	1.3	0.6	0.8	0.8
**KI81 220**	67.0	0.0	7.0	-	0.7	0.5	1.4	0.8	0.5	1.6
**KI81 220**	67.0	37.5	9.3	4.0	0.7	0.4	1.6	0.5	0.3	1.6
**KI81 220**	67.0	0.0	10.3	-	0.5	0.5	0.9	0.5	0.4	1.1
**KI81 220**	67.0	10.1	6.9	1.5	0.6	0.4	1.6	0.7	0.3	2.6
**KI81 220**	67.0	36.1	11.1	3.3	0.4	0.7	0.6	0.5	0.6	0.9
**KI81 220**	67.0	23.5	11.9	2.0	0.6	0.5	1.1	0.9	0.6	1.6
**KI81 220**	67.0	0.0	13.5	-	0.6	0.4	1.5	0.5	0.4	1.5
**KI81 220**	67.0	0.0	18.6	-	0.6	0.4	1.5	0.6	0.4	1.5
**KI81 220**	67.0	67.5	16.2	4.2	2.1	0.4	5.5	2.3	0.4	6.0
**KI81 220**	67.0	41.3	14.8	2.8	0.6	0.5	1.2	0.6	0.4	1.3
**KI81 220**	67.0	0.0	6.6	-	0.6	0.5	1.2	0.5	0.5	1.0
**KI81 220**	67.0	0.0	9.7	-	0.9	0.5	1.7	1.6	0.5	3.2
**KI81 220**	67.0	0.0	5.4	-	0.8	0.4	2.0	0.7	0.5	1.3
**KI81 220**	67.0	15.8	6.4	2.5	0.7	0.5	1.4	0.7	0.4	1.8
**KI81 220**	67.0	0.0	14.2	-	0.5	0.5	1.2	1.1	0.5	2.1
**KI81 220**	67.0	26.2	10.3	2.5	0.6	0.4	1.8	0.7	0.5	1.4
**KI81 220**	67.0	39.3	10.4	3.8	0.7	0.4	1.9	0.5	0.3	1.9
**KI81 220**	67.0	33.0	4.5	7.3	0.9	0.3	2.6	0.6	0.5	1.4
**KI81 220**	67.0	0.0	8.7	-	0.6	0.3	2.0	0.7	0.3	2.2
**KI81 220**	67.0	0.0	14.3	-	0.7	0.4	1.8	0.8	0.3	2.4
**KI81 220**	67.0	0.0	0.0	-	0.7	0.4	1.5	0.7	0.3	2.4
**KI81 220**	67.0	0.0	10.3	-	0.8	0.5	1.5	0.7	0.5	1.5
**KI81 220**	67.0	0.0	0.0	-	0.6	0.5	1.1	0.7	0.5	1.3
**KI81 220**	67.0	0.0	17.2	-	0.6	0.9	0.7	1.1	0.6	1.9
**KI81 220**	67.0	0.0	19.3	-	0.9	0.4	2.5	0.9	0.3	2.7
**KI81 220**	67.0	48.5	12.6	3.8	0.5	0.6	0.8	0.6	0.4	1.5
**KI81 220**	67.0	10.0	6.8	1.5	0.6	0.6	1.0	0.5	0.6	1.0
**KI81 220**	67.0	16.5	5.6	3.0	0.4	0.5	0.8	0.5	0.5	0.9
**KI81 220**	67.0	39.9	3.4	11.6	0.6	0.6	1.0	0.7	0.5	1.4
**KI81 220**	67.0	0.0	9.8	-	0.8	0.4	1.8	0.8	0.5	1.6
**KI81 220**	67.0	0.0	8.3	-	1.2	0.3	3.6	0.8	0.3	3.3

**KI81 220**	67.0	43.7	12.1	3.6	0.8	0.3	2.5	0.9	0.3	2.8
KI81 220 Average	*67.0*	*12.6*	*11.0*	*3.6*	*0.8*	*0.5*	*1.7*	*0.9*	*0.5*	*1.9*
**KI81 205**	62.6	0.0	32.9	-	0.5	0.3	1.5	0.5	0.3	1.4
**KI81 205**	62.6	0.0	11.6	-	0.6	0.4	1.5	0.6	0.5	1.1
**KI81 205**	62.6	81.9	38.0	2.2	1.0	0.3	3.3	0.9	0.3	3.1
**KI81 205**	62.6	0.0	0.0	-	0.4	0.5	0.9	0.4	0.4	1.2
**KI81 205**	62.6	0.0	0.0	-	0.4	0.3	1.3	0.5	0.4	1.3
**KI81 205**	62.6	100.2	35.4	2.8	0.6	0.2	2.6	0.8	0.3	2.9
**KI81 205**	62.6	0.0	0.0	-	0.5	0.4	1.4	0.5	0.5	0.9
**KI81 205**	62.6	58.7	0.0	-	0.5	0.3	1.6	0.7	0.4	1.6
**KI81 205**	62.6	79.5	39.0	2.0	0.4	0.4	1.2	0.4	0.4	1.0
**KI81 205**	62.6	0.0	34.9	-	0.6	0.6	1.0	0.5	0.4	1.1
**KI81 205**	62.6	0.0	0.0	-	0.6	0.2	2.8	1.0	0.4	2.3
**KI81 205**	62.6	0.0	52.8	-	0.4	0.3	1.6	0.5	0.4	1.1
**KI81 205**	62.6	0.0	0.0	-	0.8	0.3	2.6	0.9	0.3	3.1
**KI81 205**	62.6	0.0	19.2	-	0.5	0.3	1.8	0.6	0.3	2.1
**KI81 205**	62.6	0.0	30.2	-	0.6	0.2	2.9	0.6	0.2	2.7
**KI81 205**	62.6	0.0	31.8	-	1.5	0.5	2.8	0.5	0.5	1.1
**KI81 205**	62.6	0.0	19.6	-	0.4	0.3	1.4	0.5	0.3	1.8
**KI81 205**	62.6	0.0	9.8	-	0.8	0.3	2.5	0.8	0.3	2.7
**KI81 205**	62.6	0.0	0.0	-	0.8	0.3	3.2	0.5	0.3	1.9
**KI81 205**	62.6	0.0	29.0	-	0.6	0.3	2.2	0.6	0.3	1.8
**KI81 205**	62.6	0.0	7.5	-	0.5	0.3	1.8	0.9	0.3	2.9
**KI81 205**	62.6	0.0	9.5	-	0.5	0.2	2.7	0.5	0.3	2.0
**KI81 205**	62.6	0.0	36.0	-	0.5	0.3	1.5	0.7	0.3	2.4
**KI81 205**	62.6	0.0	35.2	-	0.6	0.3	2.1	0.5	0.2	2.0
**KI81 205**	62.6	0.0	7.6	-	0.5	0.2	2.9	0.4	0.3	1.4
**KI81 205**	62.6	0.0	17.6	-	0.4	0.3	1.1	0.4	0.4	0.9
**KI81 205**	62.6	0.0	15.9	-	0.3	0.3	1.2	0.4	0.2	2.3
**KI81 205**	62.6	0.0	16.2	-	0.3	0.2	1.8	0.3	0.2	1.6
**KI81 205**	62.6	0.0	9.5	-	0.4	0.2	2.7	0.4	0.1	2.5
**KI81 205**	62.6	0.0	8.1	-	0.3	0.2	1.5	0.2	0.2	1.6
**KI81 205**	62.6	0.0	11.0	-	0.8	0.3	2.4	0.5	0.3	2.0
**KI81 205**	62.6	0.0	11.9	-	0.4	0.3	1.3	0.4	0.3	1.2
**KI81 205**	62.6	0.0	17.2	-	0.4	0.3	1.3	0.4	0.2	1.6
**KI81 205**	62.6	0.0	10.8	-	0.3	0.3	1.2	0.3	0.3	1.0
**KI81 205**	62.6	0.0	10.9	-	0.4	0.3	1.4	0.3	0.2	1.3
**KI81 205**	62.6	0.0	9.7	-	0.2	0.3	0.8	0.5	0.3	1.8
**KI81 205**	62.6	0.0	15.4	-	0.7	0.4	1.8	0.5	0.3	1.6
**KI81 205**	62.6	0.0	25.2	-	0.3	0.3	1.0	0.3	0.3	1.0
**KI81 205**	62.6	0.0	14.7	-	0.5	0.2	2.9	0.3	0.2	1.8
**KI81 205**	62.6	0.0	13.1	-	0.4	0.3	1.2	0.4	0.2	2.3

**KI81 205**	62.6	0.0	26.2	-	0.5	0.2	2.4	0.5	0.3	1.5
KI81 205 Average	*62.6*	*7.8*	*17.4*	*2.3*	*0.5*	*0.3*	*1.9*	*0.5*	*0.3*	*1.8*
**KI81 193**	58.8	0.0	85.9	-	0.5	0.2	2.4	0.6	0.3	1.7
**KI81 193**	58.8	21.7	8.5	2.5	0.4	0.3	1.4	0.6	0.4	1.7
**KI81 193**	58.8	37.1	5.2	7.1	0.8	0.2	3.4	0.9	0.4	2.4
**KI81 193**	58.8	56.4	6.3	9.0	0.6	0.3	2.1	0.7	0.3	2.2
**KI81 193**	58.8	56.2	5.5	10.3	0.3	0.2	1.7	0.4	0.3	1.2
**KI81 193**	58.8	0.0	7.0	-	0.6	0.2	2.6	0.7	0.5	1.3
**KI81 193**	58.8	0.0	4.1	-	0.5	0.2	3.4	0.6	0.2	3.4
**KI81 193**	58.8	0.0	10.9	-	0.5	0.2	2.2	0.4	0.2	2.0
**KI81 193**	58.8	0.0	0.0	-	0.4	0.4	1.1	0.4	0.4	0.8
**KI81 193**	58.8	53.2	29.6	1.8	0.6	0.2	3.3	0.8	0.2	3.5
**KI81 193**	58.8	53.0	29.8	1.8	0.6	0.3	1.8	0.5	0.4	1.4
**KI81 193**	58.8	21.4	17.9	1.2	0.6	0.2	3.7	0.6	0.2	2.6
**KI81 193**	58.8	0.0	16.4	-	0.5	0.2	2.2	0.5	0.4	1.1
**KI81 193**	58.8	20.1	7.3	2.8	0.4	0.3	1.5	0.5	0.4	1.3
**KI81 193**	58.8	0.0	53.9	-	0.6	0.2	2.7	0.7	0.5	1.5
**KI81 193**	58.8	0.0	25.7	-	0.3	0.2	1.1	0.3	0.4	0.9
**KI81 193**	58.8	0.0	18.5	-	0.3	0.2	1.3	0.2	0.5	0.5
**KI81 193**	58.8	23.2	19.0	1.2	0.3	0.5	0.5	0.2	0.6	0.4
**KI81 193**	58.8	0.0	20.9	-	0.6	0.4	1.6	0.8	0.4	1.8
**KI81 193**	58.8	0.0	0.0	-	0.5	0.3	1.6	0.5	0.3	2.1
**KI81 193**	58.8	94.0	27.0	3.5	0.9	0.3	3.2	0.6	0.7	0.8
**KI81 193**	58.8	96.7	39.1	2.5	0.7	0.3	2.0	0.7	0.4	1.8
**KI81 193**	58.8	35.7	7.3	4.9	0.4	0.3	1.3	0.4	0.2	1.6
**KI81 193**	58.8	0.0	26.2	-	0.6	0.2	3.3	0.6	0.4	1.6
**KI81 193**	58.8	0.0	25.2	-	0.2	0.4	0.7	0.4	0.4	0.8
**KI81 193**	58.8	0.0	77.8	-	0.5	0.2	2.7	0.4	0.4	1.0
**KI81 193**	58.8	0.0	19.0	-	0.3	0.3	1.2	0.6	0.3	2.0
**KI81 193**	58.8	0.0	26.2	-	0.4	0.2	2.1	0.5	0.3	1.5
**KI81 193**	58.8	38.5	11.6	3.3	0.3	0.2	2.1	0.3	0.2	1.4
**KI81 193**	58.8	0.0	18.4	-	0.7	0.3	2.7	0.4	0.3	1.6
**KI81 193**	58.8	0.0	19.5	-	0.3	0.3	1.3	0.4	0.3	1.4
**KI81 193**	58.8	0.0	7.6	-	0.4	0.2	1.9	0.4	0.2	1.9
**KI81 193**	58.8	0.0	4.6	-	0.6	0.3	2.3	0.9	0.3	3.6
**KI81 193**	58.8	0.0	15.8	-	0.5	0.2	2.8	0.6	0.3	2.1
**KI81 193**	58.8	0.0	27.8	-	0.4	0.2	2.0	0.4	0.2	1.9
**KI81 193**	58.8	49.9	19.1	2.6	0.5	0.2	2.2	0.3	0.2	1.8
**KI81 193**	58.8	15.2	5.7	2.7	0.5	0.3	1.7	0.5	0.2	2.4
**KI81 193**	58.8	0.0	18.9	-	0.3	0.2	1.3	0.4	0.3	1.3
**KI81 193**	58.8	0.0	16.2	-	0.3	0.3	0.8	0.3	0.4	0.9
**KI81 193**	58.8	0.0	19.8	-	0.7	0.3	2.2	0.5	0.3	1.6

**KI81 193**	58.8	68.3	27.1	2.5	0.5	0.3	1.7	0.6	0.3	1.9
**KI81 193**	58.8	34.5	9.4	3.7	0.7	0.3	2.7	0.5	0.3	1.5
**KI81 193**	58.8	61.0	7.2	8.4	0.4	0.2	1.8	0.5	0.2	2.4
**KI81 193**	58.8	0.0	12.1	-	0.7	0.3	2.3	0.7	0.6	1.2
**KI81 193**	58.8	0.0	20.2	-	0.3	0.2	1.3	0.3	0.4	0.7
**KI81 193**	58.8	0.0	21.3	-	0.4	0.3	1.2	0.5	0.4	1.3
**KI81 193**	58.8	0.0	23.0	-	0.3	0.2	1.9	0.4	0.2	1.9
**KI81 193**	58.8	0.0	21.3	-	0.3	0.2	1.3	0.3	0.3	0.9
**KI81 193**	58.8	0.0	23.9	-	0.5	0.4	1.2	0.6	0.4	1.8
**KI81 193**	58.8	0.0	25.3	-	0.7	0.4	1.6	0.5	0.6	0.9
**KI81 193**	58.8	46.5	7.6	6.1	0.7	0.2	3.2	0.6	0.3	2.0
**KI81 193**	58.8	90.7	6.7	13.6	0.5	0.2	2.1	0.4	0.3	1.4
**KI81 193**	58.8	0.0	19.0	-	0.6	0.3	2.0	0.6	0.3	2.2
**KI81 193**	58.8	22.9	18.4	1.2	0.5	0.2	2.4	0.6	0.2	2.7
KI81 193 Average	*58.8*	*18.4*	*19.4*	*4.4*	*0.5*	*0.3*	*2.0*	*0.5*	*0.3*	*1.7*
**SRP 314**	95.9	59.4	15.3	3.9	1.3	0.6	2.0	1.0	-	-
**SRP 314**	95.9	64.2	14.7	4.4	1.2	1.6	0.7	1.9	-	-
**SRP 314**	95.9	40.1	15.2	2.6	1.4	1.2	1.2	0.5	-	-
**SRP 314**	95.9	40.1	15.2	2.6	1.8	1.6	1.1	0.7	-	-
**SRP 314**	95.9	21.5	7.5	2.9	1.7	0.6	2.8	1.6	0.4	4.3
**SRP 314**	95.9	21.5	7.5	2.9	2.9	1.0	2.9	1.4	0.2	5.8
**SRP 314**	95.9	23.4	6.0	3.9	1.7	1.1	1.6	0.6	0.2	2.8
**SRP 314**	95.9	23.4	6.0	3.9	1.7	1.3	1.3	0.6	0.2	2.7
**SRP 314**	95.9	12.3	4.8	2.6	1.5	0.7	2.3	1.2	-	-
**SRP 314**	95.9	12.3	4.8	2.6	1.9	1.1	1.7	1.3	0.3	3.9
**SRP 314**	95.9	53.3	7.5	7.1	2.2	1.1	2.1	4.5	0.7	6.9
**SRP 314**	95.9	23.1	4.0	5.8	1.2	1.1	1.1	0.8	0.7	1.1
**SRP 314**	95.9	23.0	7.5	3.1	1.2	1.2	1.1	0.8	0.4	2.1
**SRP 314**	95.9	59.7	6.7	8.9	1.8	0.8	2.1	1.6	0.3	4.5
**SRP 314**	95.9	28.6	7.8	3.7	1.5	0.9	1.6	2.0	0.5	4.4
**SRP 314**	95.9	34.9	4.5	7.7	1.2	1.1	1.1	0.3	0.4	0.6
**SRP 314**	95.9	51.4	17.7	2.9	3.4	0.9	3.7	2.3	0.4	6.2
**SRP 314**	95.9	51.4	17.7	2.9	1.1	0.9	1.2	2.5	0.4	6.7
**SRP 314**	95.9	43.8	8.3	5.2	2.5	0.9	2.7	1.2	0.6	2.0
**SRP 314**	95.9	87.6	6.8	12.9	3.6	1.0	3.6	0.8	0.4	2.1
**SRP 314**	95.9	130.0	8.1	16.0	1.2	1.2	0.9	1.5	0.5	2.9
**SRP 314**	95.9	25.1	3.1	8.0	1.8	1.1	1.6	1.1	0.4	2.6
**SRP 314**	95.9	100.6	10.8	9.3	1.3	0.6	2.0	1.9	0.6	3.1
**SRP 314**	95.9	100.2	13.2	7.6	2.9	1.4	2.1	1.3	0.6	2.3
**SRP 314**	95.9	41.5	6.1	6.8	1.3	1.3	1.0	3.0	0.8	3.7
**SRP 314**	95.9	105.6	11.4	9.3	2.0	1.0	2.0	2.9	0.7	4.2
**SRP 314**	95.9	105.6	11.4	9.3	2.0	1.2	1.7	2.9	0.5	6.0

**SRP 314**	95.9	48.6	11.2	4.3	4.6	1.6	2.9	1.7	0.6	2.9
**SRP 314**	95.9	16.7	12.7	1.3	1.5	0.8	1.9	2.0	0.2	8.0
**SRP 314**	95.9	24.4	6.4	3.8	1.1	1.0	1.2	2.1	0.4	5.8
**SRP 314**	95.9	14.1	4.1	3.5	1.5	0.7	2.2	0.3	-	-
**SRP 314**	95.9	96.3	25.9	3.7	1.3	0.4	3.1	1.3	0.4	3.5
**SRP 314**	95.9	29.4	16.5	1.8	1.8	1.5	1.2	0.7	0.5	1.4
**SRP 314**	95.9	172.5	20.4	8.5	1.9	1.1	1.8	2.1	0.2	9.0
**SRP 314**	95.9	33.6	11.9	2.8	1.0	2.0	0.5	1.3	0.3	4.1
**SRP 314**	95.9	43.8	13.7	3.2	4.1	0.7	5.6	2.4	0.3	9.3
**SRP 314**	95.9	55.9	9.0	6.2	1.7	0.7	2.2	2.6	0.5	4.7
**SRP 314**	95.9	167.7	9.9	16.9	1.4	0.9	1.6	2.6	0.1	29.6
**SRP 314**	95.9	44.0	5.7	7.7	1.4	2.0	0.7	3.0	0.4	8.1
**SRP 314**	95.9	13.1	6.1	2.1	1.0	1.3	0.8	0.7	0.3	2.4
**SRP 314**	95.9	52.7	7.2	7.3	2.0	1.0	2.1	3.8	0.6	6.7
**SRP 314**	95.9	85.5	21.0	4.1	2.2	1.3	1.7	1.9	0.4	4.3
**SRP 314**	95.9	83.4	6.3	13.3	1.3	1.1	1.2	1.3	0.5	2.5
**SRP 314**	95.9	37.6	10.3	3.7	1.9	1.1	1.7	1.9	0.3	7.3
**SRP 314**	95.9	67.8	15.1	4.5	2.3	0.7	3.2	2.2	0.6	3.9
**SRP 314**	95.9	35.7	4.4	8.1	1.3	1.0	1.3	2.1	0.2	9.0
**SRP 314**	95.9	61.4	9.5	6.5	2.5	0.7	3.8	3.9	0.4	9.9
**SRP 314**	95.9	38.1	7.5	5.1	1.1	1.3	0.8	1.4	0.6	2.6
**SRP 314**	95.9	28.8	4.7	6.1	3.1	0.7	4.8	2.2	0.2	9.5
**SRP 314**	95.9	90.6	7.3	12.4	2.0	1.4	1.4	2.1	0.5	4.6
**SRP 314**	95.9	19.4	7.2	2.7	1.7	1.0	1.6	1.6	0.4	4.0
**SRP 314**	95.9	59.5	6.4	9.2	1.6	0.8	1.9	1.4	0.3	4.2
**SRP 314**	95.9	39.3	10.7	3.7	2.2	0.9	2.5	5.1	0.3	14.5
**SRP 314**	95.9	113.5	8.9	12.7	2.3	0.3	7.9	1.6	0.3	5.9
**SRP 314**	95.9	71.2	12.8	5.6	1.1	1.1	1.0	1.1	0.6	1.8
**SRP 314**	95.9	37.4	10.6	3.5	1.0	1.0	1.1	1.2	0.3	3.5
**SRP 314**	95.9	51.1	10.3	4.9	2.8	0.7	4.0	3.2	0.3	10.0
**SRP 314**	95.9	63.6	9.7	6.5	2.7	0.9	3.0	1.5	0.4	3.9
**SRP 314**	95.9	26.4	6.8	3.9	1.6	1.0	1.6	0.4	0.5	0.9
**SRP 314**	95.9	53.0	8.2	6.5	1.4	1.3	1.1	1.8	0.4	4.2
**SRP 314**	95.9	50.0	6.9	7.3	2.5	1.0	2.5	2.1	0.3	6.7
**SRP 314**	95.9	71.6	18.3	3.9	2.0	1.7	1.2	1.3	0.6	2.1
**SRP 314**	95.9	25.5	11.7	2.2	1.9	1.1	1.8	3.5	1.0	3.6
**SRP 314**	95.9	35.6	10.9	3.3	2.3	0.8	2.9	2.8	0.5	5.1
**SRP 314**	95.9	35.6	10.9	3.3	2.3	1.1	2.0	2.8	0.2	14.4
**SRP 314**	95.9	24.3	7.1	3.4	4.1	1.7	2.4	1.0	0.6	1.8
**SRP 314**	95.9	39.0	5.5	7.1	1.5	0.5	2.9	0.3	0.4	0.8
**SRP 314**	95.9	62.6	7.5	8.4	3.1	1.3	2.4	2.7	0.4	7.5
**SRP 314**	95.9	97.9	10.7	9.2	3.5	0.8	4.6	3.1	2.4	1.3
**SRP 314**	95.9	100.7	13.1	7.7	2.8	0.9	2.9	2.6	0.4	7.1
**SRP 314**	95.9	103.0	11.3	9.1	2.1	1.6	1.3	0.2	0.5	0.5

**SRP 314**	95.9	19.2	5.7	3.4	1.6	1.6	1.0	2.1	0.5	3.9
**SRP 314**	95.9	49.1	9.7	5.1	2.4	1.0	2.4	5.3	0.3	19.3
**SRP 314**	95.9	49.1	9.7	5.1	2.4	1.8	1.4	5.3	0.4	15.0
**SRP 314**	95.9	33.7	11.2	3.0	3.0	1.5	2.0	2.0	0.5	4.0
**SRP 314**	95.9	134.4	12.6	10.7	3.4	2.8	1.2	3.5	0.4	8.4
**SRP 314**	95.9	54.5	16.1	3.4	2.1	1.3	1.6	3.3	0.3	11.7
**SRP 314**	95.9	54.5	16.1	3.4	2.1	1.4	1.5	3.3	0.6	5.3
**SRP 314**	95.9	102.1	27.1	3.8	4.8	2.0	2.4	1.9	1.4	1.4
SRP 314 Average	*95.9*	*55.7*	*10.3*	*5.8*	*2.0*	*1.1*	*2.1*	*2.0*	*0.5*	*5.5*
**SRP 301**	91.7	33.5	5.4	6.2	2.3	1.4	1.7	0.3	0.3	1.3
**SRP 301**	91.7	28.3	4.0	7.1	2.3	0.8	3.0	1.8	0.3	6.5
**SRP 301**	91.7	33.2	6.7	5.0	1.5	1.3	1.2	1.6	0.4	4.2
**SRP 301**	91.7	75.3	10.8	7.0	1.7	1.0	1.7	2.3	0.3	7.1
**SRP 301**	91.7	44.1	5.8	7.6	1.4	0.8	1.7	0.4	0.4	1.2
**SRP 301**	91.7	64.6	5.9	11.0	1.8	0.8	2.3	1.7	0.4	4.7
**SRP 301**	91.7	56.1	7.7	7.3	1.2	1.1	1.1	1.6	0.2	7.0
**SRP 301**	91.7	73.8	5.9	12.5	1.5	0.6	2.5	2.5	0.2	13.7
**SRP 301**	91.7	42.0	13.9	3.0	1.9	0.6	3.0	2.6	0.3	9.6
**SRP 301**	91.7	71.1	8.4	8.5	1.5	1.8	0.8	1.9	0.3	6.0
**SRP 301**	91.7	26.3	8.7	3.0	1.2	1.6	0.8	1.9	0.3	6.0
**SRP 301**	91.7	26.3	8.7	3.0	2.1	1.6	1.3	2.0	0.3	6.1
**SRP 301**	91.7	96.9	4.9	19.7	1.7	1.7	1.0	1.8	0.5	3.8
**SRP 301**	91.7	67.6	7.0	9.7	1.9	0.6	3.0	2.5	0.3	9.2
**SRP 301**	91.7	67.6	7.0	9.7	1.9	1.0	1.9	2.5	0.6	4.4
**SRP 301**	91.7	95.2	13.5	7.0	1.8	1.4	1.3	1.5	0.3	4.8
**SRP 301**	91.7	33.7	5.2	6.5	2.1	0.7	3.0	2.1	0.5	4.3
**SRP 301**	91.7	72.3	6.9	10.4	1.9	1.0	1.8	2.0	0.4	5.0
**SRP 301**	91.7	90.7	11.0	8.2	2.5	1.0	2.4	2.9	0.4	7.6
**SRP 301**	91.7	105.5	6.5	16.2	1.5	0.4	3.9	2.3	0.2	9.2
**SRP 301**	91.7	23.4	5.7	4.1	1.2	0.6	1.9	1.1	0.4	2.7
**SRP 301**	91.7	30.4	7.6	4.0	2.1	1.1	2.0	2.4	0.3	9.4
**SRP 301**	91.7	78.4	9.2	8.5	0.9	0.9	0.9	1.2	0.4	3.1
**SRP 301**	91.7	72.0	13.9	5.2	1.8	0.8	2.4	1.9	0.4	4.9
**SRP 301**	91.7	26.0	13.0	2.0	0.5	0.6	0.8	2.0	0.4	4.7
**SRP 301**	91.7	31.1	11.4	2.7	1.4	1.3	1.1	2.2	0.2	8.7
**SRP 301**	91.7	17.9	6.9	2.6	1.0	1.4	0.7	0.8	0.4	1.8
**SRP 301**	91.7	23.4	6.6	3.6	1.8	0.8	2.2	1.2	0.4	3.4
**SRP 301**	91.7	25.5	17.0	1.5	2.0	0.3	6.4	2.0	0.3	7.1
**SRP 301**	91.7	53.5	9.4	5.7	1.5	1.1	1.3	1.3	0.9	1.4
**SRP 301**	91.7	57.7	5.5	10.5	2.5	0.9	2.7	2.2	-	-
**SRP 301**	91.7	54.1	13.1	4.1	2.3	0.8	3.0	1.0	0.3	3.0
**SRP 301**	91.7	15.2	11.0	1.4	1.8	1.6	1.1	1.7	0.0	0.0

**SRP 301**	91.7	59.6	15.3	3.9	1.5	1.4	1.1	1.3	1.0	1.3
**SRP 301**	91.7	38.5	10.4	3.7	1.8	1.4	1.2	1.8	0.3	5.4
**SRP 301**	91.7	30.3	5.9	5.1	4.0	1.2	3.3	1.4	0.3	4.8
**SRP 301**	91.7	62.5	8.1	7.7	3.4	0.9	3.7	3.6	-	-
**SRP 301**	91.7	90.9	15.7	5.8	2.4	1.7	1.4	1.3	0.5	2.6
**SRP 301**	91.7	90.9	15.7	5.8	2.4	0.4	5.6	1.3	0.7	1.8
**SRP 301**	91.7	34.9	13.2	2.6	1.8	1.5	1.2	1.4	0.5	2.6
**SRP 301**	91.7	34.9	13.2	2.6	1.0	1.5	0.6	1.7	0.5	3.3
**SRP 301**	91.7	67.7	9.4	7.2	2.7	0.6	4.9	3.0	0.2	16.1
**SRP 301**	91.7	75.0	8.1	9.3	3.0	0.5	5.6	2.4	0.6	4.2
**SRP 301**	91.7	75.0	8.1	9.3	3.0	1.0	2.9	2.4	0.5	4.4
**SRP 301**	91.7	55.4	8.9	6.2	0.8	1.0	0.8	2.3	0.3	7.8
**SRP 301**	91.7	40.8	11.6	3.5	2.9	1.3	2.3	2.8	0.4	6.9
**SRP 301**	91.7	40.8	11.6	3.5	2.9	1.0	2.9	2.8	0.4	6.9
**SRP 301**	91.7	37.0	9.9	3.7	2.3	1.1	2.2	2.0	0.3	7.2
**SRP 301**	91.7	34.1	9.8	3.5	1.7	1.0	1.6	2.0	0.4	5.1
**SRP 301**	91.7	64.6	16.1	4.0	2.6	1.5	1.7	1.8	0.3	5.5
SRP 301 Average	*91.7*	*52.9*	*9.5*	*6.2*	*1.9*	*1.0*	*2.2*	*1.9*	*0.4*	*5.4*
**SRP 300**	91.5	105.2	7.7	13.7	2.4	0.2	13.7	2.8	-	-
**SRP 300**	91.5	41.9	5.0	8.4	2.0	1.9	1.1	1.7	0.3	5.1
**SRP 300**	91.5	76.4	9.3	8.2	4.4	1.2	3.7	3.4	0.5	6.7
**SRP 300**	91.5	23.3	13.0	1.8	1.9	0.7	2.8	2.3	0.5	4.9
**SRP 300**	91.5	23.3	13.0	1.8	1.4	0.7	2.1	-	0.5	-
**SRP 300**	91.5	19.6	4.8	4.1	1.0	0.9	1.1	1.0	0.2	5.6
**SRP 300**	91.5	19.6	4.8	4.1	0.9	0.9	0.9	0.3	0.2	1.4
**SRP 300**	91.5	42.4	5.2	8.1	1.2	0.5	2.4	1.1	0.5	2.2
**SRP 300**	91.5	24.9	4.7	5.3	1.0	0.9	1.1	2.7	0.3	8.3
**SRP 300**	91.5	20.8	4.6	4.6	1.4	0.6	2.4	1.5	0.3	4.7
**SRP 300**	91.5	29.4	7.2	4.1	1.2	0.8	1.5	1.2	0.2	6.1
**SRP 300**	91.5	40.3	5.1	8.0	1.2	0.8	1.4	1.4	0.5	2.8
**SRP 300**	91.5	17.0	4.6	3.7	1.3	0.7	1.8	2.7	0.2	11.3
**SRP 300**	91.5	71.6	9.0	8.0	0.8	1.1	0.7	2.1	0.3	8.0
**SRP 300**	91.5	38.0	5.2	7.4	2.2	1.0	2.1	1.6	0.3	4.7
**SRP 300**	91.5	38.0	5.2	7.4	1.3	1.0	1.3	1.9	0.3	5.9
**SRP 300**	91.5	36.8	5.1	7.2	1.1	0.7	1.6	0.7	0.3	2.8
**SRP 300**	91.5	36.8	5.1	7.2	1.4	0.7	2.2	1.8	0.3	6.8
**SRP 300**	91.5	56.5	4.7	11.9	1.0	0.5	1.8	2.2	0.5	4.7
**SRP 300**	91.5	56.5	4.7	11.9	1.0	0.5	2.0	2.2	0.3	6.8
**SRP 300**	91.5	51.4	4.7	10.8	1.2	0.5	2.2	1.2	0.5	2.4
**SRP 300**	91.5	57.3	5.6	10.2	1.5	0.9	1.7	1.7	0.4	4.0
**SRP 300**	91.5	56.0	8.5	6.6	1.6	0.5	3.3	0.3	0.3	1.1
**SRP 300**	91.5	35.5	4.0	8.8	1.3	0.5	2.7	1.3	0.2	6.4

**SRP 300**	91.5	34.9	5.6	6.3	1.0	0.5	1.8	0.5	0.0	0.0
**SRP 300**	91.5	19.5	6.7	2.9	1.4	0.6	2.1	1.7	0.3	5.7
**SRP 300**	91.5	14.9	7.9	1.9	1.4	0.8	1.8	1.6	0.4	4.3
**SRP 300**	91.5	39.3	7.6	5.2	1.3	0.5	2.5	0.8	-	-
**SRP 300**	91.5	39.3	7.6	5.2	1.3	1.1	1.2	0.8	0.3	2.6
**SRP 300**	91.5	50.4	3.4	15.0	1.1	0.6	2.0	0.7	0.1	4.6
**SRP 300**	91.5	20.8	3.2	6.4	1.0	0.8	1.3	0.7	0.4	2.1
**SRP 300**	91.5	15.9	5.9	2.7	1.1	1.0	1.1	0.8	0.4	2.2
**SRP 300**	91.5	102.2	6.2	16.6	1.2	0.5	2.2	2.1	0.3	6.5
**SRP 300**	91.5	29.1	8.7	3.4	2.0	0.4	4.6	2.3	0.0	0.0
**SRP 300**	91.5	29.1	8.7	3.4	1.7	1.3	1.3	1.1	0.4	2.6
**SRP 300**	91.5	26.1	6.1	4.2	1.3	0.6	2.1	0.8	0.9	0.9
**SRP 300**	91.5	36.7	9.4	3.9	0.8	0.7	1.2	1.2	0.3	4.1
**SRP 300**	91.5	36.7	9.4	3.9	1.2	0.7	1.7	1.4	0.3	4.5
**SRP 300**	91.5	27.7	5.3	5.2	1.0	0.7	1.4	0.4	0.3	1.2
**SRP 300**	91.5	27.7	5.3	5.2	1.0	0.7	1.4	0.4	0.0	-
**SRP 300**	91.5	33.7	4.8	7.0	0.7	0.9	0.8	0.8	0.3	2.7
**SRP 300**	91.5	47.9	11.6	4.1	2.2	0.9	2.4	1.9	0.4	4.8
**SRP 300**	91.5	36.3	5.7	6.4	1.2	0.8	1.5	1.6	-	-
**SRP 300**	91.5	62.7	12.1	5.2	2.1	0.5	4.3	2.1	0.4	6.0
**SRP 300**	91.5	47.3	4.7	10.0	1.7	1.1	1.6	1.2	0.3	4.3
**SRP 300**	91.5	56.3	5.2	10.8	1.2	0.8	1.5	1.6	0.2	6.9
**SRP 300**	91.5	60.8	5.1	11.9	1.8	0.5	3.9	1.6	0.3	5.1
**SRP 300**	91.5	42.3	13.8	3.1	1.5	0.6	2.4	1.6	0.4	3.7
**SRP 300**	91.5	42.3	13.8	3.1	2.0	0.4	5.4	1.8	0.7	2.6
**SRP 300**	91.5	32.7	8.8	3.7	0.5	0.7	0.8	0.4	0.5	0.9
**SRP 300**	91.5	85.6	7.4	11.5	1.3	0.8	1.6	1.3	0.4	3.6
**SRP 300**	91.5	20.2	6.4	3.1	1.7	0.9	2.0	1.4	0.2	7.1
**SRP 300**	91.5	20.2	6.4	3.1	0.7	0.9	0.8	0.5	0.2	2.6
**SRP 300**	91.5	12.9	5.3	2.4	0.8	0.9	0.9	1.2	0.0	0.0
**SRP 300**	91.5	81.2	6.6	12.3	1.2	1.0	1.2	1.2	0.3	4.3
**SRP 300**	91.5	22.1	7.2	3.1	0.9	0.8	1.3	1.0	0.3	2.9
**SRP 300**	91.5	48.1	5.9	8.1	0.9	0.4	2.1	1.1	-	-
**SRP 300**	91.5	36.2	8.0	4.5	1.3	0.8	1.5	0.3	0.2	1.4
**SRP 300**	91.5	36.2	8.0	4.5	1.3	0.5	2.4	0.3	0.0	0.0
**SRP 300**	91.5	35.0	8.3	4.2	1.1	0.9	1.3	1.1	0.2	5.3
**SRP 300**	91.5	43.2	10.8	4.0	1.4	1.0	1.4	0.9	0.3	2.8
**SRP 300**	91.5	27.3	10.5	2.6	1.2	0.6	1.9	1.0	-	-
**SRP 300**	91.5	25.7	3.8	6.8	1.5	0.7	2.2	1.3	-	-
**SRP 300**	91.5	18.8	5.4	3.5	0.9	0.8	1.1	1.0	-	-
**SRP 300**	91.5	36.1	6.0	6.0	1.0	0.6	1.6	1.2	0.5	2.5
**SRP 300**	91.5	24.3	13.6	1.8	2.1	1.6	1.3	1.8	0.4	4.8
**SRP 300**	91.5	24.3	13.6	1.8	1.6	1.6	1.0	1.8	0.4	4.8
**SRP 300**	91.5	43.1	8.4	5.1	1.5	0.5	3.2	1.3	-	-

**SRP 300**	91.5	43.1	8.4	5.1	1.5	1.0	1.4	1.3	0.2	6.2
**SRP 300**	91.5	30.1	12.8	2.3	1.3	0.7	1.9	1.1	0.2	5.3
**SRP 300**	91.5	30.1	12.8	2.3	1.3	1.4	0.9	1.1	0.3	3.2
**SRP 300**	91.5	28.6	7.8	3.7	1.3	0.8	1.6	1.2	0.3	3.8
**SRP 300**	91.5	29.6	5.4	5.5	1.2	0.7	1.7	1.3	0.2	6.1
**SRP 300**	91.5	64.7	7.2	9.0	1.5	0.6	2.5	1.2	0.3	4.8
**SRP 300**	91.5	48.5	7.1	6.9	2.3	0.5	4.3	1.7	0.4	4.0
**SRP 300**	91.5	48.5	7.1	6.9	2.3	0.5	4.3	1.7	0.2	8.2
**SRP 300**	91.5	42.4	6.1	7.0	1.3	1.0	1.3	0.9	-	-
**SRP 300**	91.5	46.8	6.8	6.9	1.4	1.1	1.3	1.2	0.2	6.9
**SRP 300**	91.5	27.9	4.0	7.0	1.3	0.6	2.3	1.6	0.6	2.8
**SRP 300**	91.5	21.4	11.7	1.8	1.0	0.8	1.3	1.1	0.3	3.3
**SRP 300**	91.5	10.8	6.7	1.6	2.2	0.6	3.7	2.2	0.0	0.0
**SRP 300**	91.5	10.8	6.7	1.6	1.9	0.6	3.0	1.2	0.3	3.9
**SRP 300**	91.5	28.3	6.0	4.7	1.3	0.8	1.7	1.7	0.3	6.1
**SRP 300**	91.5	28.3	6.0	4.7	1.3	0.9	1.4	1.7	0.3	4.9
**SRP 300**	91.5	99.8	15.0	6.6	1.2	0.6	1.8	1.4	0.5	3.2
**SRP 300**	91.5	87.5	9.8	8.9	1.3	1.1	1.2	1.3	0.4	3.4
**SRP 300**	91.5	78.1	5.8	13.5	1.3	0.4	3.7	1.4	0.4	3.3
**SRP 300**	91.5	77.0	5.4	14.2	1.3	0.6	2.4	1.6	0.3	6.1
**SRP 300**	91.5	78.1	5.8	13.5	0.9	0.5	1.7	1.3	0.6	2.1
**SRP 300**	91.5	42.5	3.7	11.4	1.0	0.4	2.7	1.1	0.3	4.0
**SRP 300**	91.5	62.7	14.1	4.4	0.7	0.6	1.1	2.0	0.5	4.0
**SRP 300**	91.5	32.2	4.8	6.7	2.3	0.9	2.6	3.1	0.5	6.6
**SRP 300**	91.5	48.3	13.9	3.5	3.0	1.0	3.0	3.0	0.6	5.3
**SRP 300**	91.5	48.3	13.9	3.5	2.2	1.0	2.2	2.0	0.6	3.5
**SRP 300**	91.5	52.3	11.0	4.8	1.2	0.4	2.8	1.6	0.4	3.9
**SRP 300**	91.5	52.3	11.0	4.8	1.2	0.6	2.0	1.6	0.3	6.1
**SRP 300**	91.5	34.1	3.9	8.7	1.3	0.5	2.3	1.3	0.2	6.4
SRP 300 Average	*91.5*	*41.3*	*7.5*	*6.2*	*1.4*	*0.8*	*2.1*	*1.4*	*0.3*	*4.2*
**SRP 299**	91.3	24.1	7.8	3.1	2.4	1.2	2.0	0.0	0.0	-
**SRP 299**	91.3	20.8	7.5	2.8	1.6	0.7	2.3	0.4	0.6	0.7
**SRP 299**	91.3	20.8	7.5	2.8	1.6	0.7	2.3	1.3	0.6	2.2
**SRP 299**	91.3	25.1	7.1	3.5	0.5	0.8	0.6	1.3	0.3	4.7
**SRP 299**	91.3	25.1	7.1	3.5	1.2	0.8	1.6	1.2	0.3	4.4
**SRP 299**	91.3	14.8	3.2	4.7	0.5	0.4	1.2	1.0	0.1	7.3
**SRP 299**	91.3	15.5	6.5	2.4	0.7	0.7	1.1	0.9	0.7	1.4
**SRP 299**	91.3	15.5	6.5	2.4	0.7	0.6	1.4	0.9	0.4	2.1
**SRP 299**	91.3	29.4	2.4	12.2	1.0	0.3	3.6	0.2	0.5	0.4
**SRP 299**	91.3	14.9	7.4	2.0	0.6	0.8	0.8	0.5	0.3	1.4
**SRP 299**	91.3	7.0	3.5	2.0	0.7	0.6	1.3	0.8	0.5	1.7
**SRP 299**	91.3	53.6	7.0	7.6	1.0	0.7	1.5	1.5	0.5	3.3

**SRP 299**	91.3	129.8	30.1	4.3	0.6	0.6	1.0	0.8	-	-
**SRP 299**	91.3	31.6	14.5	2.2	1.0	0.5	2.1	1.2	0.2	5.1
**SRP 299**	91.3	31.6	14.5	2.2	1.0	0.6	1.8	1.2	0.4	2.8
**SRP 299**	91.3	33.7	6.3	5.3	1.0	1.1	0.9	1.1	0.2	5.8
**SRP 299**	91.3	33.7	6.3	5.3	1.0	0.8	1.3	1.1	-	-
**SRP 299**	91.3	121.4	9.2	13.1	1.7	0.6	2.7	1.4	0.4	3.3
**SRP 299**	91.3	112.5	11.8	9.5	0.9	0.5	1.7	1.1	0.4	2.9
**SRP 299**	91.3	63.1	11.8	5.3	0.8	1.0	0.8	0.7	0.3	2.4
**SRP 299**	91.3	79.2	10.1	7.8	1.0	0.8	1.3	0.7	0.3	2.1
**SRP 299**	91.3	79.2	10.1	7.8	1.0	0.7	1.5	0.7	-	-
**SRP 299**	91.3	23.7	8.9	2.7	1.1	1.6	0.7	0.2	0.3	0.7
**SRP 299**	91.3	23.7	8.9	2.7	1.9	1.6	1.2	0.7	0.3	2.4
**SRP 299**	91.3	18.6	7.8	2.4	1.7	0.6	2.8	1.5	0.3	4.4
**SRP 299**	91.3	18.6	7.8	2.4	0.8	0.6	1.4	0.9	0.3	2.8
**SRP 299**	91.3	19.8	13.6	1.5	1.2	0.7	1.8	1.3	0.5	2.8
**SRP 299**	91.3	19.8	13.6	1.5	0.7	0.4	1.7	0.6	0.2	2.7
**SRP 299**	91.3	31.3	5.2	6.0	1.4	0.7	1.8	0.4	0.5	0.7
**SRP 299**	91.3	31.3	5.2	6.0	1.4	0.6	2.4	0.4	0.3	1.2
**SRP 299**	91.3	65.9	8.5	7.7	1.2	0.4	3.5	1.5	0.3	4.6
**SRP 299**	91.3	65.9	8.5	7.7	1.2	0.7	1.8	1.5	0.4	3.7
**SRP 299**	91.3	15.4	4.7	3.3	1.0	0.5	1.8	0.9	0.3	2.9
**SRP 299**	91.3	14.2	3.8	3.8	0.7	0.6	1.1	1.1	0.3	3.7
**SRP 299**	91.3	71.0	9.3	7.7	2.2	0.6	3.6	1.5	0.5	2.9
**SRP 299**	91.3	71.0	9.3	7.7	2.2	0.8	2.8	1.5	0.5	3.1
**SRP 299**	91.3	104.3	5.7	18.2	1.0	0.6	1.8	1.3	0.2	5.8
**SRP 299**	91.3	14.8	4.1	3.6	1.6	0.5	3.4	0.7	0.3	2.7
**SRP 299**	91.3	26.2	4.6	5.6	1.3	0.3	4.3	1.2	0.3	4.0
**SRP 299**	91.3	49.1	4.6	10.7	1.3	0.5	2.4	0.4	0.5	0.8
**SRP 299**	91.3	98.8	6.6	15.1	1.4	0.4	3.9	1.3	0.4	3.4
**SRP 299**	91.3	19.9	5.0	4.0	0.8	0.5	1.6	0.5	0.5	1.1
**SRP 299**	91.3	19.9	5.0	4.0	0.8	0.5	1.8	0.5	0.2	2.4
**SRP 299**	91.3	60.9	4.8	12.8	1.1	0.4	3.1	0.8	0.2	3.1
**SRP 299**	91.3	60.9	4.8	12.8	1.1	0.2	4.9	0.8	0.5	1.5
**SRP 299**	91.3	52.7	5.2	10.2	0.9	0.4	2.2	1.0	0.3	3.7
**SRP 299**	91.3	35.2	6.4	5.5	1.3	0.3	4.5	1.0	0.3	3.6
**SRP 299**	91.3	35.2	6.4	5.5	1.3	0.4	3.0	1.0	0.2	5.2
**SRP 299**	91.3	52.7	5.2	10.2	1.2	0.8	1.5	1.3	0.2	6.1
**SRP 299**	91.3	16.7	2.9	5.7	1.9	0.6	3.5	1.6	0.3	5.9
SRP 299 Average	*91.3*	*42.4*	*7.7*	*6.0*	*1.2*	*0.6*	*2.1*	*0.9*	*0.4*	*3.1*
**SRP 298**	90.8	52.9	15.4	3.4	1.3	0.5	2.6	2.8	0.3	10.6
**SRP 298**	90.8	53.3	5.3	10.0	1.7	0.8	2.0	2.3	-	-
**SRP 298**	90.8	54.0	4.8	11.1	1.7	0.8	2.0	1.3	0.2	6.3

**SRP 298**	90.8	80.7	8.1	10.0	1.8	0.6	2.9	2.8	-	-
**SRP 298**	90.8	31.1	8.7	3.6	0.6	0.5	1.0	0.4	-	-
**SRP 298**	90.8	56.7	20.9	2.7	1.8	1.6	1.1	1.6	-	-
**SRP 298**	90.8	22.5	6.3	3.6	0.7	0.4	1.8	0.6	0.3	1.9
**SRP 298**	90.8	44.4	5.7	7.8	1.0	0.8	1.3	2.9	-	-
**SRP 298**	90.8	35.2	6.6	5.4	0.6	0.9	0.6	3.3	0.3	9.8
**SRP 298**	90.8	40.1	4.7	8.5	0.9	1.2	0.7	2.5	0.0	0.0
**SRP 298**	90.8	17.2	3.2	5.3	0.9	1.0	0.8	1.3	0.3	3.9
**SRP 298**	90.8	22.2	4.2	5.2	0.8	0.6	1.2	1.2	-	-
**SRP 298**	90.8	22.2	4.2	5.2	0.8	0.4	2.0	1.2	0.3	4.5
**SRP 298**	90.8	29.4	11.1	2.6	1.5	1.3	1.1	1.8	0.0	0.0
**SRP 298**	90.8	58.1	9.5	6.1	0.8	1.0	0.8	1.1	0.5	2.2
**SRP 298**	90.8	43.6	6.8	6.4	1.5	1.0	1.5	2.0	0.5	4.1
**SRP 298**	90.8	63.6	7.8	8.1	1.4	0.7	2.0	3.2	0.2	21.1
**SRP 298**	90.8	59.1	12.0	4.9	1.6	0.9	1.7	4.4	0.3	14.8
**SRP 298**	90.8	48.5	8.7	5.6	1.4	1.1	1.3	1.9	-	-
**SRP 298**	90.8	20.5	7.3	2.8	1.6	0.7	2.2	1.5	-	-
**SRP 298**	90.8	28.4	14.9	1.9	1.6	0.7	2.4	1.6	0.4	4.1
**SRP 298**	90.8	76.1	7.8	9.8	1.8	0.3	5.1	3.7	0.4	8.4
**SRP 298**	90.8	142.8	7.6	18.8	1.6	0.8	1.9	2.0	0.5	3.9
**SRP 298**	90.8	22.2	5.4	4.1	1.3	0.3	4.2	0.5	-	-
**SRP 298**	90.8	71.8	7.9	9.1	0.7	0.6	1.3	0.6	-	-
**SRP 298**	90.8	71.8	7.9	9.1	0.7	0.5	1.5	0.6	-	-
**SRP 298**	90.8	27.0	4.3	6.2	1.5	0.6	2.5	0.0	-	-
**SRP 298**	90.8	27.0	4.3	6.2	1.5	0.3	5.4	0.0	-	-
**SRP 298**	90.8	71.8	7.9	9.1	0.5	0.7	0.7	1.0	0.5	2.0
**SRP 298**	90.8	71.8	7.9	9.1	0.5	1.0	0.5	1.0	0.3	3.2
**SRP 298**	90.8	88.7	5.7	15.6	0.7	1.4	0.5	0.4	0.3	1.4
**SRP 298**	90.8	44.7	17.8	2.5	1.2	1.3	0.9	2.2	0.0	-
**SRP 298**	90.8	55.0	5.9	9.3	0.8	0.8	1.1	0.9	0.6	1.4
**SRP 298**	90.8	20.9	4.3	4.9	1.6	0.4	4.2	0.4	-	-
**SRP 298**	90.8	23.9	4.5	5.3	0.5	0.9	0.6	0.7	-	-
**SRP 298**	90.8	23.9	4.5	5.3	1.4	0.9	1.5	2.6	-	-
**SRP 298**	90.8	24.3	4.8	5.1	0.9	0.6	1.4	1.0	-	-
**SRP 298**	90.8	29.7	5.1	5.8	1.2	0.6	2.1	1.7	0.3	5.2
**SRP 298**	90.8	29.7	5.1	5.8	1.2	1.0	1.2	1.7	0.6	2.9
**SRP 298**	90.8	38.7	9.7	4.0	1.7	0.9	1.8	1.4	-	-
**SRP 298**	90.8	38.7	9.7	4.0	1.7	0.4	3.9	1.4	-	-
**SRP 298**	90.8	43.9	9.8	4.5	1.8	0.4	4.5	1.5	0.3	4.9
**SRP 298**	90.8	65.7	6.1	10.7	2.1	0.7	3.2	2.3	0.5	4.7
**SRP 298**	90.8	64.1	5.3	12.2	1.8	1.3	1.3	1.8	0.2	7.9
**SRP 298**	90.8	71.6	8.0	9.0	1.2	1.4	0.9	1.5	0.7	2.3
**SRP 298**	90.8	43.6	6.5	6.7	1.5	0.5	2.7	1.9	0.3	6.3
**SRP 298**	90.8	51.0	12.2	4.2	2.3	0.3	6.9	2.0	0.5	3.7

**SRP 298**	90.8	42.3	7.0	6.1	1.3	0.6	2.2	2.1	0.7	2.9
**SRP 298**	90.8	61.2	4.1	14.8	1.2	1.1	1.1	1.8	0.4	4.6
**SRP 298**	90.8	61.2	4.1	14.8	1.2	0.4	3.3	1.8	0.3	5.2
**SRP 298**	90.8	31.9	6.0	5.3	1.8	0.7	2.6	1.5	0.3	5.1
**SRP 298**	90.8	60.1	9.0	6.6	1.0	0.6	1.7	2.2	0.3	6.8
**SRP 298**	90.8	91.2	6.4	14.3	1.3	0.7	1.8	2.7	0.2	12.1
**SRP 298**	90.8	82.1	13.8	5.9	0.8	0.8	1.0	2.3	0.7	3.5
**SRP 298**	90.8	40.0	5.2	7.7	0.6	1.1	0.6	1.3	0.2	8.1
**SRP 298**	90.8	23.2	4.4	5.3	0.9	0.3	3.6	1.8	0.5	3.8
**SRP 298**	90.8	23.2	4.4	5.3	0.9	0.2	4.0	1.8	0.2	7.1
**SRP 298**	90.8	40.3	6.0	6.8	1.3	1.1	1.1	2.6	0.2	11.4
**SRP 298**	90.8	104.5	4.5	23.3	0.9	0.3	3.4	1.8	0.2	7.6
**SRP 298**	90.8	26.3	5.9	4.5	0.7	0.4	2.0	0.8	0.3	2.9
**SRP 298**	90.8	48.7	21.5	2.3	1.9	1.5	1.3	3.4	2.1	1.6
**SRP 298**	90.8	35.6	11.4	3.1	1.4	1.3	1.1	2.6	0.5	5.1
**SRP 298**	90.8	68.8	12.7	5.4	1.8	0.5	3.3	2.0	0.4	5.4
**SRP 298**	90.8	28.9	15.0	1.9	0.6	1.3	0.5	1.4	0.6	2.5
**SRP 298**	90.8	28.9	15.0	1.9	0.6	0.5	1.3	1.4	0.3	4.3
**SRP 298**	90.8	23.3	11.3	2.1	1.3	0.9	1.5	2.1	0.7	2.9
**SRP 298**	90.8	53.3	10.5	5.1	1.6	0.5	3.1	3.6	0.2	16.2
**SRP 298**	90.8	60.0	10.9	5.5	1.4	0.5	2.5	1.5	0.4	4.2
**SRP 298**	90.8	154.7	43.8	3.5	0.5	0.5	1.0	0.5	0.7	0.7
**SRP 298**	90.8	67.3	3.7	18.4	1.4	0.4	3.9	1.6	0.2	7.0
**SRP 298**	90.8	40.8	9.6	4.2	1.3	1.1	1.2	1.2	0.7	1.6
**SRP 298**	90.8	45.5	7.8	5.4	1.5	0.7	2.0	1.8	0.3	6.3
**SRP 298**	90.8	50.1	8.8	6.6	1.5	0.8	2.4	1.8	0.3	6.0
SRP 298 Average	*90.8*	*49.6*	*8.6*	*6.9*	*1.2*	*0.8*	*2.0*	*1.7*	*0.4*	*5.4*
**LAKI 04**	-	80.3	23.2	3.5	2.0	0.2	10.9	-	-	-
**LAKI 04**	-	125.1	24.5	5.1	0.7	0.4	1.7	-	-	-
**LAKI 04**	-	24.7	6.5	3.8	0.9	0.5	1.7	-	-	-
**LAKI 04**	-	28.9	6.6	4.4	0.3	0.2	1.1	-	-	-
**LAKI 04**	-	96.2	12.0	8.0	0.7	0.3	2.4	-	-	-
**LAKI 04**	-	144.8	17.9	8.1	1.0	0.3	3.3	-	-	-
**LAKI 04**	-	144.8	17.9	8.1	1.0	0.2	4.6	-	-	-
**LAKI 04**	-	32.9	9.8	3.4	0.3	0.3	1.3	-	-	-
**LAKI 04**	-	25.7	10.6	2.4	0.7	0.3	2.3	-	-	-
**LAKI 04**	-	25.7	10.6	2.4	0.7	0.2	4.3	-	-	-
**LAKI 04**	-	40.3	16.4	2.5	0.8	0.3	2.6	-	-	-
**LAKI 04**	-	80.7	13.6	5.9	1.4	0.3	4.3	-	-	-
**LAKI 04**	-	124.2	10.2	12.2	0.5	0.3	1.8	-	-	-
**LAKI 04**	-	140.0	29.8	4.7	0.7	0.3	2.7	-	-	-
**LAKI 04**	-	140.0	29.8	4.7	0.7	0.3	2.3	-	-	-

**LAKI 04**	-	12.7	5.8	2.2	0.8	0.3	2.5	-	-	-
**LAKI 04**	-	64.7	10.9	5.9	1.0	0.3	3.6	-	-	-
**LAKI 04**	-	64.7	10.9	5.9	1.0	0.3	3.4	-	-	-
**LAKI 04**	-	74.4	6.4	11.7	0.4	0.2	1.6	-	-	-
**LAKI 04**	-	80.9	13.2	6.1	0.7	0.1	5.6	-	-	-
**LAKI 04**	-	29.8	13.8	2.2	1.0	0.4	2.5	-	-	-
**LAKI 04**	-	29.8	13.8	2.2	1.0	0.4	2.4	-	-	-
**LAKI 04**	-	1.4	0.1	9.7	0.7	0.2	3.2	-	-	-
**LAKI 04**	-	59.8	6.5	9.1	1.1	0.3	4.0	-	-	-
**LAKI 04**	-	113.4	5.0	22.7	1.0	0.6	1.7	-	-	-
**LAKI 04**	-	116.5	4.3	27.2	0.6	0.2	3.1	-	-	-
**LAKI 04**	-	87.3	26.7	3.3	0.7	0.3	2.4	-	-	-
**LAKI 04**	-	102.0	22.4	4.6	0.7	0.3	2.3	-	-	-
**LAKI 04**	-	42.7	6.0	7.1	0.3	0.1	2.4	-	-	-
**LAKI 04**	-	101.6	7.7	13.2	0.6	0.2	3.2	-	-	-
**LAKI 04**	-	111.8	8.3	13.4	0.7	0.2	2.7	-	-	-
**LAKI 04**	-	160.3	15.0	10.7	0.7	0.2	3.1	-	-	-
**LAKI 04**	-	158.9	15.0	10.6	0.6	0.2	3.6	-	-	-
**LAKI 04**	-	30.1	5.6	5.4	0.5	0.3	1.5	-	-	-
**LAKI 04**	-	42.9	9.6	4.4	0.6	0.5	1.2	-	-	-
**LAKI 04**	-	36.9	6.9	5.4	0.5	0.2	2.1	-	-	-
**LAKI 04**	-	81.1	5.8	14.1	0.4	0.2	2.1	-	-	-
**LAKI 04**	-	84.5	10.0	8.5	0.5	0.2	2.2	-	-	-
**LAKI 04**	-	83.8	21.5	3.9	0.4	0.6	0.6	-	-	-
**LAKI 04**	-	134.0	12.1	11.1	0.7	0.2	2.8	-	-	-
**LAKI 04**	-	129.4	10.1	12.8	0.4	0.2	1.9	-	-	-
**LAKI 04**	-	33.4	4.9	6.8	0.5	0.2	2.1	-	-	-
**LAKI 04**	-	29.5	7.1	4.1	0.6	0.2	2.5	-	-	-
**LAKI 04**	-	13.9	8.7	1.6	1.2	0.2	5.1	-	-	-
**LAKI 04**	-	17.1	6.1	2.8	0.8	0.3	3.1	-	-	-
**LAKI 04**	-	17.1	6.1	2.8	0.8	0.2	3.8	-	-	-
**LAKI 04**	-	49.2	7.0	7.1	1.0	0.2	4.9	-	-	-
**LAKI 04**	-	105.3	2.8	38.1	1.0	0.1	7.4	-	-	-
**LAKI 04**	-	121.2	5.3	23.0	1.0	0.2	5.1	-	-	-
**LAKI 04**	-	121.2	5.3	23.0	1.0	0.2	4.3	-	-	-
**LAKI 04**	-	30.0	12.6	2.4	1.0	0.3	3.7	-	-	-
**LAKI 04**	-	30.0	12.6	2.4	0.8	0.2	4.0	-	-	-
**LAKI 04**	-	40.1	5.3	7.6	0.7	0.3	2.0	-	-	-
**LAKI 04**	-	11.2	3.5	3.2	0.7	0.2	3.7	-	-	-
**LAKI 04**	-	31.8	6.6	4.8	0.7	0.3	2.2	-	-	-
**LAKI 04**	-	31.8	6.6	4.8	0.6	0.5	1.2	-	-	-
**LAKI 04**	-	49.8	4.5	11.0	0.5	0.3	1.8	-	-	-
**LAKI 04**	-	33.8	5.3	6.4	0.4	0.2	2.4	-	-	-
**LAKI 04**	-	34.2	4.9	7.0	0.7	0.2	3.8	-	-	-

**LAKI 04**	-	42.7	5.6	7.6	0.9	0.3	3.3	-	-	-
**LAKI 04**	-	42.7	5.6	7.6	0.7	0.3	2.6	-	-	-
**LAKI 04**	-	21.8	3.7	5.9	0.8	0.2	3.5	-	-	-
**LAKI 04**	-	49.8	4.5	11.0	0.3	0.1	2.5	-	-	-
**LAKI 04**	-	33.8	5.3	6.4	0.5	0.2	3.3	-	-	-
**LAKI 04**	-	56.5	16.3	3.5	0.9	0.3	2.6	-	-	-
**LAKI 04**	-	56.5	16.3	3.5	0.9	0.3	3.2	-	-	-
**LAKI 04**	-	54.1	11.2	4.8	0.7	0.3	2.1	-	-	-
LAKI 04 Average	-	*66.4*	*10.4*	*7.8*	*0.7*	*0.3*	*3.0*	-	-	*0.0*

Table 3 Line profiles of composition (obtained using EPMA), extending up to 50 µm outwards from the Fe-rich CBL around plagioclase, in samples from 44.8 m (KI 147) depth and 45.4 m (KI 149) depth

(corresponding to quench temperatures of 1090°C and 1112°C, respectively).

Sample	Transect no.	Location relative to (010) plag face	SiO_2	SD	TiO_2	SD	Al_2O_3	SD	FeO	SD	MnO	SD	MgO	SD	CaO	SD	Na_2O	SD	K_2O	SD	P_2O_5	SD	Total	Distance (µm)
**KI 149**	1	Perp to (010)	48.88	0.65	5.22	0.08	11.94	0.35	11.93	0.45	0.20	0.04	4.52	0.20	7.71	0.18	2.60	0.14	1.14	0.08	0.78	0.04	94.91	0.0
**KI 149**	1	Perp to (010)	48.86	0.65	5.14	0.10	11.85	0.35	12.02	0.45	0.21	0.04	4.37	0.20	7.83	0.19	2.46	0.14	1.24	0.08	0.79	0.03	94.77	2.1
**KI 149**	1	Perp to (010)	49.21	0.65	5.24	0.10	12.04	0.35	12.07	0.45	0.19	0.04	4.37	0.20	7.75	0.18	2.28	0.13	1.21	0.08	0.79	0.04	95.12	4.3
**KI 149**	1	Perp to (010)	50.17	0.66	5.33	0.10	12.17	0.35	12.28	0.46	0.17	0.04	4.57	0.21	7.82	0.19	2.13	0.13	1.14	0.08	0.80	0.04	96.59	6.4
**KI 149**	1	Perp to (010)	49.89	0.66	5.29	0.10	12.43	0.35	12.21	0.45	0.19	0.04	4.53	0.21	7.98	0.19	2.03	0.12	1.22	0.08	0.80	0.04	96.57	8.6
**KI 149**	1	Perp to (010)	50.35	0.66	5.30	0.10	12.12	0.35	12.46	0.46	0.17	0.05	4.52	0.21	7.90	0.19	2.02	0.12	1.18	0.08	0.80	0.04	96.81	10.7
**KI 149**	1	Perp to (010)	50.22	0.66	5.26	0.10	12.37	0.35	12.54	0.46	0.18	0.05	4.56	0.21	7.93	0.19	2.04	0.12	1.20	0.08	0.78	0.04	97.07	12.9
**KI 149**	1	Perp to (010)	49.95	0.66	5.33	0.10	12.03	0.35	12.32	0.46	0.18	0.04	4.62	0.21	8.02	0.19	2.01	0.12	1.20	0.08	0.80	0.04	96.47	15.0
**KI 149**	1	Perp to (010)	49.94	0.66	5.35	0.10	12.03	0.35	12.60	0.46	0.17	0.04	4.55	0.21	7.93	0.19	1.99	0.12	1.22	0.08	0.80	0.04	96.58	17.2
**KI 149**	1	Perp to (010)	49.86	0.66	5.32	0.10	12.04	0.35	12.43	0.46	0.16	0.04	4.58	0.21	7.86	0.19	1.97	0.12	1.18	0.08	0.77	0.04	96.18	19.3
**KI 149**	1	Perp to (010)	49.92	0.66	5.34	0.10	11.95	0.35	12.30	0.46	0.20	0.04	4.55	0.21	7.84	0.19	1.92	0.12	1.26	0.08	0.82	0.04	96.10	21.4
**KI 149**	1	Perp to (010)	50.23	0.66	5.06	0.10	12.96	0.36	11.77	0.45	0.17	0.04	4.36	0.20	8.05	0.19	2.10	0.13	1.11	0.08	0.78	0.04	96.57	23.6
**KI 149**	1	Perp to (010)	50.27	0.66	4.60	0.10	14.90	0.39	10.79	0.42	0.16	0.04	3.84	0.19	8.58	0.19	2.20	0.13	1.01	0.07	0.71	0.03	97.06	25.7
**KI 149**	1	Perp to (010)	49.92	0.66	3.94	0.10	17.31	0.41	9.00	0.39	0.16	0.04	3.24	0.17	9.71	0.21	2.24	0.13	0.88	0.07	0.64	0.03	97.04	27.9
**KI 149**	1	Perp to (010)	49.89	0.66	3.31	0.10	19.71	0.44	7.77	0.36	0.14	0.04	2.69	0.16	10.39	0.21	2.51	0.14	0.77	0.07	0.59	0.03	97.77	30.0
**KI 149**	2	Parallel to (010)	50.31	0.66	4.02	0.09	17.20	0.41	8.71	0.38	0.11	0.04	2.98	0.17	9.60	0.21	3.59	0.17	0.68	0.06	0.66	0.03	97.87	0.0
**KI 149**	2	Parallel to (010)	49.34	0.66	5.26	0.09	13.37	0.37	11.53	0.44	0.15	0.04	4.21	0.20	8.62	0.19	3.00	0.15	0.95	0.07	0.83	0.04	97.27	2.1
**KI 149**	2	Parallel to (010)	49.20	0.65	5.79	0.10	11.45	0.34	12.83	0.47	0.20	0.04	4.86	0.21	8.33	0.19	2.61	0.14	1.04	0.08	0.88	0.04	97.20	4.1
**KI 149**	2	Parallel to (010)	48.71	0.65	6.11	0.10	10.01	0.32	13.56	0.48	0.18	0.04	5.06	0.22	8.34	0.19	2.16	0.13	1.18	0.08	0.92	0.04	96.23	6.2
**KI 149**	2	Parallel to (010)	49.23	0.65	6.02	0.10	10.20	0.32	13.38	0.48	0.21	0.05	5.08	0.22	8.11	0.19	1.99	0.12	1.21	0.08	0.92	0.04	96.36	8.3
**KI 149**	2	Parallel to (010)	49.97	0.66	5.67	0.10	10.78	0.33	12.62	0.46	0.20	0.04	4.94	0.21	7.95	0.19	1.94	0.12	1.24	0.08	0.87	0.04	96.19	10.4
**KI 149**	2	Parallel to (010)	50.25	0.66	5.41	0.10	11.21	0.34	12.48	0.46	0.20	0.04	4.73	0.21	7.74	0.18	1.96	0.12	1.33	0.09	0.83	0.04	96.14	12.4
**KI 149**	2	Parallel to (010)	50.19	0.66	5.37	0.10	11.58	0.34	12.32	0.46	0.24	0.04	4.62	0.21	7.76	0.18	1.98	0.12	1.25	0.08	0.80	0.04	96.11	14.5
**KI 149**	2	Parallel to (010)	49.97	0.66	5.27	0.10	11.84	0.35	12.74	0.47	0.19	0.04	4.48	0.20	7.87	0.19	1.95	0.12	1.21	0.08	0.78	0.04	96.29	16.6
**KI 149**	2	Parallel to (010)	49.66	0.66	5.23	0.10	11.88	0.35	12.61	0.46	0.19	0.05	4.54	0.21	7.92	0.19	2.05	0.13	1.19	0.08	0.78	0.04	96.05	18.6
**KI 149**	2	Parallel to (010)	50.03	0.66	5.31	0.10	12.16	0.35	12.51	0.46	0.21	0.04	4.66	0.21	7.82	0.19	1.94	0.12	1.24	0.08	0.83	0.04	96.71	20.7
**KI 149**	2	Parallel to (010)	50.11	0.66	5.31	0.09	12.27	0.35	12.43	0.46	0.19	0.04	4.49	0.20	7.86	0.19	1.90	0.12	1.19	0.08	0.80	0.04	96.58	22.8
**KI 149**	2	Parallel to (010)	49.79	0.66	5.28	0.09	12.05	0.35	12.18	0.45	0.18	0.04	4.59	0.21	7.80	0.19	1.99	0.12	1.19	0.08	0.81	0.04	95.85	24.8
**KI 149**	2	Parallel to (010)	49.22	0.65	5.24	0.08	12.18	0.35	12.23	0.46	0.21	0.04	4.49	0.20	7.80	0.19	1.90	0.12	1.24	0.08	0.80	0.04	95.30	26.9
**KI 149**	2	Parallel to (010)	49.05	0.65	5.23	0.08	11.83	0.35	12.18	0.45	0.21	0.04	4.30	0.20	7.95	0.19	1.90	0.12	1.16	0.08	0.78	0.04	94.59	29.0
**KI 149**	3	Perp to (010)	50.21	0.67	0.12	0.03	29.99	0.54	0.81	0.14	-	-	-	-	13.26	0.24	3.63	0.16	0.15	0.04	0.16	0.02	98.51	0.0
**KI 149**	3	Perp to (010)	50.56	0.67	0.13	0.03	29.44	0.53	0.90	0.14	-	-	-	-	12.88	0.24	3.89	0.17	0.16	0.04	0.15	0.03	98.28	3.4
**KI 149**	3	Perp to (010)	51.12	0.68	0.14	0.03	29.52	0.53	0.92	0.14	-	-	0.17	0.05	12.61	0.24	4.06	0.18	0.16	0.04	0.14	0.02	98.84	6.9
**KI 149**	3	Perp to (010)	50.95	0.67	0.52	0.04	28.17	0.52	1.78	0.18	-	-	0.44	0.07	12.38	0.24	4.02	0.17	0.17	0.04	0.22	0.03	98.65	10.3
**KI 149**	3	Perp to (010)	49.34	0.66	5.34	0.05	11.73	0.49	12.42	0.24	0.19	0.04	4.53	0.10	7.74	0.22	2.59	0.17	1.20	0.05	0.80	0.03	95.88	13.7
**KI 149**	3	Perp to (010)	50.27	0.64	5.29	0.06	12.39	0.45	12.25	0.31	0.20	0.04	4.52	0.13	7.90	0.21	2.63	0.16	1.17	0.06	0.79	0.03	97.41	17.2
**KI 149**	3	Perp to (010)	50.08	0.63	5.23	0.07	12.30	0.41	12.12	0.35	0.17	0.04	4.43	0.15	7.79	0.20	2.46	0.16	1.20	0.06	0.82	0.03	96.60	20.6
**KI 149**	3	Perp to (010)	50.08	0.61	5.22	0.08	12.51	0.38	12.09	0.37	0.16	0.04	4.40	0.16	7.85	0.18	2.47	0.15	1.20	0.07	0.82	0.03	96.82	24.0
**KI 149**	3	Perp to (010)	50.38	0.58	5.25	0.09	12.36	0.31	12.01	0.42	0.20	0.04	4.48	0.18	7.90	0.17	2.36	0.13	1.21	0.07	0.81	0.03	96.96	27.5
**KI 149**	3	Perp to (010)	50.05	0.66	5.23	0.10	12.30	0.35	12.28	0.46	0.17	0.05	4.32	0.20	8.02	0.18	2.44	0.14	1.12	0.08	0.81	0.04	96.75	30.9
**KI 149**	3	Perp to (010)	49.81	0.66	5.25	0.10	12.21	0.35	12.20	0.46	0.18	0.04	4.41	0.21	7.96	0.19	2.41	0.14	1.13	0.08	0.80	0.04	96.36	34.4
**KI 149**	3	Perp to (010)	50.02	0.66	5.23	0.10	12.28	0.35	12.51	0.46	0.18	0.04	4.41	0.20	7.89	0.19	2.53	0.14	1.16	0.08	0.79	0.04	97.01	37.8
**KI 149**	3	Perp to (010)	50.12	0.66	5.30	0.10	12.17	0.36	12.30	0.45	0.19	0.04	4.38	0.20	7.89	0.19	2.62	0.14	1.14	0.08	0.79	0.04	96.91	41.2

**KI 149**	3	Perp to (010)	50.05	0.66	5.30	0.10	11.96	0.35	12.45	0.45	0.17	0.04	4.44	0.20	7.90	0.19	2.48	0.13	1.15	0.08	0.80	0.04	96.70	44.7
**KI 149**	3	Perp to (010)	49.82	0.66	5.28	0.10	12.17	0.35	12.45	0.46	0.19	0.04	4.28	0.20	7.99	0.19	2.51	0.14	1.14	0.08	0.81	0.04	96.64	48.1
**KI 149**	3	Perp to (010)	49.83	0.66	5.24	0.10	12.14	0.35	12.27	0.46	0.19	0.04	4.46	0.20	7.99	0.19	2.43	0.14	1.13	0.08	0.80	0.04	96.50	51.5
**KI 149**	3	Perp to (010)	49.87	0.66	5.27	0.10	12.17	0.35	12.25	0.46	0.18	0.04	4.38	0.20	7.97	0.19	2.56	0.14	1.13	0.08	0.79	0.04	96.57	55.0
**KI 149**	3	Perp to (010)	49.47	0.66	5.25	0.10	11.86	0.35	12.34	0.46	0.16	0.04	4.39	0.20	7.61	0.19	2.50	0.14	1.08	0.08	0.79	0.04	95.45	58.4
**KI 149**	3	Perp to (010)	48.88	0.66	5.10	0.10	11.82	0.35	12.14	0.46	0.21	0.04	4.40	0.21	7.52	0.19	2.42	0.14	1.07	0.08	0.77	0.04	94.32	61.8
**KI 149**	3	Perp to (010)	49.72	0.66	5.24	0.10	12.44	0.35	12.07	0.46	0.19	0.04	4.45	0.20	7.83	0.19	2.65	0.14	1.09	0.08	0.79	0.04	96.46	65.3
**KI 149**	3	Perp to (010)	50.33	0.66	5.19	0.10	11.46	0.35	12.15	0.45	0.20	0.04	4.83	0.20	8.26	0.19	2.53	0.14	1.05	0.08	0.76	0.04	96.76	68.7
**KI 149**	3	Perp to (010)	50.33	0.66	4.44	0.10	10.63	0.35	11.09	0.46	0.16	0.04	7.21	0.20	10.30	0.19	2.03	0.14	0.94	0.08	0.68	0.04	97.81	72.1
**KI 149**	3	Perp to (010)	50.03	0.66	3.93	0.10	7.73	0.35	10.30	0.46	0.20	0.04	9.83	0.20	13.08	0.18	1.37	0.14	0.59	0.08	0.60	0.04	97.66	75.6
**KI 149**	3	Perp to (010)	49.90	0.65	1.92	0.09	4.53	0.35	8.34	0.45	0.19	0.04	14.33	0.20	17.50	0.18	0.56	0.14	0.23	0.08	0.34	0.03	97.83	79.0
**KI 149**	3	Perp to (010)	50.24	0.65	1.54	0.09	2.49	0.35	7.95	0.45	0.20	0.04	15.68	0.20	18.60	0.18	0.32	0.14	0.02	0.08	0.28	0.04	97.33	82.4
**KI 149**	3	Perp to (010)	50.11	0.65	1.08	0.10	2.61	0.35	7.07	0.45	0.18	0.04	16.29	0.20	19.65	0.18	0.28	0.14	0.02	0.08	0.23	0.03	97.54	85.9
**KI 149**	3	Perp to (010)	49.83	0.66	1.04	0.10	2.76	0.36	6.66	0.45	0.16	0.04	16.26	0.20	19.87	0.19	0.30	0.14	0.01	0.08	0.22	0.04	97.11	89.3
**KI 149**	3	Perp to (010)	49.87	0.66	0.99	0.09	2.88	0.34	6.54	0.45	-	0.04	16.27	0.21	19.97	0.19	0.33	0.14	-	0.08	0.24	0.04	97.22	92.7
**KI 147**	1	Perp to (010)	53.77	2.20	4.01	0.23	12.62	0.60	11.74	0.96	0.17	0.08	3.76	0.32	7.02	0.39	3.18	0.38	1.61	0.13	0.91	0.07	98.79	0.0
**KI 147**	1	Perp to (010)	53.36	2.18	4.07	0.23	12.50	0.60	12.02	0.98	0.19	0.08	3.77	0.32	6.95	0.38	3.00	0.37	1.63	0.13	0.91	0.07	98.40	2.0
**KI 147**	1	Perp to (010)	54.03	2.20	3.98	0.23	12.43	0.59	11.76	0.97	0.18	0.08	3.61	0.31	6.91	0.38	2.39	0.33	1.58	0.13	0.87	0.07	97.74	3.9
**KI 147**	1	Perp to (010)	54.76	2.22	4.07	0.23	12.36	0.60	11.39	0.95	0.18	0.08	3.50	0.31	6.86	0.38	2.97	0.36	1.60	0.13	0.91	0.07	98.60	5.9
**KI 147**	1	Perp to (010)	53.60	2.19	4.07	0.23	12.24	0.59	12.05	0.98	0.19	0.08	3.63	0.32	7.24	0.39	3.32	0.39	1.52	0.13	0.89	0.07	98.76	7.9
**KI 147**	1	Perp to (010)	55.28	2.24	4.11	0.23	12.36	0.59	12.38	1.00	0.21	0.08	3.63	0.31	7.01	0.38	2.18	0.31	1.55	0.13	0.94	0.07	99.64	9.8
**KI 147**	1	Perp to (010)	54.47	2.21	4.09	0.23	12.70	0.60	12.08	0.98	0.31	0.08	3.84	0.32	6.96	0.38	3.09	0.38	1.58	0.13	0.95	0.07	100.06	11.8
**KI 147**	1	Perp to (010)	53.75	2.19	4.03	0.23	12.38	0.59	11.29	0.94	0.14	0.08	3.61	0.31	6.90	0.38	3.22	0.38	1.55	0.13	0.86	0.07	97.75	13.7
**KI 147**	1	Perp to (010)	54.37	2.21	4.00	0.23	12.61	0.60	12.51	1.01	0.18	0.08	3.74	0.32	6.97	0.38	2.85	0.36	1.55	0.13	0.92	0.07	99.68	15.7
**KI 147**	1	Perp to (010)	54.76	2.22	4.07	0.23	12.74	0.61	11.82	0.97	0.07	0.08	3.53	0.31	7.11	0.39	3.26	0.38	1.62	0.13	0.92	0.07	99.89	17.7
**KI 147**	1	Perp to (010)	54.98	2.23	4.06	0.23	12.49	0.60	11.53	0.95	0.17	0.08	3.43	0.30	6.91	0.38	2.43	0.33	1.54	0.13	0.91	0.07	98.45	19.6
**KI 147**	1	Perp to (010)	54.76	2.22	4.07	0.23	12.43	0.59	12.05	0.98	0.20	0.08	3.68	0.31	6.89	0.38	3.14	0.38	1.60	0.13	0.89	0.07	99.69	21.6
**KI 147**	1	Perp to (010)	54.04	2.20	3.98	0.23	12.99	0.61	12.12	0.98	0.21	0.09	3.69	0.31	6.80	0.38	3.02	0.37	1.60	0.13	0.96	0.07	99.40	23.6
**KI 147**	1	Perp to (010)	54.49	2.21	4.06	0.23	12.61	0.60	11.79	0.96	0.20	0.08	3.67	0.32	6.93	0.38	2.60	0.34	1.53	0.13	0.92	0.07	98.79	25.5
**KI 147**	1	Perp to (010)	54.52	2.22	3.98	0.23	12.81	0.61	11.90	0.97	0.13	0.08	3.47	0.30	7.03	0.39	3.20	0.38	1.58	0.13	0.93	0.07	99.54	27.5
**KI 147**	1	Perp to (010)	54.23	2.21	3.86	0.22	12.14	0.59	11.42	0.95	0.27	0.10	3.73	0.31	6.95	0.38	3.31	0.39	1.56	0.13	0.93	0.07	98.41	29.5
**KI 147**	1	Perp to (010)	53.72	2.19	3.91	0.22	12.59	0.60	12.24	0.99	0.22	0.09	3.48	0.31	6.72	0.38	2.63	0.34	1.66	0.13	0.91	0.07	98.08	31.4
**KI 147**	1	Perp to (010)	53.92	2.20	3.87	0.22	12.66	0.60	11.90	0.98	0.30	0.08	3.70	0.32	6.77	0.38	2.54	0.34	1.65	0.13	0.93	0.07	98.24	33.4
**KI 147**	2	Parallel to (010)	54.43	2.21	4.09	0.23	12.47	0.59	12.03	0.98	0.25	0.08	3.67	0.31	7.04	0.39	3.27	0.39	1.53	0.13	0.92	0.07	99.69	0.0
**KI 147**	2	Parallel to (010)	53.85	2.19	4.05	0.23	12.43	0.60	11.62	0.96	0.19	0.08	3.71	0.32	6.91	0.38	3.19	0.38	1.59	0.13	0.93	0.07	98.47	1.8
**KI 147**	2	Parallel to (010)	53.89	2.19	4.11	0.23	12.54	0.60	11.99	0.97	0.15	0.08	3.51	0.31	7.00	0.39	3.19	0.38	1.55	0.13	0.93	0.07	98.87	3.7
**KI 147**	2	Parallel to (010)	53.69	2.19	4.09	0.23	12.65	0.60	11.96	0.97	0.15	0.08	3.58	0.31	6.90	0.38	3.32	0.38	1.60	0.13	0.94	0.07	98.87	5.5
**KI 147**	2	Parallel to (010)	53.62	2.18	4.05	0.23	12.72	0.60	11.93	0.97	0.22	0.09	3.46	0.32	6.90	0.38	2.30	0.32	1.56	0.13	0.88	0.07	97.64	7.4
**KI 147**	2	Parallel to (010)	54.53	2.21	4.06	0.23	12.24	0.59	11.83	0.97	0.23	0.09	3.50	0.31	7.02	0.39	3.16	0.38	1.51	0.13	0.91	0.07	98.99	9.2
**KI 147**	2	Parallel to (010)	54.19	2.20	4.01	0.23	12.28	0.59	12.13	0.98	0.18	0.08	3.61	0.31	7.09	0.39	2.55	0.34	1.63	0.13	0.91	0.07	98.59	11.0
**KI 147**	2	Parallel to (010)	53.78	2.19	3.94	0.22	12.70	0.60	11.80	0.97	0.23	0.08	3.55	0.31	6.94	0.38	3.09	0.37	1.59	0.13	0.89	0.07	98.51	12.9
**KI 147**	2	Parallel to (010)	53.73	2.19	4.02	0.23	12.47	0.59	12.06	0.98	0.19	0.08	3.69	0.31	6.96	0.38	3.02	0.37	1.61	0.13	0.91	0.07	98.66	14.7
**KI 147**	2	Parallel to (010)	53.87	2.19	4.07	0.23	12.78	0.60	11.89	0.97	0.23	0.09	3.67	0.31	6.67	0.37	3.17	0.37	1.65	0.13	0.90	0.07	98.90	16.6
**KI 147**	2	Parallel to (010)	53.70	2.19	3.99	0.23	12.47	0.59	11.60	0.95	0.11	0.08	3.69	0.31	7.07	0.39	2.42	0.33	1.62	0.13	0.92	0.07	97.60	18.4
**KI 147**	2	Parallel to (010)	54.07	2.20	3.99	0.23	12.50	0.60	11.86	0.97	0.25	0.09	3.61	0.31	6.88	0.38	3.08	0.37	1.63	0.13	0.90	0.07	98.77	20.2
**KI 147**	2	Parallel to (010)	53.73	2.19	4.03	0.23	12.50	0.59	12.34	0.99	0.21	0.09	3.68	0.31	6.82	0.38	2.76	0.35	1.60	0.13	0.92	0.07	98.58	22.1
**KI 147**	2	Parallel to (010)	53.99	2.20	3.99	0.23	12.35	0.59	11.51	0.95	0.25	0.09	3.75	0.32	6.83	0.38	2.98	0.36	1.57	0.13	0.91	0.07	98.13	23.9
**KI 147**	2	Parallel to (010)	53.78	2.19	3.96	0.22	12.74	0.60	12.20	0.99	0.24	0.09	3.74	0.32	7.11	0.39	3.20	0.38	1.60	0.13	0.91	0.07	99.48	25.8
**KI 147**	2	Parallel to (010)	54.05	2.20	3.89	0.22	12.63	0.60	11.74	0.96	0.27	0.10	3.48	0.30	6.85	0.38	2.73	0.35	1.67	0.13	0.91	0.07	98.21	27.6

**KI 147**	2	Parallel to (010)	54.83	2.22	3.88	0.22	12.55	0.60	11.96	0.97	0.20	0.09	3.45	0.30	6.99	0.38	2.53	0.33	1.59	0.13	0.88	0.07	98.85	29.4
**KI 147**	2	Parallel to (010)	53.42	2.18	3.83	0.22	12.64	0.60	11.55	0.95	0.17	0.08	3.58	0.31	6.74	0.38	3.22	0.38	1.66	0.13	0.90	0.07	97.70	31.3
**KI 147**	2	Parallel to (010)	53.96	2.19	3.83	0.22	12.31	0.59	11.51	0.95	0.24	0.09	3.73	0.31	6.74	0.38	2.97	0.36	1.71	0.14	0.91	0.07	97.91	33.1
**KI 147**	2	Parallel to (010)	53.37	2.18	3.74	0.21	11.49	0.57	12.09	0.98	0.17	0.09	3.78	0.32	6.47	0.37	2.62	0.34	1.68	0.13	0.89	0.07	96.31	35.0
**KI 147**	2	Parallel to (010)	55.96	2.25	4.11	0.23	10.81	0.55	12.47	1.00	0.10	0.09	3.90	0.33	6.59	0.37	2.21	0.31	1.72	0.14	0.96	0.07	98.82	36.8
**KI 147**	3	Parallel to (010)	54.97	2.23	3.85	0.22	12.63	0.60	12.19	0.99	0.21	0.09	3.78	0.32	6.92	0.38	3.07	0.38	1.66	0.13	0.92	0.07	100.20	0.0
**KI 147**	3	Parallel to (010)	54.37	2.21	3.97	0.23	12.79	0.60	11.97	0.97	0.27	0.10	3.52	0.31	6.99	0.38	2.17	0.31	1.57	0.13	0.90	0.07	98.51	1.7
**KI 147**	3	Parallel to (010)	54.15	2.20	3.82	0.22	12.29	0.59	11.85	0.97	0.20	0.08	3.96	0.32	6.62	0.37	2.53	0.33	1.62	0.13	0.91	0.07	97.95	3.4
**KI 147**	3	Parallel to (010)	54.25	2.21	3.88	0.22	12.80	0.61	11.80	0.96	0.20	0.08	3.60	0.31	6.84	0.38	3.12	0.38	1.61	0.13	0.92	0.07	99.03	5.1
**KI 147**	3	Parallel to (010)	54.55	2.21	3.85	0.22	12.50	0.60	11.65	0.96	0.12	0.05	3.86	0.32	6.79	0.38	3.21	0.38	1.63	0.13	0.95	0.07	99.09	6.8
**KI 147**	3	Parallel to (010)	53.76	2.19	3.90	0.22	12.66	0.60	11.70	0.96	0.13	0.05	3.59	0.31	6.94	0.38	3.26	0.38	1.62	0.13	0.92	0.07	98.47	8.5
**KI 147**	3	Parallel to (010)	55.71	2.25	3.92	0.22	12.44	0.59	11.76	0.96	0.24	0.09	3.77	0.32	7.07	0.39	2.04	0.30	1.50	0.13	0.89	0.07	99.34	10.1
**KI 147**	3	Parallel to (010)	55.15	2.23	3.83	0.22	12.72	0.60	11.45	0.94	-	-	3.83	0.32	6.77	0.38	2.69	0.34	1.64	0.13	0.94	0.07	99.04	11.8
**KI 147**	3	Parallel to (010)	54.08	2.20	3.92	0.22	12.43	0.60	11.61	0.96	0.24	0.09	3.39	0.30	6.95	0.38	2.73	0.34	1.69	0.14	0.91	0.07	97.97	13.5
**KI 147**	3	Parallel to (010)	53.75	2.19	3.85	0.22	12.81	0.60	11.52	0.95	0.19	0.08	3.87	0.32	6.83	0.38	3.30	0.39	1.64	0.13	0.89	0.07	98.64	15.2
**KI 147**	3	Parallel to (010)	53.96	2.20	3.92	0.22	12.62	0.60	11.73	0.97	0.19	0.08	3.61	0.31	6.88	0.38	3.11	0.37	1.62	0.13	0.94	0.07	98.58	16.9
**KI 147**	3	Parallel to (010)	54.60	2.22	3.97	0.22	12.53	0.60	12.00	0.98	0.19	0.08	3.64	0.31	6.81	0.38	2.22	0.31	1.47	0.13	0.93	0.07	98.36	18.6
**KI 147**	3	Parallel to (010)	54.54	2.21	3.88	0.22	12.63	0.60	12.10	0.98	0.21	0.08	3.65	0.31	6.80	0.38	2.64	0.34	1.62	0.13	0.93	0.07	98.99	20.3
**KI 147**	3	Parallel to (010)	53.85	2.20	3.93	0.22	12.80	0.61	11.65	0.96	0.25	0.09	3.65	0.31	6.94	0.38	3.24	0.38	1.57	0.13	0.92	0.07	98.80	22.0
**KI 147**	3	Parallel to (010)	54.27	2.21	3.93	0.22	12.49	0.60	11.81	0.97	-	-	3.56	0.31	6.65	0.37	3.28	0.38	1.65	0.13	0.94	0.07	98.63	23.7
**KI 147**	3	Parallel to (010)	54.15	2.21	3.86	0.22	12.59	0.60	12.23	0.99	0.18	0.08	3.65	0.31	6.79	0.38	3.08	0.37	1.56	0.13	0.91	0.07	99.00	25.4
**KI 147**	3	Parallel to (010)	54.51	2.22	3.91	0.22	12.73	0.60	12.30	0.99	0.18	0.08	3.56	0.31	6.83	0.38	2.34	0.32	1.55	0.13	0.91	0.07	98.81	27.0
**KI 147**	3	Parallel to (010)	54.38	2.21	3.82	0.22	12.36	0.60	11.69	0.96	0.24	0.09	3.45	0.30	7.03	0.39	2.53	0.34	1.60	0.13	0.91	0.07	98.01	28.7
**KI 147**	3	Parallel to (010)	54.53	2.21	3.92	0.22	12.42	0.59	11.60	0.96	0.09	0.04	3.54	0.31	6.99	0.38	2.94	0.36	1.61	0.13	0.90	0.07	98.54	30.4
**KI 147**	3	Parallel to (010)	53.94	2.20	4.01	0.23	12.71	0.60	11.67	0.96	0.23	0.09	3.65	0.31	6.81	0.38	3.17	0.38	1.59	0.13	0.92	0.07	98.70	32.1
**KI 147**	3	Parallel to (010)	54.57	2.22	3.93	0.22	12.73	0.60	12.24	0.99	0.25	0.09	3.58	0.31	6.93	0.38	3.32	0.39	1.68	0.13	0.93	0.07	100.16	33.8
**KI 147**	3	Parallel to (010)	54.88	2.22	3.99	0.23	12.71	0.60	11.64	0.96	0.15	0.06	3.51	0.31	6.81	0.38	2.05	0.30	1.53	0.13	0.95	0.07	98.22	35.5
**KI 147**	3	Parallel to (010)	55.05	2.23	3.90	0.22	12.78	0.60	11.87	0.97	0.18	0.06	3.52	0.31	6.77	0.38	2.81	0.35	1.57	0.13	0.94	0.07	99.38	37.2
**KI 147**	3	Parallel to (010)	55.08	2.23	3.94	0.22	12.81	0.60	12.45	1.00	0.09	0.04	3.66	0.31	7.02	0.39	3.05	0.37	1.62	0.13	0.90	0.07	100.63	38.9
**KI 147**	3	Parallel to (010)	54.19	2.20	3.99	0.23	12.42	0.59	11.83	0.97	0.24	0.09	3.68	0.31	6.87	0.38	2.25	0.31	1.58	0.13	0.92	0.07	97.97	40.6
**KI 147**	3	Parallel to (010)	53.70	2.19	3.87	0.22	12.66	0.60	11.81	0.97	0.18	0.08	3.61	0.31	6.99	0.38	3.27	0.39	1.62	0.13	0.90	0.07	98.62	42.3
**KI 147**	3	Parallel to (010)	54.79	2.22	3.98	0.23	12.76	0.60	11.46	0.95	0.09	0.04	3.69	0.31	6.77	0.38	2.38	0.32	1.56	0.13	0.89	0.07	98.38	43.9
**KI 147**	3	Parallel to (010)	54.28	2.21	3.91	0.22	12.57	0.60	12.12	0.99	0.22	0.09	3.56	0.31	6.61	0.37	3.20	0.38	1.62	0.13	0.87	0.07	98.96	45.6
**KI 147**	3	Parallel to (010)	54.28	2.21	3.93	0.22	12.69	0.60	12.02	0.98	0.06	0.04	3.55	0.31	6.94	0.38	2.15	0.30	1.50	0.13	0.94	0.07	98.06	47.3
**KI 147**	3	Parallel to (010)	54.06	2.20	3.88	0.22	12.80	0.61	11.91	0.98	0.23	0.09	3.65	0.31	6.81	0.38	3.17	0.38	1.62	0.13	0.92	0.07	99.04	49.0
**KI 147**	3	Parallel to (010)	55.19	2.24	3.93	0.22	12.71	0.60	11.88	0.97	0.20	0.09	3.57	0.31	6.82	0.38	2.40	0.32	1.55	0.13	0.92	0.07	99.18	50.7
**KI 147**	3	Parallel to (010)	54.91	2.22	3.96	0.22	12.48	0.60	11.78	0.97	0.30	0.10	3.70	0.31	6.74	0.38	3.03	0.37	1.59	0.13	0.86	0.07	99.33	52.4
**KI 147**	3	Parallel to (010)	54.83	2.22	3.89	0.22	12.63	0.60	11.94	0.98	0.28	0.09	3.41	0.30	6.61	0.37	2.94	0.36	1.64	0.13	0.92	0.07	99.07	54.1
**KI 147**	3	Parallel to (010)	53.84	2.19	3.97	0.22	12.68	0.60	12.06	0.98	0.21	0.09	3.58	0.31	6.75	0.38	3.06	0.37	1.60	0.13	0.91	0.07	98.64	55.8
**KI 147**	3	Parallel to (010)	53.85	2.19	4.01	0.23	12.77	0.60	11.60	0.96	0.22	0.09	3.22	0.29	6.89	0.38	3.41	0.40	1.58	0.13	0.94	0.07	98.51	57.5
**KI 147**	3	Parallel to (010)	54.80	2.22	3.99	0.23	12.94	0.61	11.95	0.97	0.08	0.04	3.27	0.31	6.46	0.37	3.07	0.37	1.70	0.14	0.93	0.07	99.18	59.2
**KI 147**	3	Parallel to (010)	54.56	2.21	4.08	0.23	12.84	0.60	11.39	0.95	0.15	0.08	3.09	0.29	6.62	0.37	2.18	0.31	1.63	0.13	0.97	0.07	97.52	60.8
**KI 147**	3	Parallel to (010)	54.80	2.22	3.87	0.22	13.26	0.62	10.65	0.90	0.13	0.06	3.02	0.28	6.41	0.36	2.73	0.35	1.80	0.14	0.96	0.07	97.63	62.5
**KI 147**	3	Parallel to (010)	55.43	2.24	3.98	0.23	13.44	0.62	10.84	0.91	0.18	0.09	2.97	0.28	6.26	0.36	1.98	0.29	1.77	0.14	0.98	0.07	97.83	64.2
**KI 147**	4	Parallel to (010)	54.82	2.22	3.92	0.22	12.50	0.60	11.61	0.96	0.18	0.08	3.81	0.32	6.89	0.38	3.02	0.37	1.64	0.13	0.95	0.07	99.32	0.0
**KI 147**	4	Parallel to (010)	55.06	2.23	3.92	0.22	12.94	0.61	11.66	0.96	0.12	0.06	3.69	0.31	6.92	0.38	2.28	0.31	1.57	0.13	0.95	0.07	99.10	1.5
**KI 147**	4	Parallel to (010)	54.51	2.21	3.94	0.22	12.49	0.60	11.57	0.95	0.23	0.08	3.64	0.31	6.89	0.38	3.03	0.37	1.65	0.13	0.88	0.07	98.81	3.0
**KI 147**	4	Parallel to (010)	54.48	2.21	3.97	0.22	12.62	0.60	11.56	0.95	0.19	0.08	3.80	0.32	7.09	0.39	2.75	0.35	1.68	0.13	0.93	0.07	99.06	4.5
**KI 147**	4	Parallel to (010)	54.59	2.21	3.88	0.22	12.67	0.60	11.97	0.97	0.20	0.08	3.53	0.30	6.89	0.38	2.31	0.32	1.55	0.13	0.87	0.07	98.46	6.0

**KI 147**	4	Parallel to (010)	54.91	2.23	3.92	0.22	12.87	0.61	11.98	0.98	0.15	0.05	3.48	0.30	6.82	0.38	3.18	0.38	1.59	0.13	0.89	0.07	99.78	7.6
**KI 147**	4	Parallel to (010)	54.03	2.20	3.87	0.22	12.80	0.60	12.03	0.98	0.07	0.04	3.50	0.30	7.00	0.39	3.18	0.38	1.57	0.13	0.91	0.07	98.95	9.1
**KI 147**	4	Parallel to (010)	54.58	2.22	4.03	0.23	12.56	0.60	12.33	0.99	0.34	0.12	3.57	0.30	7.02	0.38	2.12	0.30	1.54	0.13	0.96	0.07	99.05	10.6
**KI 147**	4	Parallel to (010)	54.72	2.22	3.90	0.22	12.76	0.60	11.90	0.97	0.15	0.06	3.52	0.30	6.72	0.38	2.41	0.33	1.67	0.13	0.90	0.07	98.63	12.1
**KI 147**	4	Parallel to (010)	54.37	2.21	3.96	0.22	12.64	0.60	12.03	0.98	0.23	0.08	3.57	0.31	7.02	0.38	3.31	0.39	1.61	0.13	0.92	0.07	99.65	13.6
**KI 147**	4	Parallel to (010)	54.67	2.22	3.87	0.22	12.68	0.60	12.32	0.99	0.22	0.08	3.45	0.31	6.94	0.38	3.09	0.37	1.68	0.14	0.93	0.07	99.84	15.1
**KI 147**	4	Parallel to (010)	54.88	2.22	3.92	0.22	12.64	0.60	11.99	0.98	0.10	0.04	3.63	0.31	6.96	0.38	2.12	0.30	1.54	0.13	0.93	0.07	98.71	16.6
**KI 147**	4	Parallel to (010)	55.02	2.23	3.99	0.23	12.50	0.59	11.73	0.96	0.24	0.09	3.59	0.31	6.92	0.38	2.08	0.30	1.58	0.13	0.90	0.07	98.55	18.1
**KI 147**	4	Parallel to (010)	53.85	2.19	3.82	0.22	12.24	0.59	11.90	0.97	0.26	0.08	3.60	0.31	6.73	0.38	2.92	0.36	1.56	0.13	0.94	0.07	97.83	19.6
**KI 147**	4	Parallel to (010)	53.86	2.19	3.92	0.22	12.68	0.60	11.72	0.96	0.22	0.09	3.51	0.31	6.79	0.38	2.89	0.36	1.67	0.13	0.94	0.07	98.22	21.1
**KI 147**	4	Parallel to (010)	54.99	2.23	3.93	0.22	12.53	0.60	11.79	0.96	0.16	0.08	3.78	0.32	6.87	0.38	2.96	0.36	1.61	0.13	0.96	0.07	99.58	22.7
**KI 147**	4	Parallel to (010)	54.67	2.22	4.03	0.23	11.16	0.56	12.53	1.01	0.22	0.08	3.94	0.32	6.85	0.38	2.18	0.31	1.73	0.14	0.96	0.07	98.27	24.2
**KI 147**	5	Perp to (010)	54.40	2.21	3.86	0.22	12.60	0.60	11.75	0.96	0.18	0.08	3.82	0.32	6.96	0.38	3.32	0.39	1.65	0.13	0.88	0.07	99.42	0.0
**KI 147**	5	Perp to (010)	54.83	2.22	3.88	0.22	12.54	0.60	11.80	0.96	-	-	3.54	0.31	6.99	0.38	2.35	0.32	1.56	0.13	0.90	0.07	98.39	1.0
**KI 147**	5	Perp to (010)	54.50	2.21	3.92	0.22	12.62	0.60	11.95	0.98	0.15	0.08	3.67	0.31	7.01	0.38	2.84	0.35	1.68	0.13	0.89	0.07	99.22	2.0
**KI 147**	5	Perp to (010)	54.84	2.22	3.98	0.23	13.01	0.61	12.12	0.98	0.20	0.09	3.72	0.32	6.88	0.38	2.99	0.36	1.56	0.13	0.93	0.07	100.23	3.0
**KI 147**	5	Perp to (010)	54.07	2.20	4.00	0.23	12.58	0.60	11.91	0.97	0.05	0.02	3.63	0.31	6.96	0.38	2.29	0.32	1.61	0.13	0.89	0.07	98.00	4.0
**KI 147**	5	Perp to (010)	54.25	2.21	3.86	0.22	12.48	0.60	11.71	0.96	0.24	0.09	3.63	0.31	6.77	0.38	3.14	0.38	1.65	0.13	0.90	0.07	98.64	5.0
**KI 147**	5	Perp to (010)	54.22	2.21	3.87	0.22	12.66	0.60	11.63	0.95	0.11	0.05	3.41	0.30	6.73	0.38	2.48	0.33	1.63	0.13	0.93	0.07	97.68	6.0
**KI 147**	5	Perp to (010)	54.68	2.22	3.93	0.22	12.48	0.60	11.83	0.97	0.13	0.05	3.61	0.31	6.83	0.38	3.08	0.37	1.64	0.13	0.87	0.07	99.09	7.0
**KI 147**	5	Perp to (010)	55.02	2.23	3.90	0.22	12.57	0.60	11.73	0.96	0.12	0.05	3.65	0.31	7.04	0.39	2.49	0.33	1.67	0.13	0.93	0.07	99.11	8.0
**KI 147**	5	Perp to (010)	54.69	2.22	3.87	0.22	12.51	0.60	11.72	0.96	0.19	0.08	3.53	0.31	6.99	0.38	2.67	0.35	1.61	0.13	0.92	0.07	98.69	9.0
**KI 147**	5	Perp to (010)	54.19	2.20	3.92	0.22	12.52	0.59	11.60	0.96	0.28	0.09	3.60	0.31	6.98	0.38	1.96	0.30	1.60	0.13	0.89	0.07	97.54	10.0
**KI 147**	5	Perp to (010)	54.78	2.22	3.98	0.23	12.74	0.60	12.26	0.99	0.28	0.09	3.34	0.30	7.02	0.39	2.41	0.33	1.66	0.13	0.92	0.07	99.39	11.0
**KI 147**	5	Perp to (010)	55.07	2.23	3.98	0.23	12.42	0.59	11.46	0.95	0.13	0.04	3.70	0.31	7.08	0.39	2.44	0.33	1.57	0.13	0.91	0.07	98.75	12.0
**KI 147**	5	Perp to (010)	54.24	2.21	3.88	0.22	12.60	0.60	12.02	0.98	0.19	0.08	3.59	0.31	6.83	0.38	3.13	0.37	1.59	0.13	0.93	0.07	99.01	13.0
**KI 147**	5	Perp to (010)	54.99	2.23	3.87	0.22	12.82	0.60	11.99	0.98	0.24	0.09	3.64	0.31	6.95	0.38	2.03	0.30	1.55	0.13	0.93	0.07	99.01	14.0
**KI 147**	5	Perp to (010)	55.01	2.23	3.80	0.22	15.07	0.67	12.12	0.98	0.15	0.04	3.16	0.29	7.65	0.41	3.69	0.41	1.18	0.11	0.88	0.07	102.71	15.0
**KI 147**	5	Perp to (010)	53.44	2.19	0.28	0.05	27.60	0.96	0.87	0.26	-	-	0.27	0.10	11.62	0.54	4.58	0.45	0.27	0.05	0.20	0.04	99.12	16.0

Table 4 Bulk chemical analyses for the specified phase from APT tips. Note that compositions are listed as atomic percent (at. %).

Sample	Si	Ti	Al	Fe	Mn	Mg	Ca	Na	K	P	O	Total
**Average bulk analyses from the Plagioclase, Concentration (at %)**												
**178414**	24.8	0.2	11.4	1.7	-	0.3	2.7	2.5	0.1	0.0	56.2	99.9
**179197**	23.0	0.0	12.6	2.6	0.0	0.6	3.2	3.0	0.2	0.1	53.6	98.9
**179237**	25.1	-	10.8	2.0	0.0	0.5	3.0	1.6	0.1	0.1	56.8	99.8
**179377**	23.8	0.0	11.0	1.9	0.0	0.5	3.0	2.1	0.1	0.0	57.5	99.9
**173738**	20.7	0.0	15.5	0.7	0.0	0.1	5.3	2.0	0.1	0.0	55.7	100.0
**Average bulk analyses from the glass Compositional Boundary Layer, Concentration (at %)**												
**178414**	17.6	0.8	2.1	12.6	0.2	7.8	7.3	0.4	0.0	1.5	49.7	99.9
**179197**	16.2	0.7	2.3	13.6	0.2	7.2	6.2	0.4	0.0	1.2	51.8	99.8
**179237**	17.2	0.7	2.2	13.0	0.2	7.9	6.7	0.3	0.0	1.6	50.1	100.0
**179377**	15.2	0.8	2.2	13.4	0.2	7.7	7.1	0.3	0.0	1.5	51.6	100.0
**179526**	15.7	0.7	2.4	13.5	0.2	7.9	7.1	0.5	0.0	1.2	50.9	100.0
**173970**	17.2	0.7	1.8	12.8	0.2	8.9	8.0	0.3	0.0	1.9	47.6	99.5
**Average bulk analyses from the Fe-rich glass comprising the nanoemulsion, Concentration (at %)**												
**179523**	16.5	1.5	3.9	12.3	0.12	9.2	7.4	0.3	0.1	0.9	47.4	99.7
**179530**	16.3	1.2	4.3	11.8	0.10	8.9	7.8	0.7	0.1	0.5	48.0	99.8
**179484**	16.6	1.4	4.1	11.5	0.16	8.5	7.8	0.8	0.1	1.4	47.6	99.8
**179483**	15.5	1.2	4.7	12.0	0.10	8.5	8.1	1.2	0.2	0.8	47.3	99.6
**179534**	16.3	1.2	4.0	11.6	0.06	9.2	8.3	0.6	0.1	0.4	47.5	99.1
**173941**	18.2	1.1	5.3	10.7	0.21	7.6	7.5	0.8	0.1	1.4	46.3	99.2
**173974**	18.4	1.4	5.5	10.5	0.04	7.6	6.4	0.5	0.1	1.4	47.6	99.5
**173975**	21.9	0.8	4.6	17.8	0.03	3.0	2.1	1.0	0.2	1.8	46.6	99.7
**173967**	20.7	0.9	5.5	10.8	0.10	6.1	5.8	0.6	0.1	1.5	48.1	100.1
**173972**	16.8	0.8	6.3	14.3	0.16	5.4	5.6	0.9	0.2	0.3	48.9	99.7
**Average bulk analyses from the Si-rich glass comprising the nanoemulsion, Concentration (at %)**												
**179523**	28.1	0.1	8.4	2.2	0.0	1.1	1.2	0.1	0.3	0.2	58.1	99.8
**179530 (1)**	27.4	0.1	8.1	2.3	0.0	0.7	0.9	1.5	0.7	0.1	57.8	99.6
**179484 (1)**	29.2	-	6.7	1.6	0.0	0.4	0.8	0.5	0.2	0.1	60.3	99.9
**179522 (1)**	28.7	0.0	7.5	2.1	0.0	0.7	0.8	1.7	0.6	0.1	57.5	99.6
**179483**	28.1	0.0	7.7	2.0	0.0	0.7	0.9	0.8	0.5	0.1	59.1	100.0
**179534**	28.5	0.0	8.3	2.2	0.0	0.7	0.9	2.3	0.7	0.1	56.1	99.9
**179530 (2)**	28.0	0.1	8.4	2.2	0.0	0.6	1.0	1.8	0.6	0.1	57.2	100.0
**179484 (2)**	28.1	0.2	7.9	2.1	0.0	0.7	1.1	0.5	0.4	0.0	58.9	100.0
**179522 (2)**	28.5	0.1	7.4	1.9	0.0	0.6	0.8	1.9	0.4	0.1	56.6	98.4
**173941**	26.2	0.1	8.9	2.3	0.0	0.9	1.4	1.4	0.5	0.1	57.6	99.4
**173974**	26.1	0.0	9.0	2.4	0.0	1.1	1.7	1.2	0.4	0.1	57.4	99.5
**173773**	26.6	0.0	8.9	2.1	0.0	0.8	1.6	1.1	0.4	0.2	58.3	100.0
**173975**	25.8	0.0	9.1	2.2	0.0	1.2	1.7	0.9	0.4	0.0	58.1	99.4
**173967**	25.3	0.0	8.9	2.5	0.0	1.2	1.8	1.5	0.5	0.2	57.5	99.3
**173972**	26.2	0.0	8.8	2.4	0.0	1.1	1.8	0.8	0.3	0.0	57.8	99.3
**173770**	26.5	0.0	8.8	2.3	0.0	0.8	1.4	0.9	0.3	0.1	58.8	100.0

SUPPLEMENTARY NOTE 1

Kīlauea Iki Lava Lake, Hawaii, USA

The Kīlauea Iki lava lake formed following the 14 November 1959 summit eruption[10]. The lava ponded in a crater, forming a closed magma system of $40x10^6$ m^3. The lava lake was successively sampled through its 35 year crystallisation history. Much of the drilling was done through partially molten rock, meaning cooling water was pumped down alongside the drill bit to quench the samples[11]. The samples studied are from the 1976-1, 1979-1, 1981, and 1988-2 drill cores (Table 1), which are 0.06m diameter and obtained using diamond bits. Coring resulted in 99% recovery (Table 2). The Kīlauea Iki lava lake samples chosen to characterise the Fe-rich compositional boundary layer (Fe-rich CBL) are cut from (or very close to) the base of each drill section, where the glass content was highest.

Table 1. *Polished sections studied from the Kīlauea Iki lava lake (Y = sample was analysed with this technique)*

Sample	Sample depth (m)	SEM	EPMA	APT
KI76 123	37.6	Y		
KI76 135	41.1	Y		
KI76 140	42.8	Y	Y	
KI76 143	43.7	Y	Y	
KI76 145	44.3	Y	Y	
KI76 147	44.8	Y	Y	Y
KI76 149	45.5	Y	Y	
KI79 197.3	60.1	Y		
KI79 202.0	61.6	Y		
KI81 192.9	58.8	Y	Y	
KI81 205.4	62.6	Y	Y	
KI81 219.8	67.0	Y	Y	
KI81 249.7	76.1	Y	Y	
KI88 266.6	81.3	Y		
KI88 300.0	91.4	Y		
KI88 336.3	102.5	Y		
KI88 354.9	108.2	Y		

Table 2. *1976-1 drill core drilling schedule over the length of core studied (Helz 2018 pers. comm.)*

Depth (m)	Core recovered (m)	Comments
32.0 – 35.1	3.0	-
35.1 – 38.1	3.0	-
38.1 – 41.1	3.0	-
41.1 – 42.8	1.6	KI 140 sample is from the bottom of this core.
42.8 – 44.3	1.5	KI 145 sample is from the bottom of this core.
44.3 – 45.5	1.2	KI 149 sample is from the bottom of this core.
45.5 – 46.0	-	Fragmental glass below 45.5 m.

Laki Lava Flow, Iceland

The Laki polished section (LAK04) is from episodes VI–X of the 1783–1784 CE basaltic Laki eruption in Iceland, for which the lava flow discharged from fissure VII. It was collected at c. 15 km from the source vent, and underwent sufficiently fast cooling rate to form a glassy rind[12,13].

Snake River Plain (SRP) Basalt, USA

The samples studied are from the Snake River volcanic province in southern Idaho, USA, from the Sugar city drill core through the basalt flows[14,15]. These flows contain many glassy horizons.

Table 3. *Polished sections studied from the Snake River Plain tholeiites (Y = sample was analysed with this technique)*

Sample depth from core top (m)	SEM	EPMA
90.8	Y	
91.3	Y	
91.5	Y	Y
91.7	Y	Y
95.9	Y	

SUPPLEMENTARY REFERENCES

1 Barth, G., Kleinrock, M. & Helz, R. The magma body at Kīlauea Iki lava lake: Potential insights into mid‐ocean ridge magma chambers. *Journal of Geophysical Research: Solid Earth* **99**, 7199-7217 (1994).

2 Helz, R. T., Clague, D. A., Sisson, T. W. & Thornber, C. R. in *Characteristics of Hawaiian volcanoes* (eds Michael P Poland, Taeko Jane Takahashi, & Claire M Landowski) Ch. 6, 237-292 (2014).

3 Charlier, B. & Grove, T. L. Experiments on liquid immiscibility along tholeiitic liquid lines of descent. *Contributions to Mineralogy and Petrology* **164**, 27-44 (2012).

4 Helz, R. T. Differentiation behavior of Kīlauea Iki lava lake, Kīlauea Volcano, Hawaii: an overview of past and current work. *Magmatic processes: physicochemical principles* **1**, 241-258 (1987).

5 Ryabov, V. V. Immiscibility in Natural Glasses, Russia, Novosibirsk, Ph.D Thesis, 221 pp, (1989).

6 Krasov, N. & Clocchiatti, R. Immiscibility in silicate melts and its possible petrogenetic importance, as shown by study of melt inclusions in *Transactions (Doklady) of the USSR Academy of Sciences*, 92-95 (1979).

7 Philpotts, A. Liquid immiscibility in silicate melt inclusions in plagioclase phenocrysts. *Bulletin de Minéralogie* **104**, 317-324 (1981).

8 Veksler, I. V., Dorfman, A. M., Borisov, A. A., Wirth, R. and Dingwell, D. B. Liquid immiscibility and the evolution of basaltic magma. *Journal of Petrology* **48**, 2187-2210 (2007).

9 Honour, V. C., Holness, M. B., Partridge, J. L. and Charlier, B. Microstructural evolution of silicate immiscible liquids in ferrobasalts. *Contributions to Mineralogy and Petrology* **174,** 77 (2019).

10 Morse, S. A. & Brady, J. B. Thermal History of the Upper Zone of the Kiglapait Intrusion. *Journal of Petrology* **58**, 1319-1332, doi:10.1093/petrology/egx055 (2017).

11 Kirkpatrick, R. J. Crystal growth from the melt: a review. *Am Mineral* **60**, 798-814 (1975).

12 Passmore, E., Maclennan, J., Fitton, G. & Thordarson, T. Mush Disaggregation in Basaltic Magma Chambers: Evidence from the AD 1783 Laki Eruption. *Journal of Petrology* **53**, 2593-2623, doi:10.1093/petrology/egs061 (2012).

13 Neave, D. A., Buisman, I. & Maclennan, J. Continuous mush disaggregation during the long-lasting Laki fissure eruption, Iceland. *American Mineralogist* **102**, 2007-2021, doi:10.2138/am-2017-6015CCBY (2017).

14 Embree, G. F., Lowell, M. D. & Doherty, D. J. Drilling data from Sugar City exploration well, Madison County, Idaho. Report No. 2331-1258, (1978).

15 Jean, M. M. *et al.* Caldera Life-Cycles of the Yellowstone Hotspot Track: Death and Rebirth of the Heise Caldera. *Journal of Petrology* **59**, 1643-1670, doi:10.1093/petrology/egy074 (2018).
