# Supplementary material for: Compositional boundary layers trigger liquid unmixing in a basaltic crystal mush
Source: Nat Commun. 2019 Oct 23;10:4821. doi: 10.1038/s41467-019-12694-5 (PMC6811629; doi:10.1038/s41467-019-12694-5)
Supplement: Supplementary file 3 — Description of Additional Supplementary Files [file 41467_2019_12694_MOESM3_ESM.pdf]

Description of Additional Supplementary Files

File name: Supplementary Movie 1
Description: Three-dimensional reconstruction of atom probe tomography data from the interstitial liquid adjacent to the compositional boundary layers around plagioclase.”
